# Caspase-8 scaffolding function and MLKL regulate NLRP3 inflammasome activation downstream of TLR3

**DOI:** 10.1038/ncomms8515

**Published:** 2015-06-24

**Authors:** Seokwon Kang, Teresa Fernandes-Alnemri, Corey Rogers, Lindsey Mayes, Ying Wang, Christopher Dillon, Linda Roback, William Kaiser, Andrew Oberst, Junji Sagara, Katherine A. Fitzgerald, Douglas R. Green, Jianke Zhang, Edward S. Mocarski, Emad S. Alnemri

**Affiliations:** 1 Department of Biochemistry and Molecular Biology, Kimmel Cancer Center, Thomas Jefferson University, Philadelphia, Pennsylvania 19107, USA; 2 Department of Physiology and Cellular Biophysics, Columbia University Medical Center, New York, New York 10032, USA; 3 Deptartment of Immunology, St Jude Children's Research Hospital, Memphis, Tennessee 38105, USA; 4 Department of Microbiology and Immunology, Emory Vaccine Center, Emory University School of Medicine, Atlanta, Georgia 30322, USA; 5 Department of Immunology, University of Washington, Seattle Washington 98109-8059 USA; 6 Department of Biomedical Laboratory Sciences, School of Health Sciences, Shinshu University, Matsumoto, Nagano 390-8621, Japan; 7 Division of Infectious Diseases and Immunology, University of Massachusetts Medical School, Worcester, Massachusetts 01605, USA; 8 Department of Microbiology and Immunology, Kimmel Cancer Center, Thomas Jefferson University, Philadelphia, Pennsylvania 19107, USA

## Abstract

TLR2 promotes NLRP3 inflammasome activation via an early MyD88-IRAK1-dependent pathway that provides a priming signal (signal 1) necessary for activation of the inflammasome by a second potassium-depleting signal (signal 2). Here we show that TLR3 binding to dsRNA promotes post-translational inflammasome activation through intermediate and late TRIF/RIPK1/FADD-dependent pathways. Both pathways require the scaffolding but not the catalytic function of caspase-8 or RIPK1. Only the late pathway requires kinase competent RIPK3 and MLKL function. Mechanistically, FADD/caspase-8 scaffolding function provides a post-translational signal 1 in the intermediate pathway, whereas in the late pathway it helps the oligomerization of RIPK3, which together with MLKL provides both signal 1 and 2 for inflammasome assembly. Cytoplasmic dsRNA activates NLRP3 independent of TRIF, RIPK1, RIPK3 or mitochondrial DRP1, but requires FADD/caspase-8 in wildtype macrophages to remove RIPK3 inhibition. Our study provides a comprehensive analysis of pathways that lead to NLRP3 inflammasome activation in response to dsRNA.

## Abstract (web summary)

Inflammasome activation requires a complex and incompletely understood network of signalling events. Here the authors characterize step-by-step contributions of TLR3, caspase-8, RIPK3 and MLKL to the activation of NLRP3 inflammasome in response to double-stranded RNA.

## Graphical abstract

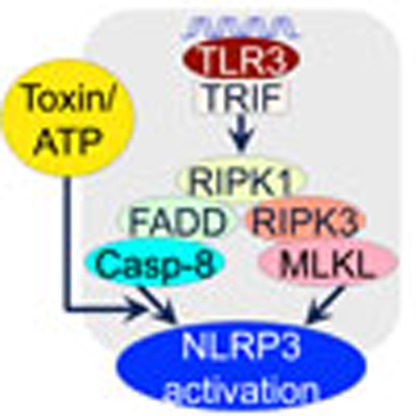

Toll-like receptors (TLRs) are pattern recognition receptors that sense a broad range of microbial ligands leading to NF-κB and IRF3 activation and expression of genes involved in inflammation and other immune responses^1^,^2^. TLR activation also leads to cell death and inflammasome activation^3^,^4^. TLR signalling involves homo- or hetero-dimerization of TLRs to bring their TIR domains in close proximity to each other, allowing the recruitment of specific TIR domain-containing adaptor molecules such as MyD88, TRIF, TIRAP (also called Mal) and TRAM^5^. All TLRs associate directly or indirectly with MyD88 except for TLR3, which associates with TRIF^5^,^6^,^7^. TLR4 is the only TLR that can associate with both TRIF and MyD88 (refs 5, 6, 7).

On binding of TLR3 to double-stranded RNA (dsRNA), it recruits TRIF through TIR–TIR homotypic domain interactions resulting in TRIF oligomerization^5^. Several outcomes follow this interaction in macrophages, depending on the composition of the complexes that are nucleated by oligomerized TRIF. Formation of a TRIF–TRAF6 complex results in TAK1 activation with subsequent activation of mitogen-activated protein kinases and NF-κB; whereas, formation of TRIF–TRAF3 activates TBK1 and IKKε, and subsequent activation of type I interferons^1^. A third complex can also form by direct interaction of TRIF with either RIPK1 or RIPK3 through their respective RHIM domains^1^,^8^,^9^,^10^. In cultured murine macrophages, low levels of cellular FLICE-like inhibitory protein results in a TRIF–RIPK1 complex, which recruits FADD and caspase-8 leading to induction of apoptosis^8^,^9^. When caspase-8 activity is compromised, this complex recruits RIPK3 to drive MLKL-dependent necroptosis^8^,^9^,^10^,^11^,^12^. In other cultured cells such as mouse embryonic fibroblasts, TRIF may directly recruit RIPK3 to initiate necroptosis independent of RIPK1 (refs 8, 10).

NLRP3 is a member of the NLR family of cytoplasmic pattern recognition receptors^13^. NLRP3 directs the assembly of an inflammasome complex with ASC and procaspase-1 after receiving a priming signal (or signal 1) from TLRs and a second signal (or signal 2) from purinergic receptors or pore-forming toxins^14^,^15^,^16^. Our recent studies showed that acute TLR2 stimulation triggers a rapid signalling pathway dependent on MyD88, IRAK1 and IRAK4, that leads to post-translational priming of NLRP3 (ref. 17). Interestingly, a second, slightly delayed TLR3- and TRIF-dependent pathway, can also lead to post-translational priming of NLRP3 (ref. 17), but the particular signalling steps involved in this novel pathway remain unknown.

Here we dissect the signal transduction pathways involved in the post-translational priming of NLRP3 by TLR3 signalling in response to extracellular dsRNA in macrophages. We show that TLR3 stimulation can activate two pathways that promote NLRP3 activation, intermediate and late, depending on the nature of the signalling molecules recruited downstream of the TRIF–RIPK1 complex. Caspase-8 scaffolding function, but not its catalytic activity, is crucial in both pathways, whereas RIPK3/MLKL activity is only required for the late pathway. We further show that cytoplasmic dsRNA can also promote NLRP3 activation through a distinct pathway that requires FADD/caspase-8 in wildtype (WT) macrophages, but not in RIPK3-deficient macrophages. Our results provide a comprehensive analysis of the NLRP3 inflammasome pathways activated by dsRNA and provide the first example of signalling by the FADD–caspase-8 complex independent of its catalytic activity.

## Results

### NLRP3 priming by TLR3 requires TRIF/RIPK1/FADD/caspase-8

**Figure 1:**
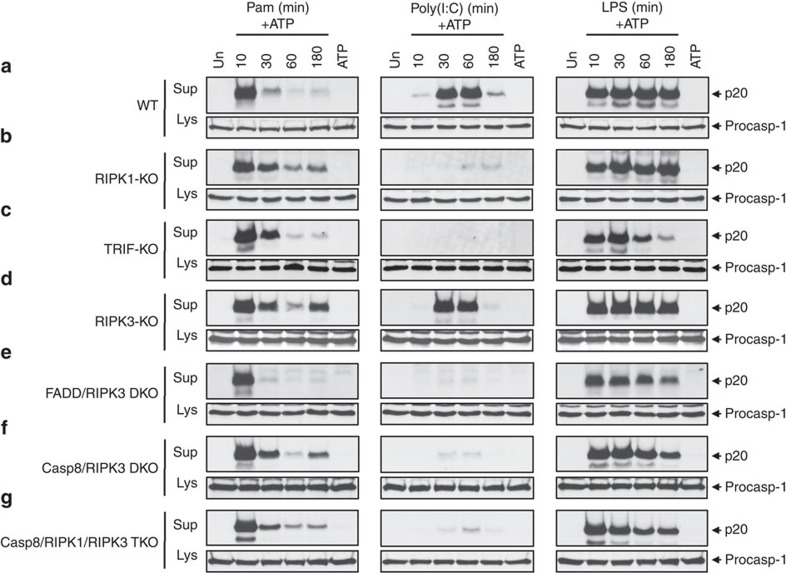
RIPK1, FADD and caspase-8 are required for activation of the NLRP3 inflammasome by dsRNA. Immunoblots of caspase-1 in the culture supernatants (Sup) or cell lysates (Lys) of C57BL/6 mouse macrophages derived from WT (**a**) RIPK1-KO (**b**) TRIF-KO (**c**) RIPK3-KO (**d**) FADD/RIPK3-DKO (**e**) caspase-8/RIPK3-DKO (**f**) Caspase-8/RIPK1/RIPK3-TKO (**g**) mice, treated with Pam3CSK4 (Pam), poly(I:C) or LPS for the indicated times (min) followed by stimulation with ATP for an additional 45 min. Results are representative of at least three independent experiments.

Our recent studies showed that stimulation of TLR3 with the synthetic dsRNA poly(I:C) primes NLRP3 activation through a post-translational, TRIF-dependent pathway designated ‘intermediate pathway’^17^. However, stimulation of TLR2 with Pam3CSK4 primes NLRP3 activation through a post-translational, MyD88–IRAK4–IRAK1-dependent pathway designated ‘early or rapid pathway’^17^. Consistent with these findings, ATP-activated caspase-1 in WT macrophages when added 30–60 min after TLR3 stimulation with poly(I:C) (Fig. 1a, middle panels). Very little activation occurred when ATP was added 10 or 180 min after poly(I:C) stimulation, indicating that maximum inflammasome activation occurred within an intermediate time window of 30–60 min following TLR3–TRIF activation (Supplementary Fig. 1). A more rapid activation of caspase-1 occurred when ATP was added 10 min after stimulation of the TLR2–MyD88 pathway with Pam3CSK4 but declined at 30 min or later (Fig. 1a, left panels). As expected, robust caspase-1 activation occurred when ATP was added at all treatment times following TLR4 stimulation with lipopolysaccharide (LPS), which activates both the MyD88 and TRIF pathways (Fig. 1a, right panels).

To further investigate the TLR3 signalling component(s) contributing to this activation pattern, we performed similar time course studies in macrophages deficient in known signalling molecules downstream of TRIF (Fig. 1b–g). RIPK1 and RIPK3 are involved in necroptosis signalling by TLR3 in macrophages^8^,^18^. Similar to the TRIF-knockout (TRIF-KO) macrophages, stimulation of RIPK1-KO macrophages with poly(I:C) plus ATP failed to induce the pattern of caspase-1 activation observed in WT macrophages, although these cells had a normal response to Pam3CSK4 plus ATP or LPS plus ATP (Fig. 1b). However, unlike the TRIF-KO macrophages, there was a weak caspase-1 activation in the RIPK1-KO at the 60 and 180 min time points and no decline in caspase-1 activation at the same time points in response to LPS plus ATP treatment (Fig. 1c). This could be explained by the recent findings that TRIF can recruit RIPK3 directly in the absence of RIPK1 when TLR3 or TLR4 are stimulated resulting in activation of a TRIF–RIPK3–MLKL pathway^10^, which is likely responsible for this late activation of caspase-1. In contrast to RIPK1 or TRIF-KO macrophages, RIPK3-KO macrophages showed normal caspase-1 activation in response to the inducers tested (Fig. 1d).

Considering that RIPK1 is an adaptor protein that recruits FADD and caspase-8 to activate apoptosis, we examined whether FADD or caspase-8 deficiency impacts TLR3-mediated NLRP3 activation. Given that FADD or caspase-8 deficiency is embryonic lethal^19^,^20^,^21^ and unleashes RIPK3-mediated necroptosis, we employed macrophages from either FADD/RIPK3 or caspase-8/RIPK3 double knockout (DKO) mice^22^,^23^,^24^. Notably, like TRIF-KO macrophages, FADD/RIPK3-DKO and caspase-8/RIPK3-DKO macrophages failed to activate caspase-1 in response to poly(I:C) plus ATP (Fig. 1e,f, middle panels) even though both lines showed caspase-1 activation in response to Pam3CSK4 plus ATP or LPS plus ATP, although there was a time-dependent loss of sensitivity to LPS plus ATP (Fig. 1e,f, left and right panels). Similar results were obtained with caspase-8/RIPK1/RIPK3 triple knockout (TKO) macrophages^10^,^11^,^12^ (Fig. 1g). Collectively, these results indicate that post-translational priming of the NLRP3 inflammasome by extracellular dsRNA through the intermediate pathway requires RIPK1, FADD and caspase-8.

### dsRNA and signal 2 induce NLRP3-mediated pyroptosis

**Figure 2:**
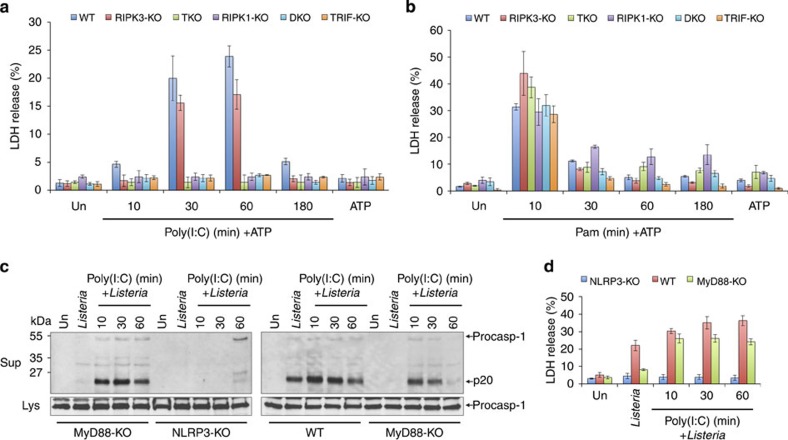
dsRNA priming promotes NLRP3-induced pyroptosis. LDH release in the culture supernatants of macrophages from WT and the indicated mouse knockout strains, treated with poly(I:C) (**a**) or Pam3CSK4 (Pam) (**b**) for the indicated times (min) followed by stimulation with ATP for an additional 45 min. DKO, caspase-8/RIPK3-DKO; TKO, caspase-8/RIPK1/RIPK3-TKO. Results are representative of at least three independent experiments. Error bars represent s.d. (**c**) Immunoblots of caspase-1 in the culture supernatants (Sup) or cell lysates (Lys) of mouse macrophages derived from WT, MyD88-KO or NLRP3-KO mice treated with poly(I:C) for the indicated times (min) followed by infection with *Listeria* (multiplicity of infection (MOI): 100) for an additional 45 min. Caspase-1 activation in macrophages infected with *Listeria* alone without prior priming with poly(I:C) is shown in lanes 2 and 7 of the left and right panels. (**d**) LDH release in the culture supernatants of macrophages derived from WT, MyD88-KO or NLRP3-KO mice treated with poly(I:C) for the indicated times (min) followed by infection with *Listeria* (MOI: 100) for an additional 45 min. LDH release in macrophages infected with *Listeria* alone without prior priming with poly(I:C) is shown in the second columns from left. Error bars represent s.d.

Activation of caspase-1 by the inflammasomes can lead to pyroptotic cell death in macrophages^25^,^26^, which can be measured by assaying the activity of released lactate dehydrogenase (LDH) in the culture medium. Consistent with the caspase-1 activation patterns described above (Fig. 1), activation of the intermediate pathway with poly(I:C) and ATP-induced LDH release from WT and RIPK3-KO, but failed to induce significant release of LDH from TRIF-KO, RIPK1-KO, caspase-8/RIPK3-DKO or caspase-8/RIPK1/RIPK3-TKO macrophages (Fig. 2a). In contrast, normal LDH release was observed in WT and all of these knockout macrophages in response to Pam3CSK4 and ATP (Fig. 2b). These results indicate that activation of the inflammasome by extracellular dsRNA and ATP can lead to TRIF/RIPK1/caspase-8-dependent pyroptotic cell death.

Extracellular dsRNA is normally produced by virally infected cells especially when they are infected with dsRNA viruses like rotaviruses. Rotaviruses infect the gastrointestinal tract and this can frequently coincide with co-infection with gram-positive bacteria such as *Listeria monocytogenes* or other NLRP3-activating toxin-producing bacteria^27^,^28^,^29^. *Listeria* is unable to activate NLRP3 through the intermediate pathway, but can activate it through the early MyD88-dependent pathway by simultaneously stimulating host TLR2–MyD88 signalling with its lipoprotein while disrupting host cell membrane by its pore-forming listeriolysin O (refs 17, 30). However, in the presence of viral dsRNA it might activate NLRP3 via the intermediate pathway. To test this possibility we infected WT, NLRP3-KO and MyD88-KO macrophages with *Listeria* with or without prior priming with poly(I:C). *Listeria* was not able to activate caspase-1 in MyD88-KO or NLRP3-KO macrophages, but was able to activate it in MyD88-KO macrophages primed with poly(I:C) (Fig. 2c). However, Listeria activated caspase-1 in WT macrophages both with or without prior priming with poly(I:C), although there was notably enhanced activation in the presence of poly(I:C) compared with in its absence (Fig. 2c, right panels). Activation of caspase-1 by *Listeria* was associated with induction of pyroptosis as determined by LDH release (Fig. 2d). These results suggest that gram-positive bacteria, which possess pore-forming toxins that can act as a signal 2 for NLRP3 activation, are able to activate NLRP3 through the intermediate pathway when this pathway is stimulated by viral dsRNA, which acts as a signal 1. This scenario can likely happen in cases of co-infection with NLRP3-activating toxin-producing bacteria such as *L. monocytogenes* or *Staphylococcus aureus* and dsRNA viruses such as rotavirus^27^,^28^,^29^.

### NLRP3 priming by TLR3 requires caspase-8 scaffolding function

**Figure 3:**
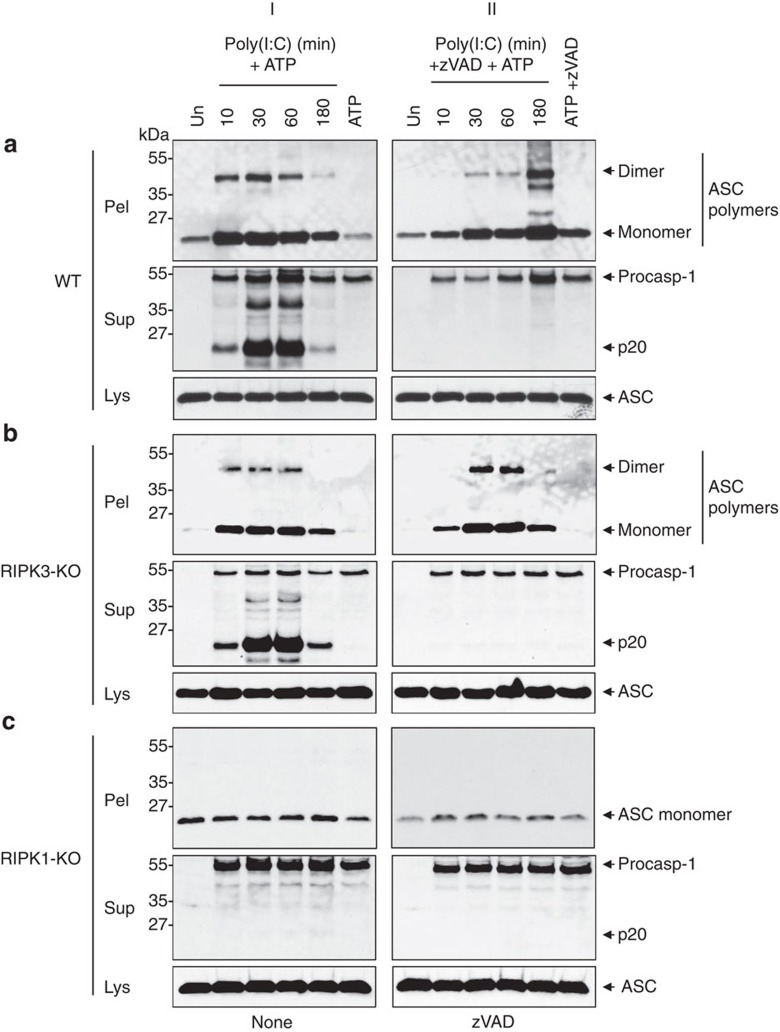
Caspase-8 enzymatic activity is not required for dsRNA-induced ASC polymerization. (**a**–**c**) (upper panels) Immunoblots of disuccinimidyl suberate (DSS) cross-linked ASC in the NP40-insoluble pellets (Pel) of WT (**a**), RIPK3-KO (**b**) and RIPK1-KO (**c**) macrophages after stimulation with poly(I:C) (I and II) for the indicated times (min) in the absence (I) or presence (II) of zVAD followed by stimulation with ATP for an additional 45 min as indicated. Immunoblots of caspase-1 in the culture supernatants (Sup) of the corresponding samples are shown underneath the ASC panels. Immunoblots of total ASC in the cell lysates (Lys) of all samples is shown at the bottom of (**a**–**c**) panels. The ASC aggregates present in the NP40-insoluble pellets are labelled ASC polymers. These fractionate as monomeric and dimeric ASC species following cross-linking with DSS, solubilization in SDS sample buffer and subsequent fractionation on SDS–PAGE. Results are representative of at least three independent experiments.

The role of caspase-8 in NLRP3 activation remains incompletely resolved. Studies in caspase-8 conditional knockout dendritic cells (caspase-8-cKO DCs) implicated caspase-8 as an inhibitor of RIPK3-mediated NLRP3 activation^31^, but these observations are clouded by the fact that caspase-8-compromise unleashes necroptosis^19^,^20^,^21^. In contrast, studies with caspase-8/RIPK3-DKO macrophages and mice reveal a contribution of caspase-8 catalytic activity in NLRP3 activation that is independent of RIPK3 (ref. 32). To address the exact role of caspase-8 in NLRP3 activation we asked whether caspase-8 catalytic activity is required for the intermediate pathway of NLRP3 activation by extracellular dsRNA. We stimulated macrophages with poly(I:C) and ATP in the presence or absence of the pan-caspase inhibitor Benzyloxycarbonyl-Val-Ala-Asp (OMe) fluoromethylketone (zVAD). Because zVAD can inhibit caspase-1 and caspase-8 activation, we used the ASC polymerization assay^25^,^33^,^34^ as an indicator of NLRP3 activation. Stimulation with poly(I:C) and ATP both in the presence or absence of zVAD induced high amounts of polymerized ASC in the pellet fractions of WT and RIPK3-KO, but not RIPK1-KO macrophages (Fig. 3a–c), indicating that inhibition of caspase-8 activity does not interfere with the intermediate pathway of NLRP3 activation. The requirement for caspase-8 (Fig. 1f,g), but not its enzymatic activity (Fig. 3), suggests that caspase-8 scaffolding function regulates NLRP3 activation in the intermediate pathway.

Intriguingly, stimulation with poly(I:C) and ATP in the presence of zVAD induced high amounts of polymerized ASC in the pellet fraction of WT (Fig. 3aII, fifth lane) but not RIPK3-KO (Fig. 3bII, fifth lane) macrophages at the 180 min time point. This suggests that caspase-8 inhibition by zVAD leads to induction of a TRIF–RIPK1–RIPK3-dependent late pathway (180 min) of NLRP3 activation in response to TLR3 signalling. The activation of this late pathway in WT macrophages caused a reduction in the amount of polymerized ASC at the 30–60 min time points (intermediate pathway; Fig. 3aII, third and fourth lanes) compared with the same time points in the absence of zVAD (Fig. 3aI, third and fourth lanes). However, a similar reduction was not observed in the RIPK3-KO macrophages (Fig. 3bI and Fig. 3bII, third and fourth lanes), indicating that recruitment of RIPK3 to the TRIF–RIPK1 complex might interfere with the function or reduce the formation of the TRIF–RIPK1–FADD–caspase-8 complex, which is required for priming of NLRP3 by the intermediate pathway.

The pattern of polymerized ASC in response to stimulation with Pam3CSK4 and ATP followed a similar time course in WT, RIPK3 and RIPK1 macrophages, and there was very little difference in the presence or absence of zVAD (Supplementary Fig. 2a–c, panels I and II), indicating that caspase-8 does not play a role in this pathway.

### dsRNA and zVAD activate NLRP3 without signal 2

**Figure 4:**
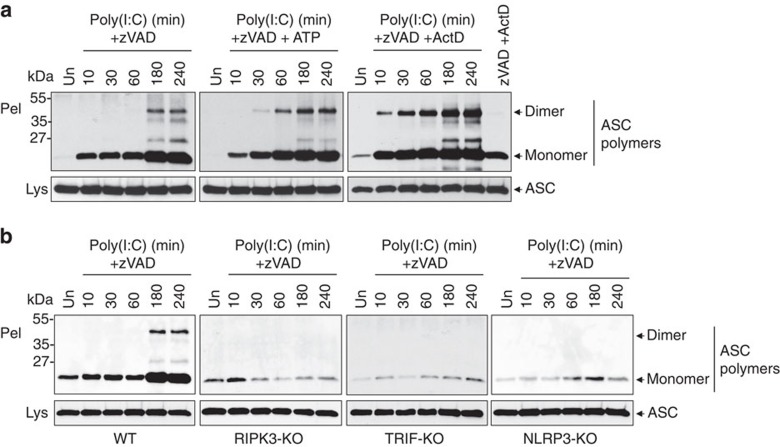
dsRNA and zVAD induce transcription- and signal 2-independent inflammasome activation. (**a**) Immunoblots of disuccinimidyl suberate (DSS) cross-linked ASC in the NP40-insoluble pellets of WT macrophages after stimulation with poly(I:C) for the indicated times (min) in the presence of zVAD (left panel), zVAD followed by stimulation with ATP for an additional 45 min (middle panel) or zVAD plus actinomycin D (ActD, right panel) as indicated. (**b**) Immunoblots of DSS cross-linked ASC in the NP40-insoluble pellets of WT (first panel), RIPK3-KO (second panel), TRIF-KO (third panel) or NLRP3-KO (fourth panel) macrophages after stimulation with poly(I:C) for the indicated times (min) in the presence of zVAD as indicated. Immunoblots of total ASC in the cell lysates (Lys) of all samples is shown at the bottom of **a**, **b** panels. Results are representative of at least three independent experiments.

NLRP3 activation requires two signals; one is derived from TLRs (signal 1) and a second signal (signal 2) is derived from purinergic receptors or pore-forming toxins^14^,^15^. To examine whether the observed poly(I:C)–zVAD-activated late pathway of inflammasome activation requires an exogenous signal 2 from P2X7 receptor, we stimulated WT macrophages with poly(I:C) and zVAD followed by treatment with or without ATP. Time course analysis of ASC polymerization showed that stimulation with poly(I:C) and zVAD alone was able to induce robust ASC polymerization comparable in magnitude to that observed when cells were further stimulated with ATP (Fig. 4a, left and middle panels). Poly(I:C) and zVAD stimulation without ATP treatment did not induce ASC polymerization in RIPK3-KO, TRIF-KO or NLRP3-KO macrophages (Fig. 4b, second to fourth panels from left ), providing additional support for the critical role of RIPK3, TRIF and NLRP3 in the late pathway of inflammasome activation.

As the late pathway of ASC polymerization requires 3–4 h of poly(I:C) stimulation, we asked whether this pathway is dependent on transcriptional upregulation of NLRP3 or other inflammasome components. Treatment of cells with actinomycin D, a potent transcription inhibitor, did not reduce the amount of polymerized ASC in response to stimulation with poly(I:C) and zVAD (Fig. 4a, right panel). On the contrary, actinomycin D treatment increased the amount of polymerized ASC and accelerated its formation kinetics. Taken together, our results show that TLR3 stimulation in the presence of inactive caspase-8 can lead to a TRIF–RIPK1–RIPK3-dependent late pathway of NLRP3 inflammasome priming and activation that is independent of exogenous signal 2 or new gene synthesis.

### RIPK3 kinase activity is required in the late pathway

**Figure 5:**
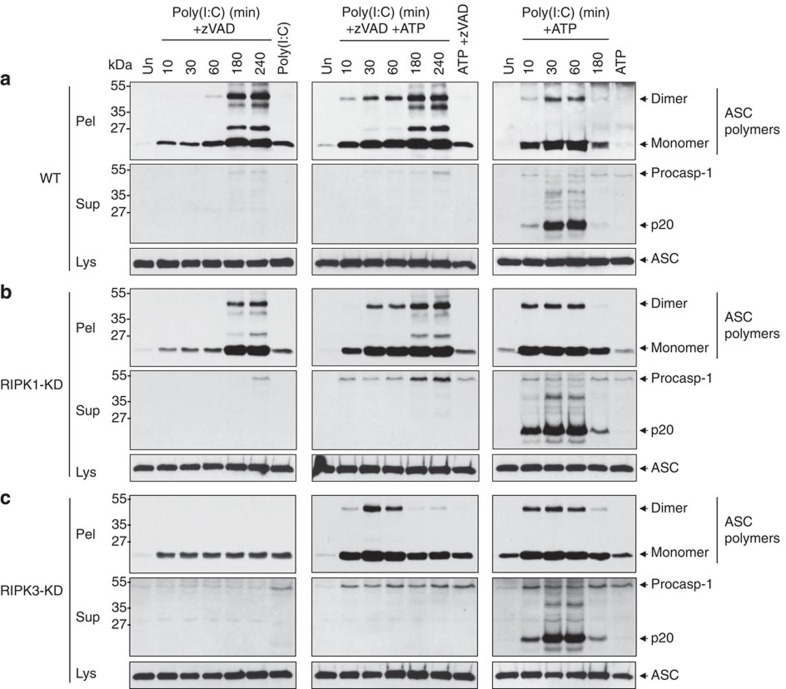
Kinase activity of RIPK3 but not of RIPK1 is required in the late pathway. (**a**–**c**) Immunoblots of disuccinimidyl suberate cross-linked ASC in the NP40-insoluble pellets of WT, kinase-dead RIPK1 (RIPK1-KD) or RIPK3 (RIPK3-KD) macrophages after stimulation with poly(I:C) for the indicated times (min) in the presence of zVAD (left panel) or zVAD followed by stimulation with ATP for 45 min (middle panel), or in the absence of zVAD followed by stimulation with ATP for an additional 45 min (right panel) as indicated. Immunoblots of caspase-1 in the culture supernatants (Sup) of the corresponding samples are shown underneath the ASC panels. Immunoblots of total ASC in the cell lysates (Lys) of all samples is shown at the bottom of **a**–**c** panels. Results are representative of at least three independent experiments.

To examine whether the kinase activity of RIPK1 or RIPK3 is important for NLRP3 activation, we performed time course studies of NLRP3 activation in response to stimulation with Pam3CSK4, poly(I:C) or LPS in knock-in macrophages expressing kinase-dead RIPK1 (RIPK1-KD) or RIPK3 (RIPK3-KD)^11^. Both RIPK1-KD and RIPK3-KD macrophages had normal caspase-1 activation similar to WT macrophages in response to Pam3CSK4, poly(I:C) or LPS plus ATP stimulation (Supplementary Fig. 3a–c), indicating that the kinase activity of RIPK1 or RIPK3 is not required for the early or intermediate pathway of NLRP3 priming. Similarly, RIPK1-KD macrophages had normal ASC polymerization similar to WT macrophages in response to poly(I:C) plus zVAD (Fig. 5a,b, left panels), indicating that the kinase activity of RIPK1 is also not required for priming or activation of the NLRP3 inflammasome by the late pathway. However, although RIPK3-KD macrophages had normal ASC polymerization during the intermediate pathway (Fig. 5c, middle and right panels), they showed no ASC polymerization during the late pathway in response to poly(I:C) plus zVAD (Fig. 5b, left panels), indicating that the kinase activity of RIPK3 is important. Consistent with this, treatment of WT macrophages with the RIPK3-specific kinase inhibitor GSK’872 (refs 8, 35) completely inhibited inflammasome activation by the RIPK3-dependent late pathway, but did not inhibit inflammasome activation by the RIPK3-independent early or intermediate pathways (Supplementary Fig. 4). Collectively, these results show that RIPK3 and its kinase activity are not required for priming of NLRP3 by the intermediate pathway, but both RIPK3 and its kinase activity are needed for priming and activation of NLRP3 by the late pathway. In addition, although RIPK1 is required as an adaptor for both the intermediate and late pathways of inflammasome activation, its kinase activity is dispensable.

### NLRP3 activation by dsRNA and zVAD requires MLKL

**Figure 6:**
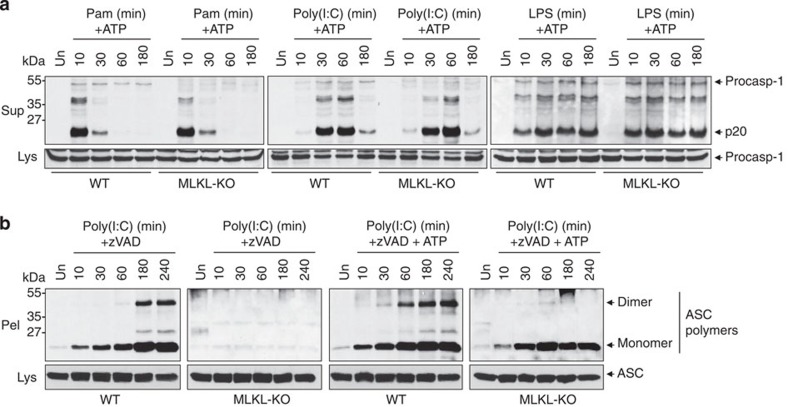
MLKL activity is critical in the late pathway. (**a**) Immunoblots of caspase-1 in the culture supernatants (Sup) or cell lysates (Lys) of mouse macrophages derived from MLKL knockout (MLKL-KO) mice treated with Pam3CSK4 (Pam), poly(I:C) or LPS for the indicated times (min) followed by stimulation with ATP for 45 min. (**b**) Immunoblots of disuccinimidyl suberate cross-linked ASC in the NP40-insoluble pellets of WT and MLKL-KO macrophages after stimulation with poly(I:C) for the indicated times (min) in the presence of zVAD (first and second panels) or zVAD followed by stimulation with ATP for an additional 45 min (third and fourth panels) as indicated. Immunoblots of total ASC in the cell lysates (Lys) of all samples is shown at the bottom. Results are representative of at least three independent experiments.

MLKL is an important substrate for RIPK3 kinase activity, and its phosphorylation by RIPK3 leads to induction of necroptosis^36^,^37^. Notably, MLKL-KO macrophages like RIPK3-KO macrophages are resistant to induction of necroptotic cell death by poly(I:C) plus zVAD (Supplementary Fig. 4c), but they exhibit normal caspase-1 activation similar to that observed in WT macrophages in response to Pam3CSK4, poly(I:C) or LPS plus ATP (Fig. 6a). As the late pathway of inflammasome activation requires the kinase activity of RIPK3, and as MLKL activation has been shown to trigger formation of plasma membrane pores^38^, we asked whether MLKL could function as a signalling molecule that facilitates NLRP3 activation downstream of RIPK3 by virtue of its pore-forming activity. Time course studies of ASC polymerization in MLKL-KO macrophages in response to poly(I:C) in the presence of zVAD with or without ATP, showed an intact intermediate pathway but a defective late pathway of inflammasome activation (Fig. 6b). Similar studies in FADD/MLKL-DKO macrophages showed that these cells have both defective intermediate and late pathways (Supplementary Fig. 5). These results indicate that intact necroptotic signalling controls activation of the late pathway and further demonstrate that FADD/caspase-8 signalling controls the intermediate pathway. The inability of ATP to stimulate ASC polymerization in the late pathway in MLKL-KO or FADD/MLKL-DKO macrophages suggests that simultaneous signalling by RIPK3 and MLKL is required to provide both signal 1 and signal 2 to activate NLRP3. Together, these results indicate that MLKL is not critical for inflammasome activation in the early or intermediate pathways, but is particularly required during the late TRIF–RIPK1–RIPK3-dependent pathway.

### RIPK3 oligomerization by dsRNA and zVAD requires caspase-8

**Figure 7:**
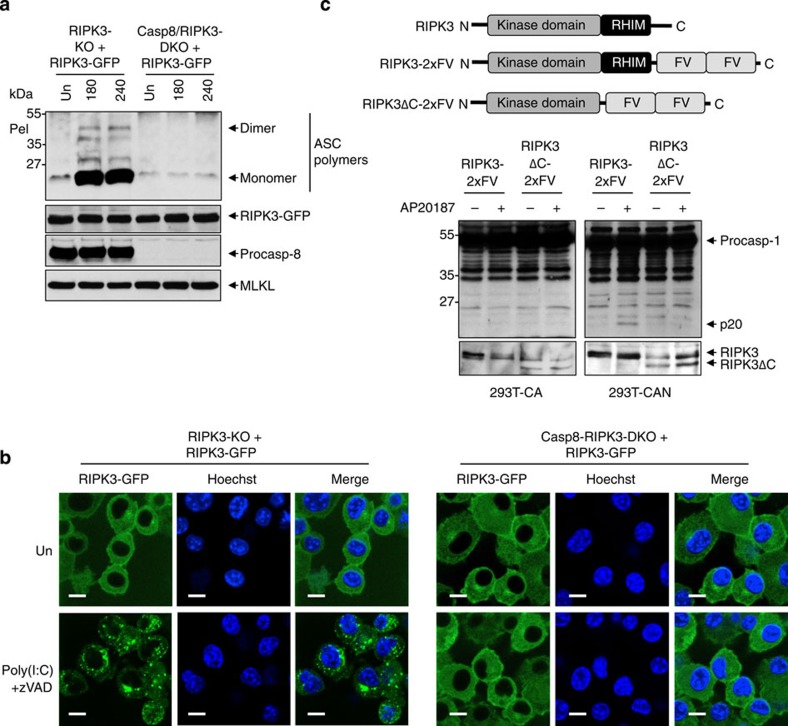
Caspase-8 scaffolding function is required for inflammasome activation and RIPK3 aggregation. (**a**) Immunoblot of disuccinimidyl suberate cross-linked ASC in the NP40-insoluble pellets (upper panel) of stable RIPK3-GFP-reconstituted RIPK3-KO (RIPK3-KO+RIPK3-GFP) or caspase-8-RIPK3-DKO (Casp8/RIPK3-DKO+RIPK3-GFP) macrophages after stimulation with poly(I:C) for the indicated times (min) in the presence of zVAD. The lower panels show immunoblots of RIPK3-GFP, caspase-8 and MLKL in the lysates of the same samples. (**b**) Confocal images of unstimulated (Un, upper panels) or poly(I:C) plus zVAD-stimulated (180 min; lower panels) stable RIPK3-GFP-reconstituted RIPK3-KO (RIPK3-KO+RIPK3-GFP) or caspase-8-RIPK3-DKO (Casp8/RIPK3-DKO+RIPK3-GFP) macrophages. Scale bar, 10 μm. (**c**) RIPK3 or RIPK3-ΔRHIM fused to two copies of FKBP^F36V^ (RIPK3-2xFV or RIPK3-ΔRHIM-2xFV, respectively; upper diagram), were ectopically expressed in 293T-CAN cell line, which is stably reconstituted with the human NLRP3 inflammasome components procaspase-1, ASC and NLRP3, or 293T-CA cell line stably reconstituted with only procaspase-1 and ASC. Caspase-1 immunoblots of cell lysates (lower panels) show caspase-1 p20 band only in RIPK3-2xFV-transfected 293T-CAN, but not in 293T-CA cells after stimulation with the oligomerization drug AP20187. Results are representative of at least three independent experiments.

To further investigate the role of caspase-8 in the late pathway, we stably reconstituted immortalized RIPK3-KO and caspase-8/RIPK3-DKO macrophages with WT green fluorescent protein (GFP)-tagged RIPK3. Notably, stable expression of RIPK3-GFP in RIPK3-KO, but not in caspase-8/RIPK3-DKO macrophages sensitized them to poly(I:C) plus zVAD as evidenced by induction of ASC polymerization (Fig. 7a). Intriguingly, confocal imaging of these macrophage lines after poly(I:C) plus zVAD treatment for 180 min showed RIPK3 oligomerization (aggregation) only in the RIPK3-GFP-reconstituted RIPK3-KO but not in the caspase-8/RIPK3-DKO macrophages (Fig. 7b). Furthermore, the RIPK3 inhibitor GSK’872 triggered a similar response in the reconstituted RIPK3-KO but not the caspase-8/RIPK3-DKO macrophages (Supplementary Fig. 6). No aggregation of RIPK3 was observed when poly(I:C) or zVAD were added to the cells alone, but GSK’872 was able to induce RIPK3 aggregation in RIPK3-KO–RIPK3-GFP cells when added alone (Supplementary Fig. 6). Stimulation of RIPK3-KO–RIPK3-GFP cells with poly(I:C) plus zVAD for 45 or 180 min followed with or without ATP treatment did not change the pattern of RIPK3-GFP aggregation (Supplementary Fig. 7). There were only fewer cells that formed RIPK3-GFP aggregates at 45 min compared with 180 min after stimulation, indicating that RIPK3 recruitment to the TRIF–RIPK1 complex starts as early as 45 min after stimulation with poly(I:C) plus zVAD. Consistent with the lack of RIPK3 involvement in the intermediate pathway, stimulation of RIPK3-KO-RIPK3-GFP cells with poly(I:C) for 45 min followed by ATP treatment in the absence of zVAD did not result in any RIPK3-GFP aggregation (Supplementary Fig. 7). Combined, these results indicate that caspase-8 is physically required for oligomerization of RIPK3 downstream of TLR3 and TRIF, as well as by binding of GSK’872 to RIPK3.

To provide additional evidence that direct oligomerization of RIPK3 can induce NLRP3 activation we ectopically expressed a chimeric protein composed of RIPK3 or RIPK3-ΔRHIM fused to two copies of FKBP^F36V^ (RIPK3-2xFV or RIPK3-ΔRHIM-2xFV, respectively)^39^ in a 293T cell line stably reconstituted with procaspase-1, ASC and NLRP3 (293T-C1AN cells), or a 293T cell line stably reconstituted with only procaspase-1 and ASC (293T-C1A cells). Treatment of the RIPK3-2xFV cells with the homodimerization drug AP20187 led to activation of caspase-1 in 293T-C1AN cells, but not in 293T-C1A cells (Fig. 7c). There was no caspase-1 activation in the ΔRHIM expressing cells after a similar treatment (Fig. 7c). Similar results were obtained when these constructs were stably expressed in 293T-C1AN (Supplementary Fig. 8). These results indicate that direct oligomerization of RIPK3 can induce RHIM-dependent NLRP3 activation.

### NLRP3 activation by cytoplasmic dsRNA requires caspase-8

**Figure 8:**
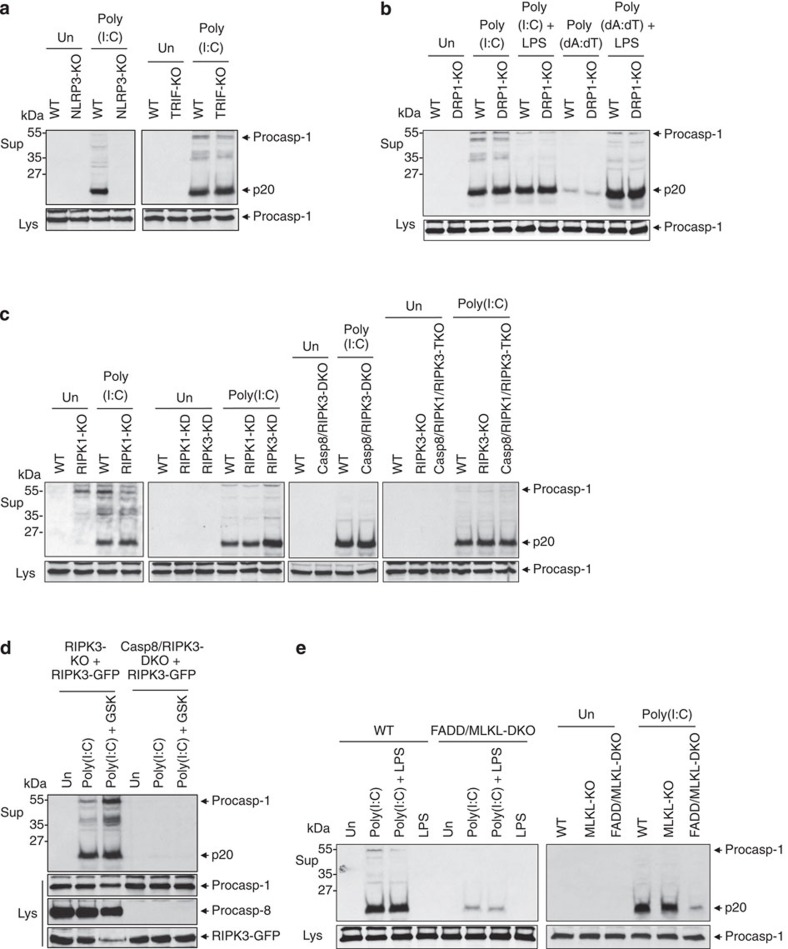
Cytoplasmic dsRNA requires FADD and caspase-8 but not DRP1 for inflammasome activation. Immunoblots of caspase-1 in the culture supernatants (Sup) or cell lysates (Lys) of WT or the indicated knockout primary macrophages (**a**–**c**,**e**) or stable RIPK3-GFP-reconstituted RIPK3-KO (RIPK3-KO+RIPK3-GFP) or caspase-8-RIPK3-DKO (Casp8/RIPK3-DKO+RIPK3-GFP) immortalized macrophages (**d**) transfected with poly(I:C) in the presence or absence of GSK’872 (GSK) for 5 h as indicated. The lower two panels in **d** show immunoblots with anti-caspase-8 or anti-RIPK3 antibodies. The decrease in RIPK3-GFP in the lysates (third lane) is due to GSK’872-induced RIPK3-GFP aggregation in the insoluble fraction. Results are representative of at least three independent experiments.

Cytoplasmic dsRNA and vesicular stomatitis virus (VSV) have been reported to induce NLRP3 activation through a RIPK1–RIPK3–DRP1-dependent and TLR3/TRIF-independent pathway^40^. Our results show that transfected poly(I:C)-induced caspase-1 activation indeed requires NLRP3 but not TRIF signalling (Fig. 8a). However, poly(I:C)-induced caspase-1 activation in DRP1-KO macrophages derived from conditional myeloid-specific DRP1-KO mice^41^ was comparable to WT macrophages (Fig. 8b). DRP1-KO macrophages had also a normal response to transfected poly(dA:dT), which activates the AIM2 inflammasome^26^ (Fig. 8b). Poly(I:C) induced similar levels of caspase-1 activation with or without prior priming with LPS (Fig. 8b, third to sixth lanes). However, poly(dA:dT) required prior LPS priming to induce efficient caspase-1 activation (Fig. 8b, seventh to tenth lanes). DRP1-KO macrophages also showed normal inflammasome activation by the early, intermediate and late pathways (Supplementary Fig. 9a–d). Similarly, deficiency in RIPK1 or RIPK3 had no effect on poly(I:C)-induced NLRP3 activation (Fig. 8c, fourth and nineteenth lanes), whereas kinase-dead RIPK3 enhanced it (tenth lane). Together, these results indicate that DRP1, RIPK1 or RIPK3 do not play a role in NLRP3 activation by cytoplasmic dsRNA.

As FADD/caspase-8 signalling plays an important role in activation of the inflammasome by extracellular dsRNA we asked whether it plays a similar role in inflammasome activation by transfected dsRNA. Caspase-8 deficiency in caspase-8/RIPK3-DKO, or caspase-8/RIPK1/RIPK3-TKO macrophages did not affect poly(I:C)-induced caspase-1 activation, which was comparable to that in WT or RIPK3-KO macrophages (Fig. 8c, fourteenth and twentieth lanes, and Supplementary Fig. 10). Surprisingly, stable expression of RIPK3-GFP blocked poly(I:C)-induced caspase-1 activation in caspase-8/RIPK3-DKO, but not in RIPK3-KO immortalized macrophages (Fig. 8d and Supplementary Fig. 11), suggesting that caspase-8 might only be required when RIPK3 is present. Consistent with this, transfected poly(I:C) failed to induce robust caspase-1 activation in FADD/MLKL-DKO macrophages, but induced comparable caspase-1 activation in MLKL-KO macrophages as in WT macrophages (Fig. 8e). Altogether, these results demonstrate that the cytoplasmic dsRNA sensor requires FADD/caspase-8 signalling in WT macrophages to relieve the inhibition of RIPK3 and facilitate NLRP3 inflammasome assembly.

### VSV-induced NLRP3 activation partially requires RIPK3

**Figure 9:**
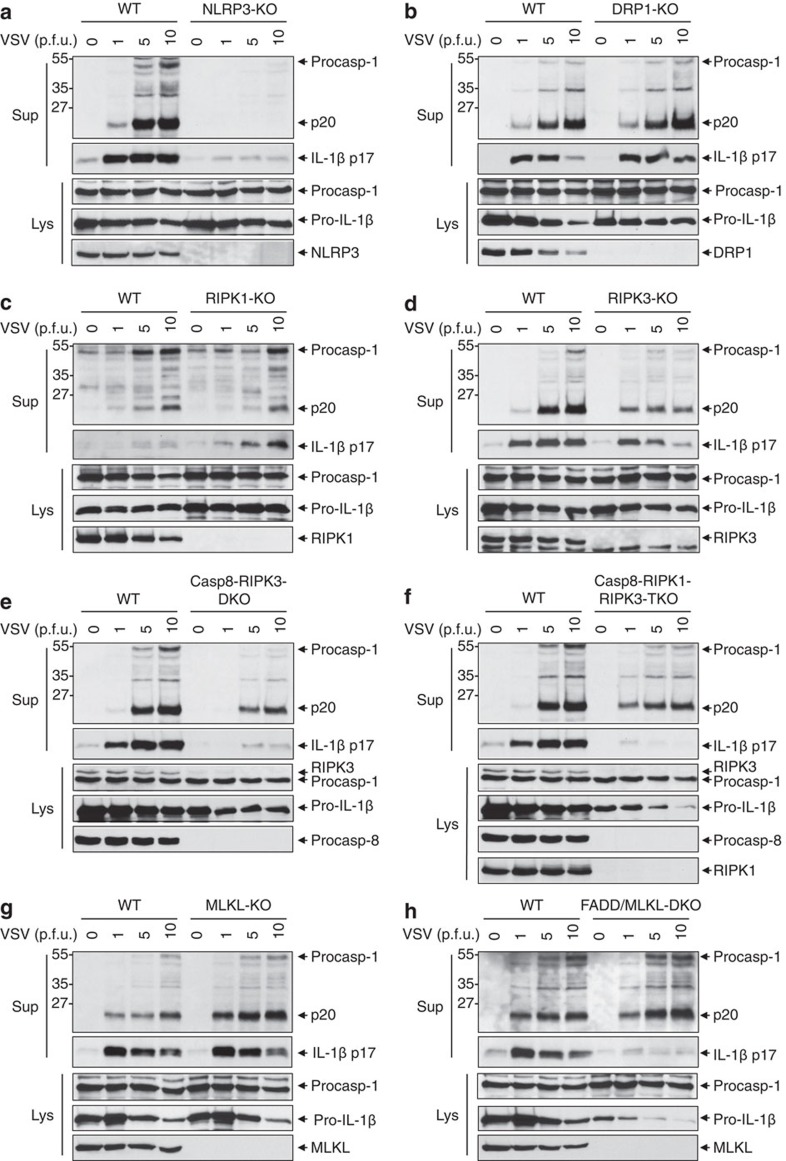
VSV-induced NLRP3 activation is partially dependent on RIPK3 but not on DRP1 or RIPK1. (**a**–**h**) Immunoblots of caspase-1 (first panels from top) and mature IL-1β p17 (second panels from top) in the culture supernatants (Sup) of macrophages derived from WT or the indicated knockout mice after infection with the indicated doses of VSV (plaque forming units (p.f.u.)) for 16 h. Immunoblots of procaspase-1, pro-IL-1β and knocked out proteins in the total cell lysates are shown underneath the supernatants blots. Results are representative of at least three independent experiments.

Infection of macrophages with single-stranded RNA viruses such as VSV activates the NLRP3 inflammasome^40^,^42^. Consistent with this, NLRP3 deficiency blocked VSV-induced caspase-1 activation (Fig. 9a). VSV has been reported to activate NLRP3 via RIPK1–RIPK3–DRP1 signalling^40^. However, our results show that deletion of DRP1 or RIPK1 have no effect on inflammasome activation by VSV infection (Fig. 9b,c). VSV was also still able to induce caspase-1 activation and interleukin (IL)-1β generation in RIPK3-KO macrophages (Fig. 9d and Supplementary Fig. 12) although the intensity of caspase-1 activation in the RIPK3-KO macrophages was partially reduced compared with WT macrophages but was not absent like in the NLRP3-KO macrophages (Fig. 9a). VSV-induced inflammasome activation was also notably reduced in caspase-8/RIPK3-DKO and caspase-8/RIPK1/RIPK3-TKO macrophages (Fig. 9e,f), but not in MLKL-KO or FADD/MLKL-DKO macrophages (Fig. 9g,h). IL-1β production from caspase-8/RIPK3-DKO, caspase-8/RIPK1/RIPK3-TKO and FADD/MLKL-DKO macrophages was much more reduced compared with that from RIPK3-KO macrophages (Fig. 9d–f,h) because FADD/caspase-8 deficiency causes reduced expression of pro-IL-1β^32^. VSV-induced inflammasome activation was comparable in WT, TRIF-KO, RIPK1-KD and RIPK3-KD macrophages and in the RIPK3–GFP-reconstituted RIPK3-KO-RIPK3-GFP and caspase-8/RIPK3-DKO-RIPK3-GFP macrophages (Supplementary Fig. 13a–c). Together, these results indicate that VSV-induced inflammasome activation is partially dependent on RIPK3 but independent of TRIF, RIPK1, MLKL, FADD and caspase-8 or the kinase activities of RIPK1 or RIPK3.

## Discussion

TLR signalling is one of the most important signalling events that regulate NLRP3 inflammasome activation^14^,^15^,^17^. However, the critical proteins downstream of TLRs that are involved in NLRP3 priming and activation and their exact role in this process have not been fully elucidated. Caspase-8 is an important component of TLR signalling. It is recruited by both TLR3 and TLR4 to induce apoptosis and regulates NF-κB activation and necroptosis^9^. Recent studies provided conflicting results on its role in NLRP3 activation^31^,^32^. Studies in caspase-8/RIPK3-DKO macrophages revealed that loss of caspase-8 inhibits NLRP3 priming and activation by both canonical and noncanonical stimuli^32^. Similar results were also reported in FADD/RIPK3-DKO macrophages^32^. In contrast, studies in caspase-8-cKO DCs showed that loss of caspase-8 facilitates LPS-induced NLRP3 activation through the RIPK3 necroptotic pathway^31^. To address these conflicting results we assessed the activity and assembly of the NLRP3 inflammasome during the early and late events of TLR signalling in cells containing or lacking caspase-8 activity. We used caspase-1 processing and ASC polymerization assays, which allow direct readout of inflammasome assembly in response to TLR signalling in the absence of new protein synthesis. We particularly avoided the widely used IL-1β secretion assay as a readout of inflammasome activation because this assay requires priming of bone marrow-derived macrophages (BMDMs) for 4–6 h to transcriptionally induce proIL-1β. This prolonged priming with LPS can desensitize both the MyD88 and TRIF pathways and can also lead to transcriptional induction of genes that may affect inflammasome assembly, which could complicate analysis of the individual contribution of MyD88, TRIF and other downstream signalling molecules to the post-translational events that regulate inflammasome assembly.

**Figure 10:**
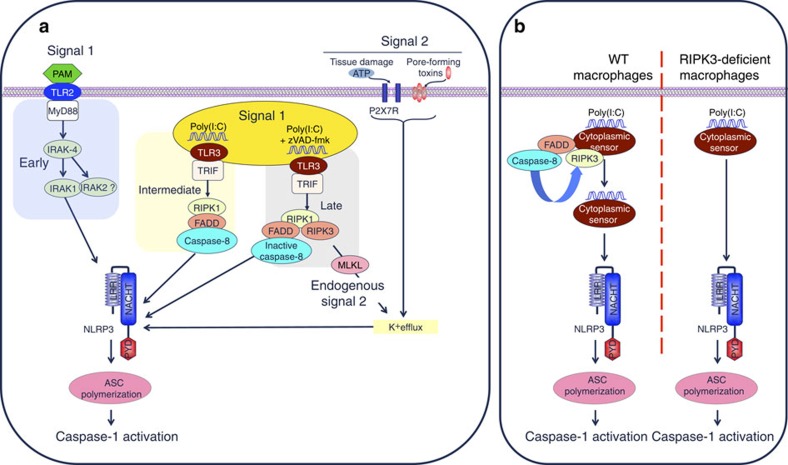
Schematic representations of the TLR and cytoplasmic dsRNA pathways that regulate NLRP3 activation. (**a**) Three pathways downstream of TLRs are involved in priming and activation of the NLRP3 inflammasome. The early MyD88-dependent pathway provides a priming signal 1 downstream of TLR2, whereas the intermediate TRIF-dependent pathway provides a priming signal 1 downstream of TLR3. Both of these pathways require exogenous signal 2 from purinergic receptors or pore-forming toxins for activation of NLRP3. Signal 2 is required to induce potassium efflux. The late TRIF-dependent pathway can provide both signal 1 and an endogenous signal 2 by recruiting RIPK3 and MLKL when caspase-8 is inhibited. (**b**) In WT macrophages, FADD/caspase-8 complex is required to remove RIPK3 inhibition of the cytoplasmic dsRNA sensor. In RIPK3-deficient macrophages such as Casp8/RIPK3-DKO macrophages, caspase-8 is not required for activation of NLRP3 by dsRNA sensor.

Our results show that signalling downstream of TLRs can regulate NLRP3 activation via three post-translational pathways depending on the adaptor molecules engaged (Fig. 10a). The early pathway is triggered by recruitment of the adaptor molecule MyD88 to TLRs, which in turn recruits IRAK4 and IRAK1 to post-translationally prime NLRP3 (ref. 17). In this early pathway, caspase-8 or its adaptor molecule FADD does not appear to play a critical role, as no defect is observed in activation of NLRP3 by the TLR2 ligand Pam3CSK4 plus ATP in FADD/caspase-8-deficient macrophages. In contrast, FADD/caspase-8 appear to be critical for an intermediate pathway of post-translational NLRP3 priming, which is controlled by the adaptor molecule TRIF downstream of TLR3 and TLR4 (ref. 5). The intermediate pathway can be clearly detected following treatment with ATP 30–60 min after poly(I:C) stimulation of TLR3. This pathway also requires RIPK1 but not RIPK3 or the enzymatic activity of caspase-8. Our assays of ASC polymerization revealed that inflammasome assembly occurs normally after stimulation of TLR3 with poly(I:C) in the presence of the potent pan-caspase inhibitor zVAD, indicating that caspase-8 plays a scaffolding role rather than a proteolytic enzyme in NLRP3 activation by extracellular dsRNA plus ATP and that its enzymatic activity is not important for NLRP3 activation under these conditions.

Physiologically, the MyD88-dependent early pathway plays a critical role in the rapid activation of the NLRP3 inflammasome by gram-positive bacteria such *L.monocytogenes* and possibly others such as *S. aureus*, which can secrete NLRP3-activating toxins and stimulate the MyD88 simultaneously^17^,^30^. However, our data suggest that the intermediate pathway might also serve to enhance inflammasome activation in conditions of co-infections with dsRNA viruses such as rotaviruses and pathogenic bacteria that produce NLRP3-activating toxin such as *L. monocytogenes*, *S. aureus* and *Clostridium difficile.* Indeed, co-infection with rotaviruses and toxin-producing bacteria are frequently observed in conditions of acute gastroenteritis^27^,^28^,^29^. The synergistic effect of dsRNA produced by rotaviruses on inflammasome activation might be responsible for the more severe clinical presentation and inflammation observed in these conditions.

Caspase-8 scaffolding function does not appear to be limited to post-translational priming of NLRP3 during the intermediate pathway. Our results indicate that caspase-8 scaffolding function controls yet another distinct late pathway of NLRP3 activation that can only be detected under conditions in which caspase-8 enzymatic activity is compromised. In this pathway ASC polymerization is observed 3–4 h following stimulation with poly(I:C) and zVAD. Importantly, although this late pathway of inflammasome activation requires a longer period of stimulation (3–4 h), it does not require new gene expression indicating that it is activated by a post-translational modification mechanism. Furthermore, this late pathway does not require exogenous signal 2 (for example, ATP), indicating that it can simultaneously provide a priming signal (signal 1) and an activation signal (signal 2) to facilitate assembly of the inflammasome.

Our analysis of the late pathway revealed that it is controlled by both RIPK1 and RIPK3 as loss of either RIPK1 or RIPK3 leads to inhibition of inflammasome assembly during this phase. As RIPK1 and RIPK3 are critical mediators of the necroptotic pathway, it is likely that the late pathway of inflammasome activation is induced by activation of the RIPK3–MLKL necroptotic pathway. Consistent with this possibility, the kinase activity of RIPK3 is critical for activation of NLRP3 by the late pathway. Like RIPK3-KO macrophages, RIPK3 kinase-dead macrophages or WT macrophages treated with the RIPK3 inhibitor GSK’872 exhibited a defect in activation of NLRP3 by the late pathway. Importantly, both RIPK3 kinase-dead and RIPK3-KO macrophages were unable to activate NLRP3 through the late pathway in the presence or absence of signal 2 (ATP), suggesting that the kinase activity of RIPK3 is critical for providing both signal 1 and signal 2 in this pathway. As the late pathway can be activated by poly(I:C) plus zVAD without the need for stimulation of the P2X7 receptor with ATP, it follows that the RIPK3 kinase activity somehow can induce potassium efflux (signal 2) through the cell membrane, an event critical for NLRP3 activation^16^,^25^,^43^. How RIPK3 can induce potassium efflux and also provide a priming signal (signal 1) in the late pathway is currently unknown, but it is likely that phosphorylation of MLKL by RIPK3 can induce potassium efflux by stimulating the pore-forming activity of MLKL^38^. Consistent with this possibility, genetic deletion of MLKL resulted in inhibition of NLRP3 inflammasome activation by the late pathway.

RIPK3-GFP expressing macrophages showed clear RIPK3 aggregation into speck-like structures after stimulation with extracellular dsRNA only when caspase-8 activity is compromised, but not in the absence of caspase-8, indicating that the physical presence of inactive caspase-8 is a critical step in the recruitment and oligomerization of RIPK3 by TLR3 signalling. The physical presence of inactive caspase-8 was also required for assembly of the inflammasome in these cells. Intriguingly, RIPK3 aggregation by the RIPK3 kinase inhibitor GSK’872 was also dependent on the physical presence of caspase-8, but caspase-8 inhibition was not required (Supplementary Fig. 6). Indeed, this inhibitor recruits caspase-8 to the inhibited RIPK3 by stimulating binding of RIPK3 to RIPK1 and FADD^35^. This certainly raises the possibility that the scaffolding function of caspase-8 together with its catalytic activity is also important for RIPK3-induced apoptosis^35^. Our observations suggest that caspase-8 physically nucleates RIPK3 oligomerization in response to the RIPK3 kinase inhibitor GSK'872, or in response to TLR3 signalling when its caspase activity is compromised.

LPS stimulation has been shown to activate the NLRP3 inflammasome in caspase-8-cKO DCs in the absence of exogenous signal 2 (ATP) in a RIPK1–RIPK3–MLKL-dependent manner^31^. Similarly LPS stimulation combined with inhibitors of apoptosis proteins (IAP) inhibition by SMAC mimetics, IAP deletion or A20 deletion can all lead to RIPK3-dependent inflammasome activation in the absence of exogenous signal 2 (refs 44, 45, 46). LPS plus IAP inhibition appears to trigger two RIPK3-dependent inflammasome activation pathways in the absence of exogenous signal 2; one is dependent on MLKL when caspase-8 is inhibited and the other is dependent on caspase-8 activity when MLKL is deleted^46^. Our findings demonstrate that dsRNA can activate NLRP3 without exogenous signal 2 only in the presence of inhibited caspase-8 through the TRIF–RIP1–RIP3-dependent late pathway. This late pathway is dependent on MLKL and might be similar to the pathway that is activated by LPS and IAP inhibitors when caspase-8 is inhibited. In addition, this pathway might also be similar to the pathway activated by LPS in caspase-8-cKO DCs, which is also dependent on MLKL^31^. As the scaffolding function of caspase-8 is required for aggregation of RIPK3 and activation of NLRP3 by poly(I:C) and zVAD, it is likely that it is also required for activation of the inflammasome by LPS and IAP inhibitors when caspase-8 is inhibited, and by LPS in caspase-8-cKO DCs. As Cre-Lox recombination does not totally eliminate caspase-8 expression in caspase-8-cKO DCs^31^,^46^, it is likely that the remaining caspase-8 has very low protease activity because of binding to cellular FLICE-like inhibitory protein, but is sufficient to provide a scaffolding function for the assembly of the RIPK1–RIPK3–FADD–caspase-8 complex.

Caspase-8 is also important for NLRP3 inflammasome activation by cytoplasmic dsRNA in WT macrophages but not in RIPK3-deficient macrophages. This suggests that RIPK3 blocks inflammasome activation at the level of the cytoplasmic dsRNA sensor (Fig. 10b). Caspase-8 activity might be required to relieve this inhibition by cleaving RIPK3 or another regulator of RIPK3 (ref. 47). Cytoplasmic dsRNA can activate the inflammasome independent of TRIF^40^,^48^,^49^, RIPK1 or RIPK3, or their kinase activities (Fig. 8). In fact, inhibition of RIPK3 kinase activity with GSK’872 or mutating the kinase domain as in the RIPK3-KD macrophages enhances cytoplasmic dsRNA-induced inflammasome activation (Fig. 8c,d). As both GSK’872 and mutation of the kinase domain of RIPK3 enhance caspase-8 activation^35^,^50^ likely by enhancing the oligomerization of RIPK3 (Fig. 7), these observations suggest that sensing of cytoplasmic dsRNA by their putative sensor leads to assembly of a FADD–caspase-8 complex that regulates RIPK3 association with, or inhibition of, this sensor. The future identification of this cytoplasmic dsRNA sensor should shed more light on how it regulates NLRP3 activation.

Our findings are in conflict with a recent report proposing that the mitochondria fission regulator DRP1 is required for NLRP3 inflammasome activation by cytoplasmic dsRNA and RNA viruses^40^. Our results show clearly that DRP1-KO macrophages have no defect in inflammasome activation in response to TLR3 stimulation, cytoplasmic dsRNA or VSV. Moreover, our findings suggest that VSV-induced NLRP3 activation is only partially dependent on RIPK3 suggesting that VSV can utilize an additional pathway to activate the inflammasome. This conflict is likely due to the use of different systems. Whereas in this study we used macrophages from conditional DRP1-KO mice, the previous study used siRNA-knockdown experiments.

In conclusion, our results provide a detailed picture of the multiple signalling pathways activated by foreign RNA to control NLRP3 activation and the central roles played by the FADD/caspase-8, RIPK3 and MLKL complexes in these pathways. Future investigation of the mechanism of NLRP3 activation by extracellular and intracellular dsRNA should shed more light on these intricate pathways.

## Methods

### Antibodies and reagents

Antibodies against caspase-1, NLRP3 and ASC were made in house and were described previously^14^,^25^,^51^. Anti-DRP1 antibody (Catalogue No. 611738) and anti-RIPK1 clone 38 (Catalogue No. 610459) were from BD biosciences. Anti-caspase-8 1G12 was from Enzo (Catalogue No. ALX-804-447-C100). Anti-MLKL clone 3H1 was from EMD Millipore (Catalogue No. MABC604). Anti-IL-1β was from GeneTex (Catalogue No. GTX74034). Anti-RIPK3 antibody (Catalogue No. R4277), ATP and actinomycin D were obtained from Sigma. Ultrapure LPS, Pam3CSK4 and poly(I:C) were obtained from InvivoGen. zVAD was obtained from ApexBio. GSK’872 was obtained from Aobious. AP20187 was obtained from Clontech. CytoTox96 LDH-release kit was from Promega. All antibodies were used at 1/1,000–1/2,000 dilutions for western blot analyses.

### Mice

C57BL/6, *Trif*^*−/−*^ and *Myd88*^*−/−*^ mice were obtained from The Jackson Laboratory (Bar Harbor, ME) and were bred at Thomas Jefferson University. *Nlrp3*^*−/−*^ (refs 16, 52, 53), *Ripk3*^*−/−*^ (ref. 54), *Casp8*^*−/−*^/*Ripk3*^*−/−*^ (refs 23, 24), *Casp8*^*−/−*^*/Ripk1*^*−/−*^*/Ripk3*^*−/−*^ (refs 10, 11, 12), *Fadd*^*−/−*^*/Ripk3*^*−/−*^, *Ripk1*^*K45A/K45A*^ (RIPK1-KD)^11^, *Ripk3*^*K51A/K51A*^ (RIPK3-KD)^11^, *Mlkl*^*−/−*^ (ref. 36), *Fadd*^*−/−*^/*Mlkl*^*−/−*^ and *Drp1*^*fl/fl*^ *LysmCre*^*+/−*^ were all on C57BL/6 background. Mouse strains were maintained in specific pathogen-free conditions and the animal protocols were carried out in accordance with the guidelines set forth by Institutional Animal Care and Use Committee. Mice of both genders (age 1–6 months) were used for harvesting bone marrow-derived macrophages.

### Cell culture and treatments

Bone marrow-derived cells were harvested from the femurs of WT (C57BL/6) and knockout mice and differentiated into BMDMs by culturing in DMEM (GIBCO) medium supplemented with 10% FBS, 10 mM HEPES pH 7.0 (Invitrogen), 100 U ml^−1^ penicillin and streptomycin (complete DMEM) and 20% L929 supernatants in 10 cm dishes at 37 °C with 5% CO_2_ for 5–6 days.

For the various treatments, BMDMs were seeded in six-well plates at a density of 1 × 10^6^ cells per well overnight. The next day BMDMs were pre-stimulated with the TLR ligands Pam3CSK4 (1 μg ml^−1)^, poly(I:C) (1 μg ml^−1^) or ultrapure LPS (500 ng ml^−1^) for various periods of times followed by stimulation with ATP (5 mM) for 45 min in OPTI-MEMI medium. In some experiments BMDMs were pre-treated with zVAD (30 μM) or actinomycin D (0.5 μg ml^−1^) before stimulation with the TLR ligands.

For transfection experiments, BMDMs were seeded in six-well plates at a density of 1 × 10^6^ cells per well overnight in DMEM medium. The next day, the culture medium was removed and replaced with 1 ml of OPTI-MEMI medium per well. Cells were primed with ultrapure LPS (500 ng ml^−1^) or left unprimed for 4 h and then transfected with poly(I:C) (2 μg ml^−1^) or poly(dA:dT) (1 μg ml^−1^) using Lipofectamine 2000 (7 μl ml^−1^) as per the manufacturer's protocol (Invitrogen).

### Generation of immortalized macrophage cell lines

Immortalized caspase-8/RIPK3-DKO and RIPK3-KO BMDMs and fetal liver-derived RIPK1-KO macrophages were generated by transformation with J2-CRE retrovirus (J2-CRE cell line was a kind gift from Dr. Howard Young, National Institutes of Health) as described before^55^ with minor modifications and grown in complete DMEM medium without L929 supernatants at 37 °C with 5% CO_2_. To generate immortalized macrophage cell lines, 1–3 × 10^6^ primary bone marrow- or fetal liver-derived cells were seeded in 60-mm dishes for 48 h in complete DMEM medium supplemented with 20% L929 supernatants. The culture medium was removed and replaced with 5 ml virus infection mix made of complete DMEM medium (2 ml), J2-CRE cell supernatant (2 ml 48 h culture supernatant), 8 μg ml^−1^ Polybrene (Sigma) and 20% L929 supernatants (1 ml). After 24 h at 37 °C, the virus infection mix was replaced with complete DMEM medium supplemented with 20% L929 supernatants and the cells were allowed to grow for 7 days. Cells were then maintained in complete DMEM medium.

### Generation of stable RIPK3-GFP cell lines

Stable RIPK3-KO and caspase-8/RIPK3-DKO immortalized macrophages expressing WT murine RIPK3-GFP were generated by retroviral transduction using a pMSCVpuro-RIPK3-GFP retroviral vector. This vector was constructed by excising the RIPK3-GFP insert from pEGFP-N1-RIPK3-GFP plasmid^56^ (a gift from Francis Chan; Addgene Plasmid #41382) with *Bgl*II-*Hpa*I restriction enzymes followed by subcloning it into the same sites of pMSCVpuro vector (Clontech).

### Caspase-1 immunoblotting

The culture supernatants and cells were collected after each stimulation and analysed for caspase-1 activation by immunoblotting as described previously^26^. Briefly, the culture supernatants were precipitated by the addition of an equal volume of methanol and 0.25 volumes of chloroform. The resulting protein pellets were resuspended in Laemmli buffer, and then fractionated by 12.5% SDS–PAGE followed by electroblotting onto nitrocellulose membranes. Blots were probed with a rabbit polyclonal antibody to mouse caspase-1. For analysis of procaspase-1 in cell pellets, total cell lysates were mixed with SDS sample buffer, fractionated on 12.5% SDS–PAGE and then immunoblotted as described above. Images have been cropped for presentation. Full size images are presented in Supplementary Figs. 14–52.

### LDH Release assay

Pyroptosis and necroptosis were quantitated by assaying the activity of LDH released into cell culture supernatants after various treatments using the CytoTox96 LDH release kit (Promega) according to the manufacturer’s protocol. The LDH activity in the culture supernatant was expressed as a percentage of total LDH in the cell lysate.

### Infection of macrophages with *Listeria*

These experiments were performed essentially as described before^55^ with minor modifications. WT *Listeria monocytogenes* (10403S) were grown to logarithmic phase in brain heart infusion medium at 37 °C with continuous shaking at 250 r.p.m. On the day of infection, a 1/10 dilution of the overnight culture was prepared in fresh brain heart infusion medium and allowed to grow at 37 °C with shaking to about *A* 600=0.5, which corresponds to ∼109 colony-forming units per ml. Bacteria were pelleted at 5,000 r.p.m. and the bacterial pellet was diluted to the desired concentration in OPTI-MEM and used to infect macrophages at multiplicity of infection of 100 following priming for different lengths of time with poly(I:C) (1 μg ml^−1^). After 45 min of infection at 37 °C, culture supernatants and cell pellets were collected and assayed as indicated.

### Viral infection of macrophages

BMDMs were seeded in six-well plates at a density of 1x10^6^ cells per well overnight. The next day, BMDMs were pre-stimulated with Pam3CSK4 (1 μg ml^−1^) for 3 h followed by infection with different doses of VSV-GFP (Indiana strain) in OPTI-MEMI medium for an additional 16 h. Active caspase-1 and IL-1β p17 were assayed in the culture supernatants of infected cells by immunoblotting as described above.

### ASC polymerization assay

ASC polymerization was assayed as described before with minor modifications^25^,^33^. After stimulation of cells in OPTI-MEMI in six-well plates, the culture supernatants were collected and used for immunoblot analyses of secreted caspase-1 p20 as described above. Cells were lysed in 0.5 ml buffer containing 20 mM Hepes-KOH, pH7.5, 150 mM KCl, 1% NP40, 0.1 mM phenylmethylsulphonyl fluoride and protease inhibitor cocktail on ice. The cell lysates were centrifuged at 6,000 r.p.m. at 4 °C for 10 min. The NP40-soluble supernatants were removed and the NP40-insloluble pellets were washed 1 × in the lysis buffer and then resuspended in 50 μl of the same buffer. The resuspended pellets were cross-linked with fresh disuccinimidyl suberate (2 mM) for 30 min as described before^25^,^33^, and then mixed with 50 μl 2 × SDS sample buffer, boiled and fractionated on 12% SDS–polyacrylamide gel followed by immunobloting with anti-mouse ASC antibody.

### Confocal microscopy

Cells were grown on coverslips in 12-well plates. After various treatments, cells were fixed with 2% formaldehyde and stained with Hoechst 33342 (Invitrogen), The coverslips were mounted on slides and then examined using a confocal laser microscope (Nikon C1 plus, Bioimaging Shared Resource of the Kimmel Cancer Center (NCI 5 P30 CA-56036)).

### Statistics

Statistical analyses were made with Student's *t*-test.

## Additional information

**How to cite this article:** Kang, S. *et al.* Caspase-8 scaffolding function and MLKL regulate NLRP3 inflammasome activation downstream of TLR3. *Nat. Commun.* 6:7515 doi: 10.1038/ncomms8515 (2015).

## Supplementary Material

Supplementary Information Supplementary Figures 1-52
