# Supplementary material for: Caspase-8 scaffolding function and MLKL regulate NLRP3 inflammasome activation downstream of TLR3
Source: Nat Commun. 2015 Jun 24;6:7515. doi: 10.1038/ncomms8515 (PMC4480782; doi:10.1038/ncomms8515)
Supplement: Supplementary Information — Supplementary Figures 1-52 [file ncomms8515-s1.pdf]

Supplementary Information

Supplementary Figures

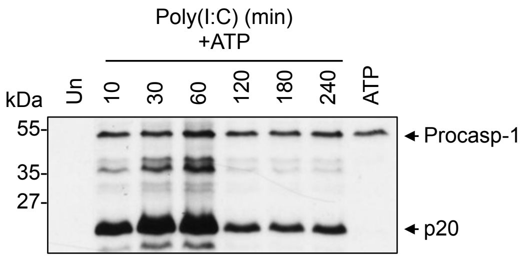

Supplementary Figure 1: Time course analysis of caspase-1 activation in response to Poly(I:C) and ATP. Immunoblot of caspase-1 in the culture supernatants of wildtype (WT) C57BL/6 mouse macrophages stimulated with poly(I:C) for the indicated times (min) followed by stimulation with ATP for an additional 45 min. Results are representative of at least three independent experiments.

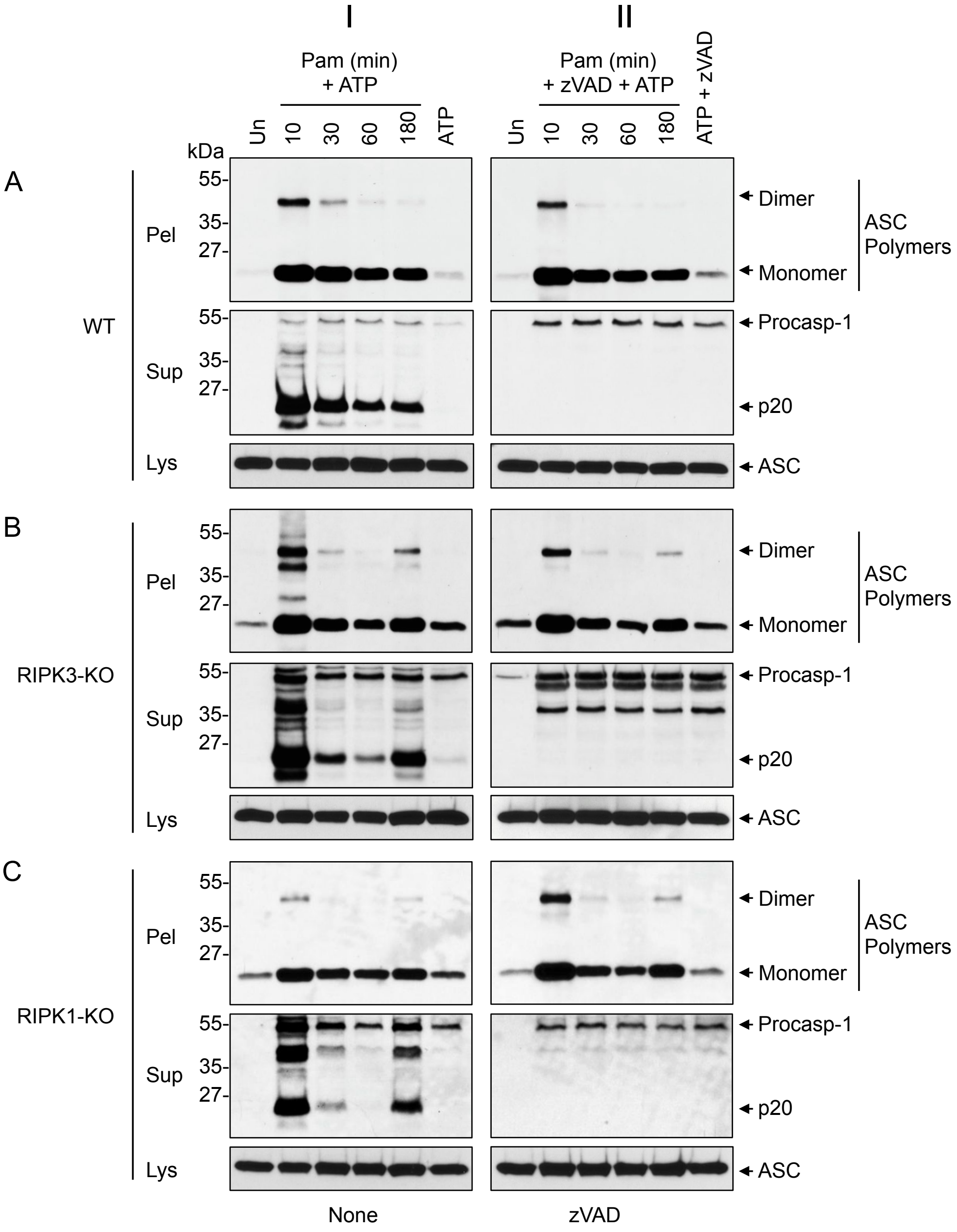

Supplementary Figure 2: TLR2-induced ASC oligomerization does not require caspase-8 enzymatic activity. *A-C* (upper panels), immunoblots of DSS cross-linked ASC in the NP40-insoluble pellets (Pel) of WT (*A*), RIPK3-KO (*B*) and RIPK1-KO (*C*) macrophages after stimulation with Pam3CSK4 (Pam, *I* and *II*) for the indicated times (min) in the absence (*I*) or presence (*II*) of zVAD-FMK followed by stimulation with ATP for an additional 45 min as indicated. Immunoblots of caspase-1 in the culture supernatants (Sup) of the corresponding samples are shown underneath the ASC panels. Immunoblots of total ASC in the cell lysates (Lys) of all samples is shown at the bottom of A-C panels. Results are representative of at least three independent experiments.

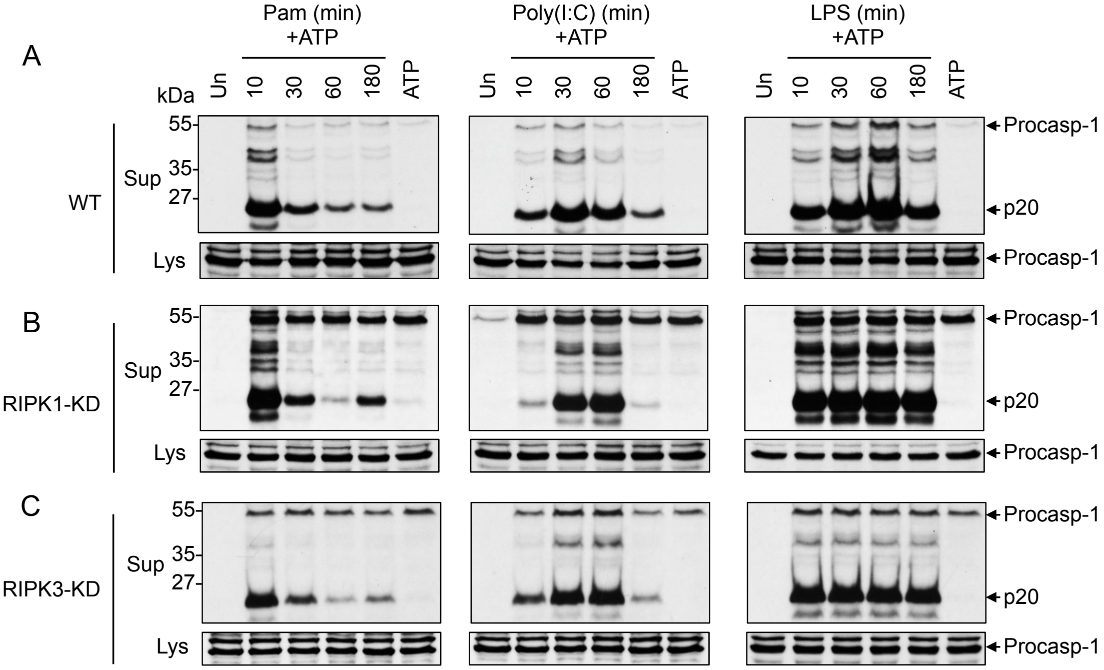

Supplementary Figure 3: Kinase activity of RIPK1 and RIPK3 is dispensable for the early and intermediate pathways of NLRP3 inflammasome activation. *A-C*, immunoblots of caspase-1 in the culture supernatants (Sup) or cell lysates (Lys) of mouse macrophages derived from wildtype (*A*, WT), kinase-dead RIPK1 (*B*, RIPK1-KD) or RIPK3 (*C*, RIPK3-KD) treated with Pam3CSK4 (Pam), poly(I:C) (Poly) or LPS for the indicated times (min) followed by stimulation with ATP for an additional 45 min. Results are representative of at least three independent experiments.

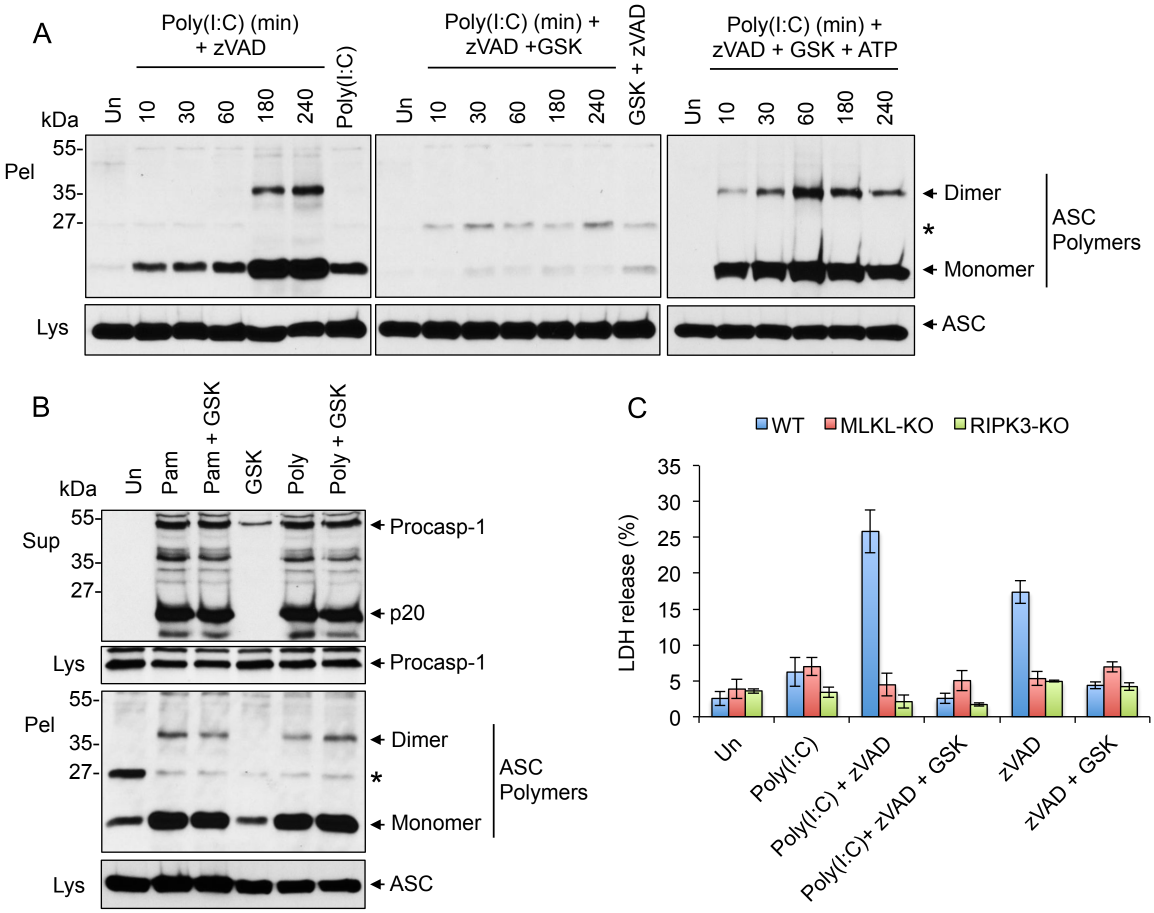

Supplementary Figure 4: Kinase activity of RIPK3 is important for the late but not for the early or intermediate pathway of NLRP3 inflammasome activation. *A,* immunoblots of DSS cross-linked ASC in the NP40-insoluble pellets of WT macrophages after stimulation with poly(I:C) for the indicated times (min) in the presence of zVAD-FMK (*left panel*), zVAD-FMK and GSK'872 (*middle panel*), or zVAD-FMK followed by stimulation with ATP for an additional 45 min (*right panel*) as indicated. *B*, immunoblots of caspase-1 in the culture supernatants (Sup, *upper panel*) or cell lysates (Lys, *middle panel*) of WT mouse macrophages treated with Pam3CSK4 (Pam) for 10 min or Poly(I:C) (Poly) for 45 min in the absence or presence of GSK'872 as indicated followed by stimulation with ATP for an additional 45 min. The lower panel shows an immunoblot of DSS cross-linked ASC in the NP40-insoluble pellets (Pel) of the same samples above. Asterisks indicate non-specific bands. *C*, LDH release in the culture supernatants of macrophages derived from WT, RIPK3-KO or MLKL-KO mice treated with the indicated stimuli for 5 h. Results are representative of at least three independent experiments. Error bars represent standard deviations.

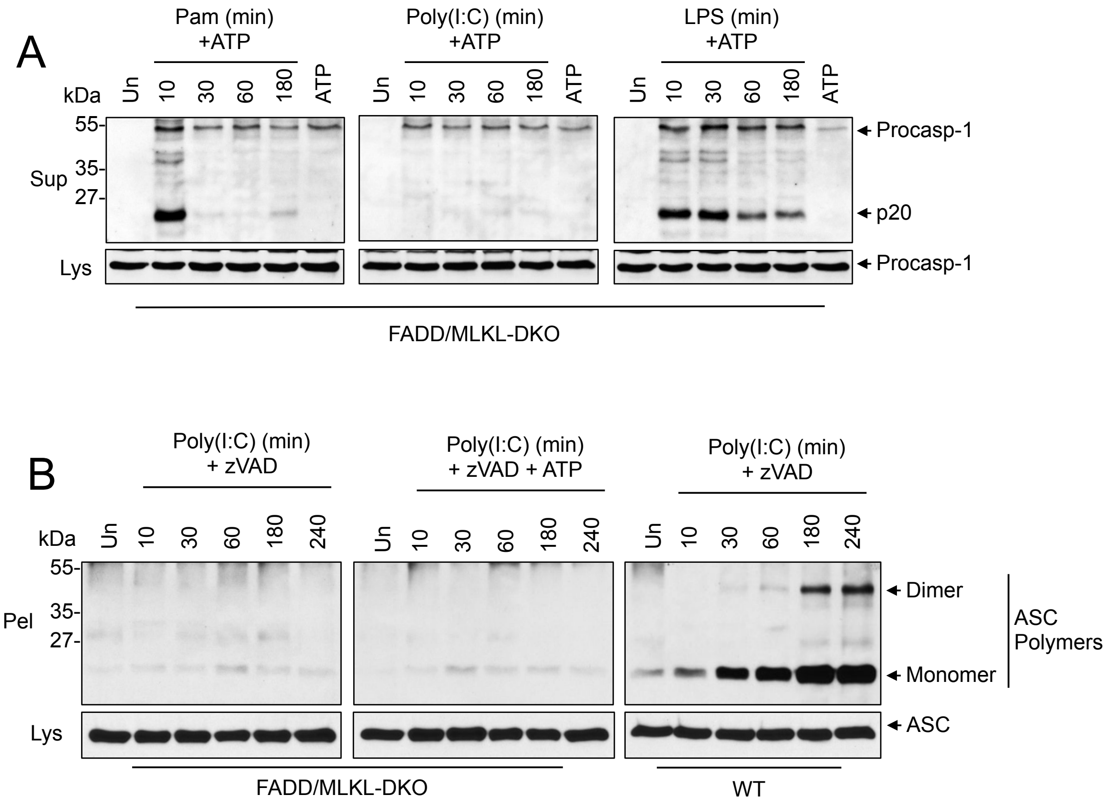

Supplementary Figure 5: TLR-induced inflammasome activation in FADD/MLKL-DKO macrophages. *A*, immunoblots of caspase-1 in the culture supernatants (Sup) or cell lysates (Lys) of mouse macrophages derived from WT or FADD/MLKL-DKO mice treated with Pam3CSK4 (Pam), poly(I:C) (Poly) or LPS for the indicated times (min) followed by stimulation with ATP for an additional 45 min. *B,* immunoblots of DSS cross-linked ASC in the NP40-insoluble pellets of FADD-MLKL-KO macrophages after stimulation with poly(I:C) for the indicated times (min) in the presence of zVAD-FMK (*left panels*) or zVAD-FMK followed by stimulation with ATP for an additional 45 min (*middle panels*) as indicated. WT immunoblots (*right panels*) are shown as a positive control. Immunoblots of total ASC in the cell lysates (Lys) of all samples is shown at the bottom. Results are representative of at least three independent experiments.

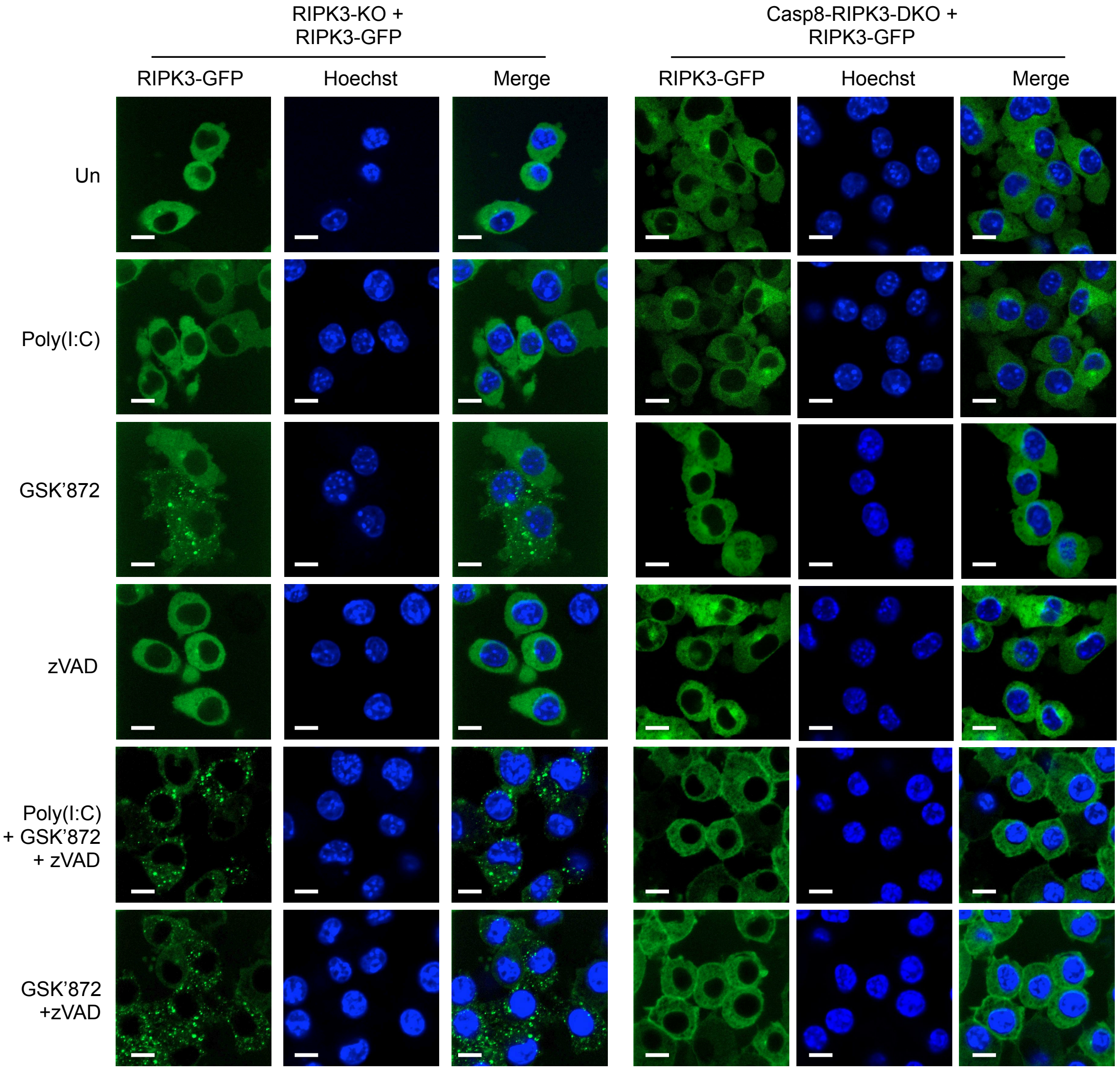

Supplementary Figure 6: Caspase-8 scaffolding function is required for GSK'872 to induce RIPK3-GFP aggregation. Confocal images of unstimulated (Un), poly(I:C), GSK'872, zVAD-FMK, poly(I:C) plus zVAD-FMK plus GSK'872 or GSK'872 plus zVAD-FMK-stimulated stable RIPK3-GFP-reconstituted RIPK3-KO (RIPK3-KO + RIPK3-GFP) or caspase-8-RIPK3-DKO (Casp8/RIPK3-DKO + RIPK3-GFP) macrophages. Stimulation times were 180 min. Results are representative of at least three independent experiments. Bar: 10 µm.

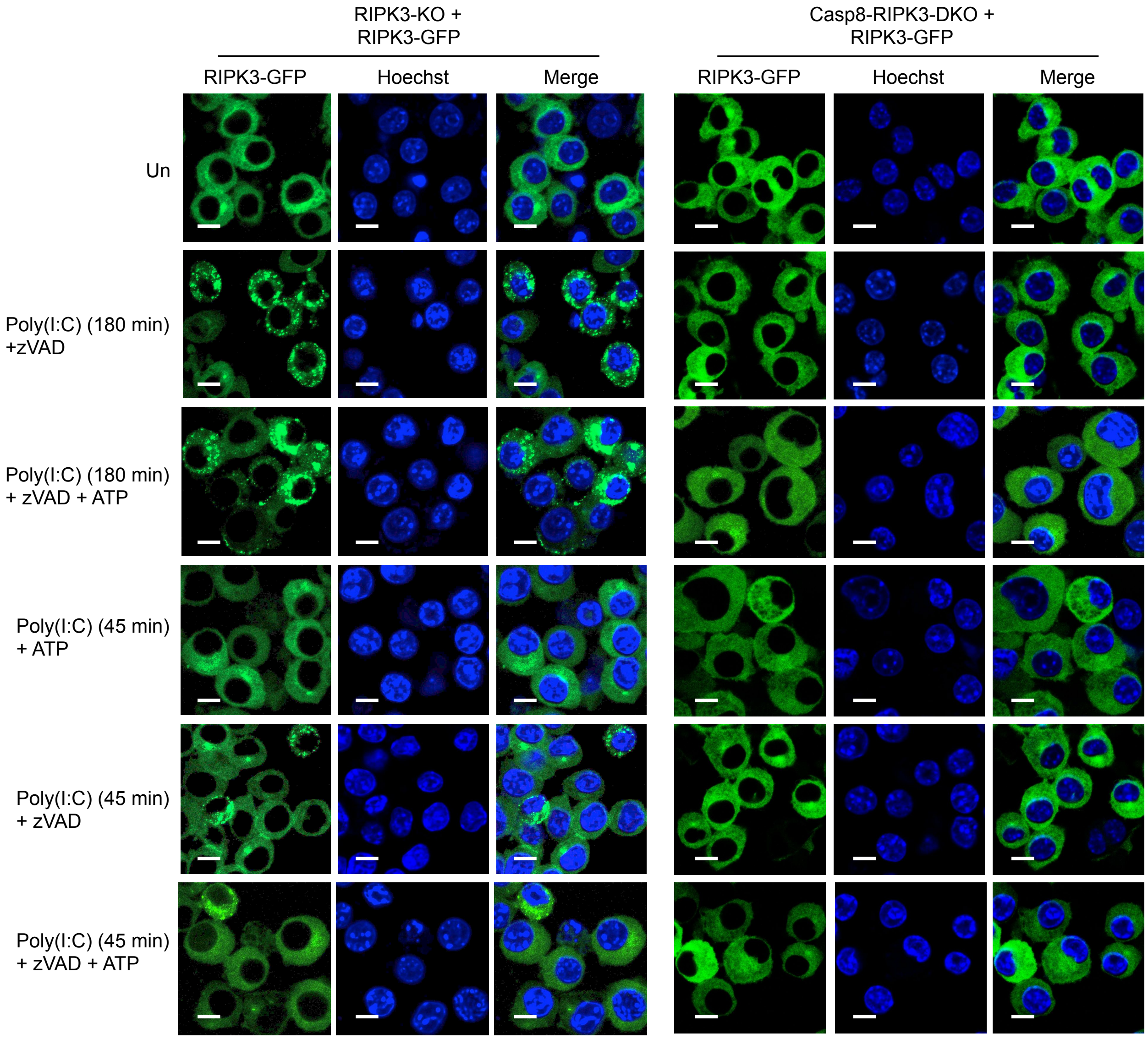

Supplementary Figure 7: ATP does not stimulate RIPK3 oligomerization. Confocal images of stable RIPK3-GFP-reconstituted RIPK3-KO (RIPK3-KO + RIPK3-GFP) or caspase-8-RIPK3-DKO (Casp8/RIPK3-DKO + RIPK3-GFP) macrophages stimulated with the indicated stimuli for the indicated times in minutes. Results are representative of at least three independent experiments. Bar: 10 µm.

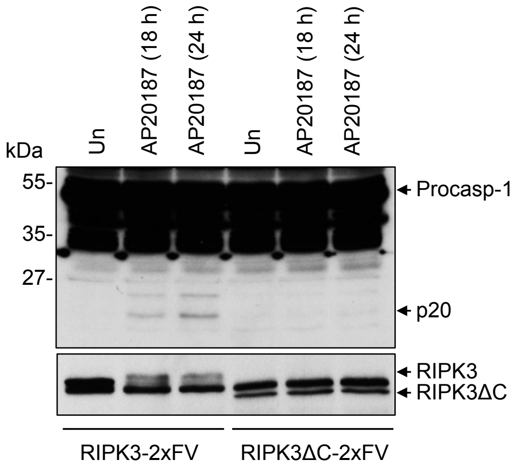

Supplementary Figure 8: Enforced oligomerization of RIPK3 induces NLRP3 inflammasome activation in reconstituted 293TCAN cells. RIPK3 or RIPK3-ΔRHIM fused to two copies of $FKBP^{F36V}$ (RIPK3-2xFV or RIPK3-ΔRHIM-2xFV, respectively) were stably expressed in 293T-CAN cell line which is stably reconstituted with the human NLRP3 inflammasome components procaspase-1, ASC, and NLRP3. Caspase-1 immunoblot of cell lysates shows caspase-1 p20 band only in RIPK3-2xFV-expressing, but not in RIPK3-ΔRHIM-2xFV-expressing 293T-CAN cells after stimulation with the oligomerization drug AP20187. Note that AP20187 stimulation causes an upshift in the RIPK3-2xFV bands but not in the RIPK3-ΔRHIM-2xFV bands, indicating posttranslational modification of RIPK3-2xFV protein after oligomerization. Results are representative of at least three independent experiments.

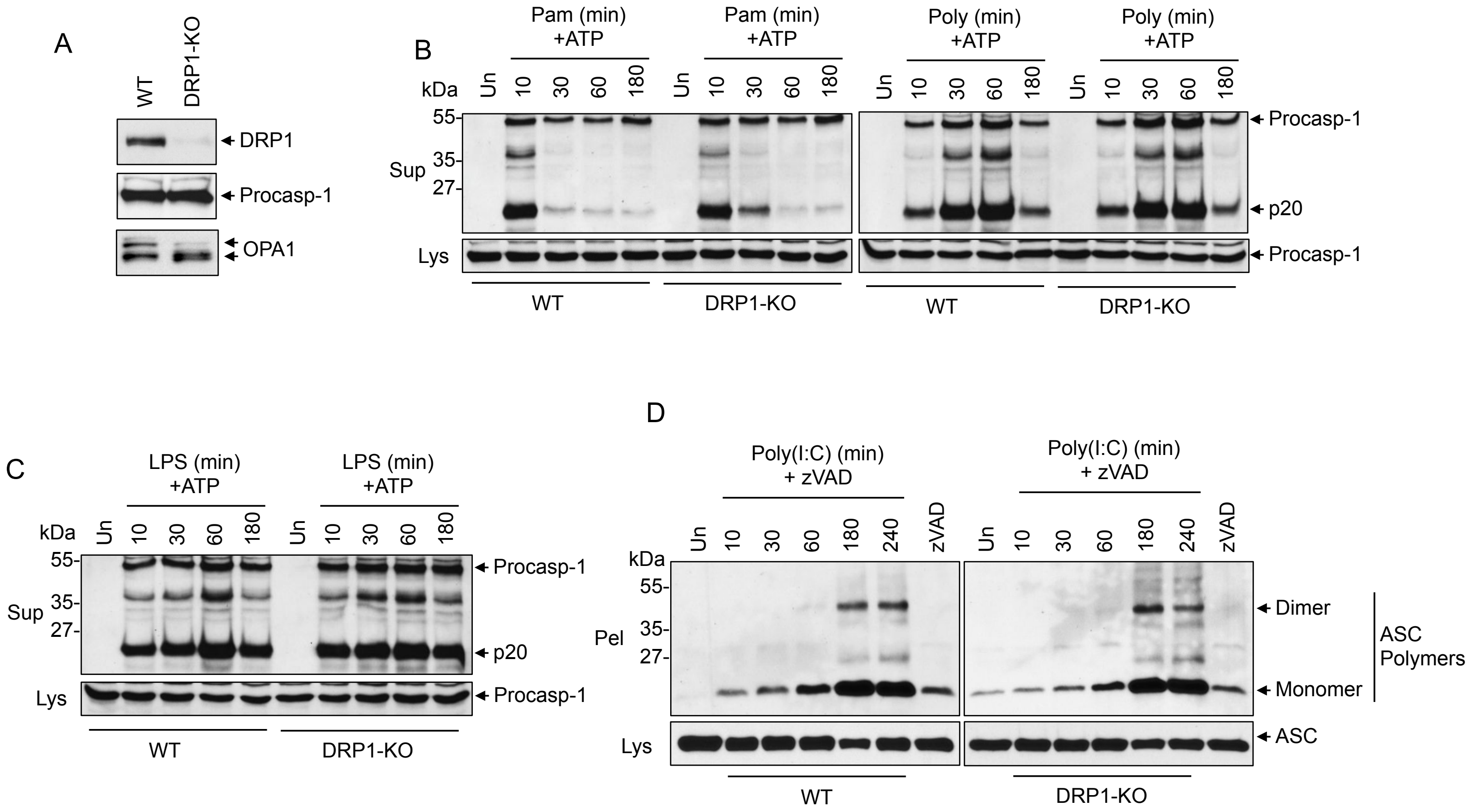

Supplementary Figure 9: Normal inflammasome activation in DRP1-KO macrophages. *A*, Immunoblots of DRP1, caspase-1 and OPA1 in cell lysates of mouse macrophages derived from WT or Drp1$^{fl/fl}$-LysMCre mice. *B-C*, immunoblots of caspase-1 in the culture supernatants (Sup) or cell lysates (Lys) of mouse macrophages derived from WT or Drp1$^{fl/fl}$-LysMCre (DRP1-KO) mice treated with Pam3CSK4 (Pam), poly(I:C) (Poly) or LPS for the indicated times (min) followed by stimulation with ATP for an additional 45 min. *D*, immunoblots of DSS cross-linked ASC in the NP40-insoluble pellets of WT or DRP1-KO macrophages after stimulation with poly(I:C) for the indicated times (min) in the presence of zVAD-FMK as indicated. . Immunoblots of total ASC in the cell lysates (Lys) of all samples is shown at the bottom. Results are representative of at least three independent experiments.

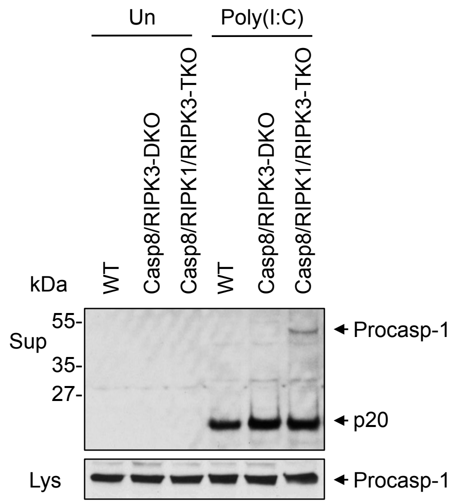

Supplementary Figure 10: Cytoplasmic dsRNA induces normal inflammasome activation in Casp8/RIPK3-DKO and Casp8/RIPK1/RIPK3-TKO macrophages. Immunoblots of caspase-1 in the culture supernatants (Sup) or cell lysates (Lys) of WT or the indicated knockout primary macrophages transfected with poly(I:C) for 5 h as indicated. Results are representative of at least three independent experiments.

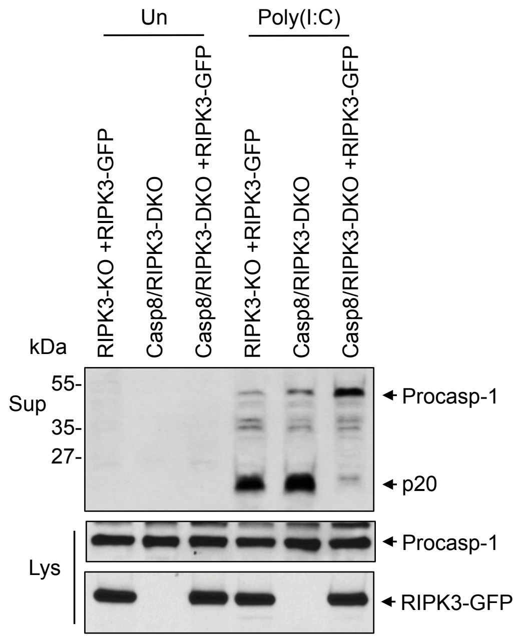

Supplementary Figure 11: Cytoplasmic dsRNA requires caspase-8 to activate the inflammasome when RIPK3 is present. Immunoblots of caspase-1 in the culture supernatants (Sup) or cell lysates (Lys) of stable RIPK3-GFP-reconstituted RIPK3-KO (RIPK3-KO + RIPK3-GFP) or caspase-8-RIPK3-DKO (Casp8/ RIPK3-DKO + RIPK3-GFP) immortalized macrophages transfected with poly(I:C) for 5 h as indicated. Results are representative of at least three independent experiments.

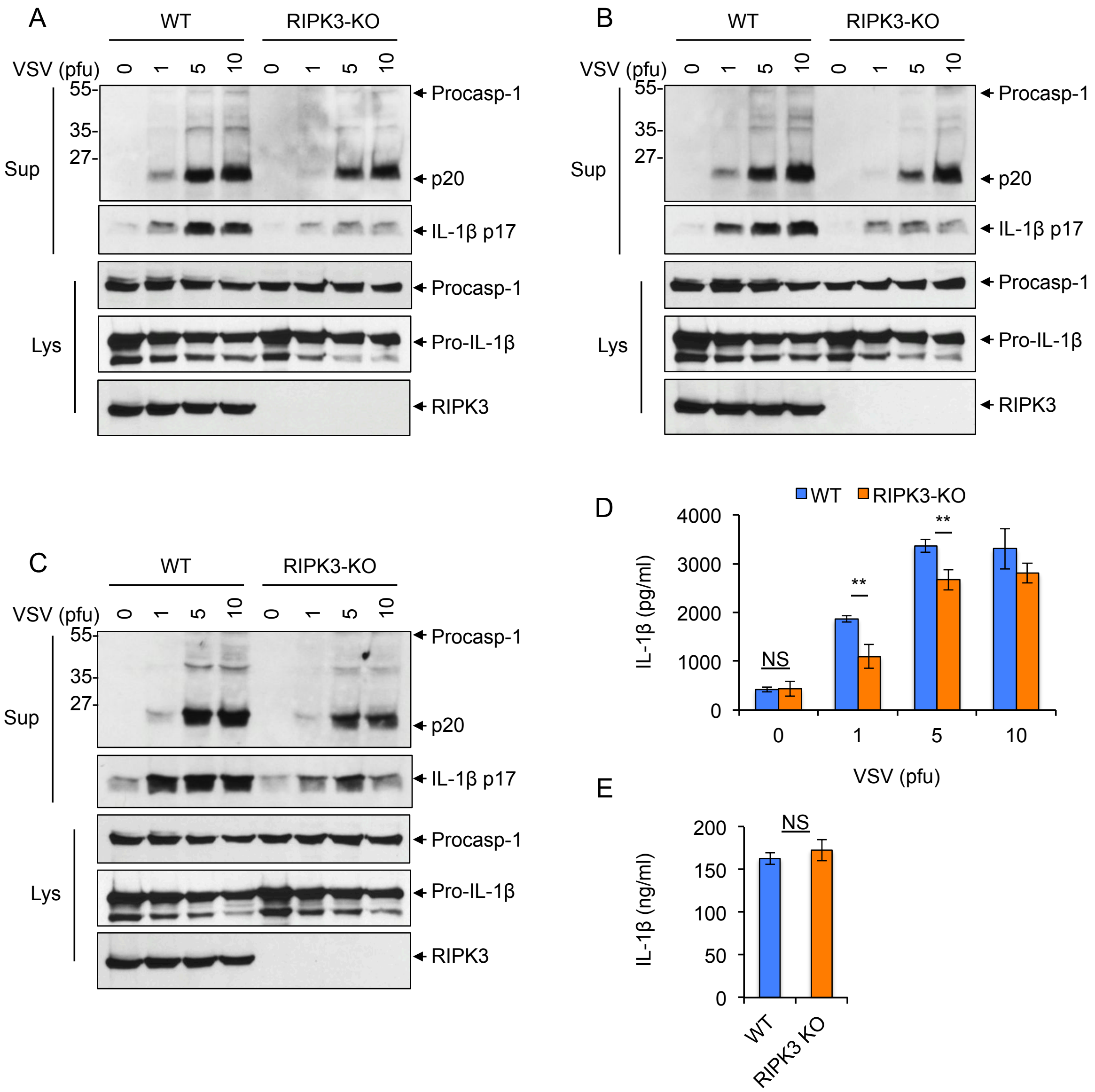

Supplementary Figure 12: VSV-induced NLRP3 activation is partially dependent on RIPK3. *A-C,* immunoblots of caspase-1 (1st *panels from top)* and mature IL-1β p17 (2nd *panels from top)* in the culture supernatants (Sup) of macrophages derived from WT (n=3) or RIPK3-KO (n=3) mice after infection with the indicated doses of VSV (plaque forming units, pfu) for 16 h. Immunoblots of procaspase-1, pro-IL-1β and RIPK3 in the total cell lysates are shown underneath the supernatants blots. *D,* ELISA quantitation of IL-1β in the culture supernatants of macrophages derived from WT (n=3) or RIPK3-KO (n=3) mice after infection with the indicated doses of VSV for 16 h. *E,* ELISA quantitation of total IL-1β in cell lysates of the uninfected macrophages shown in D. Error bars represent standard deviations. NS, not significant ($P > 0.5$). **$P < 0.01$ (student *t* test).

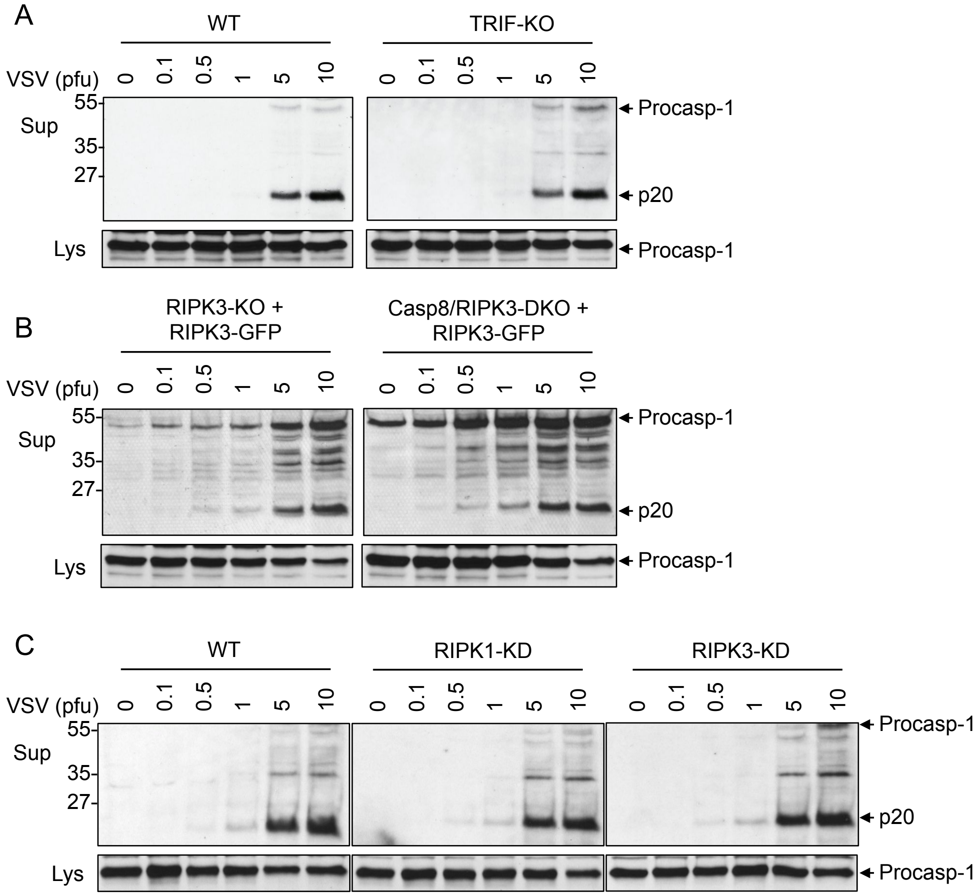

Supplementary Figure 13: VSV-induced NLRP3 activation does not require TRIF, caspase-8 or kinase activity of RIPK1 or RIPK3. *A*-C, immunoblots of caspase-1 in the culture supernatants (Sup) or cell lysates (Lys) of WT or the indicated knockout primary macrophages (*A* and *C*,), or stable RIPK3-GFP-reconstituted RIPK3-KO (RIPK3-KO + RIPK3-GFP) or caspase-8-RIPK3-DKO (Casp8/RIPK3-DKO + RIPK3-GFP) immortalized macrophages (*B*) after infection with the indicated doses of VSV (plaque forming units, pfu) for 16 h. Results are representative of at least three independent experiments.

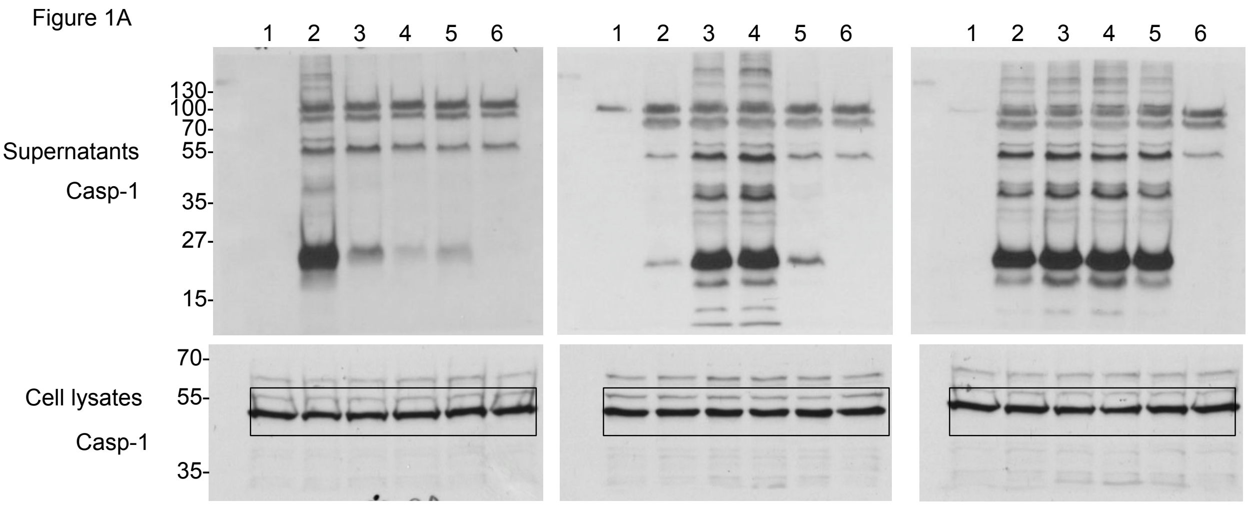

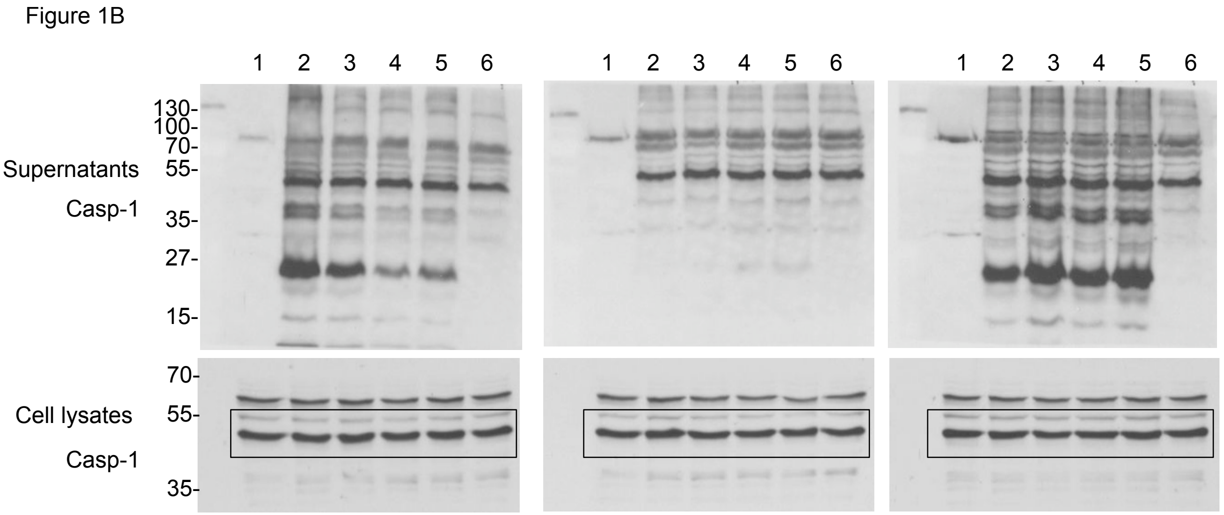

Supplementary Figure 14: full-sized scans of Western blots in Figure 1A, B.

Figure 1C

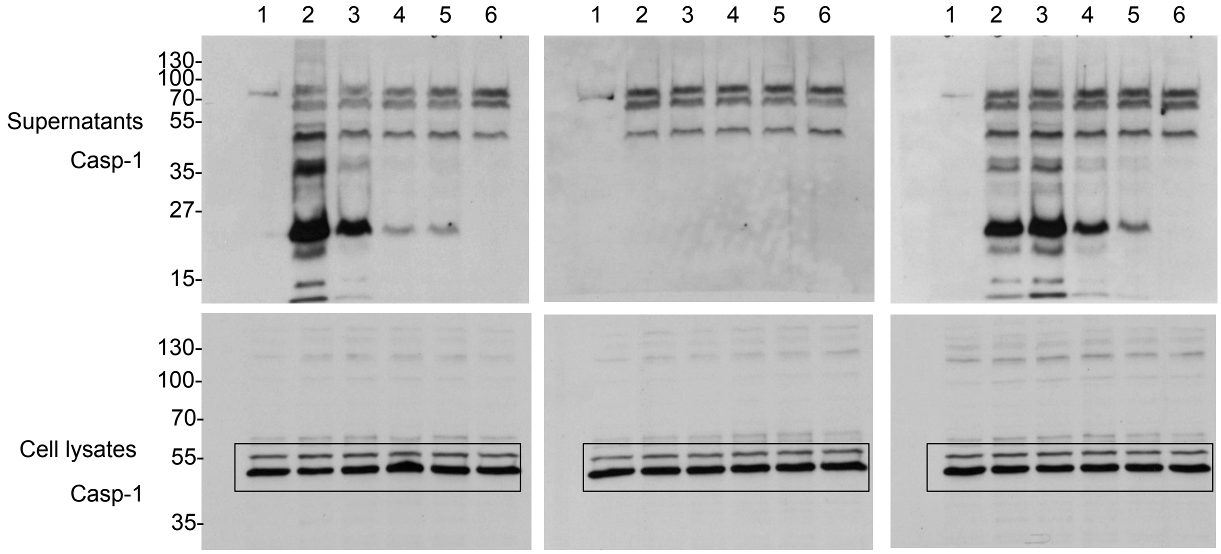

Figure 1D

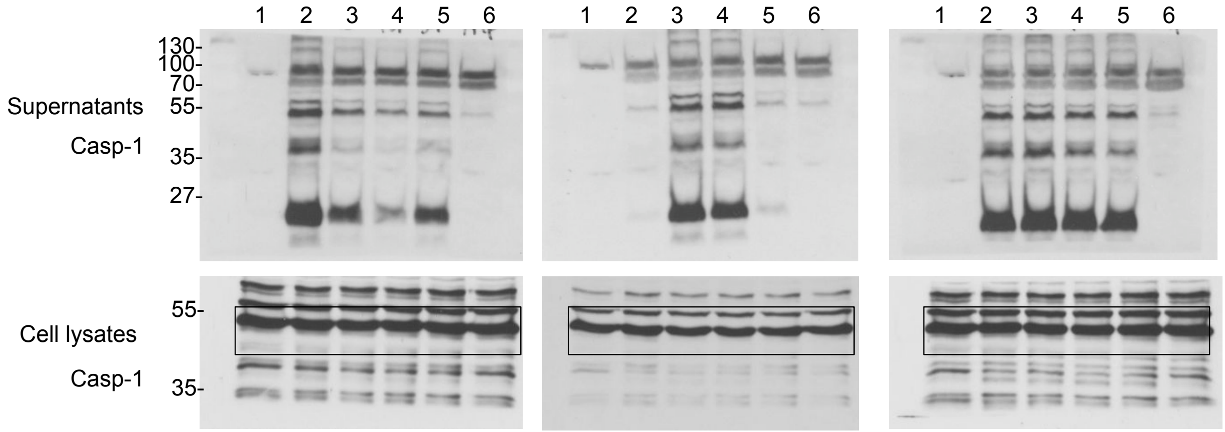

Supplementary Figure 15: full-sized scans of Western blots in Figure 1C, D

Figure 1E

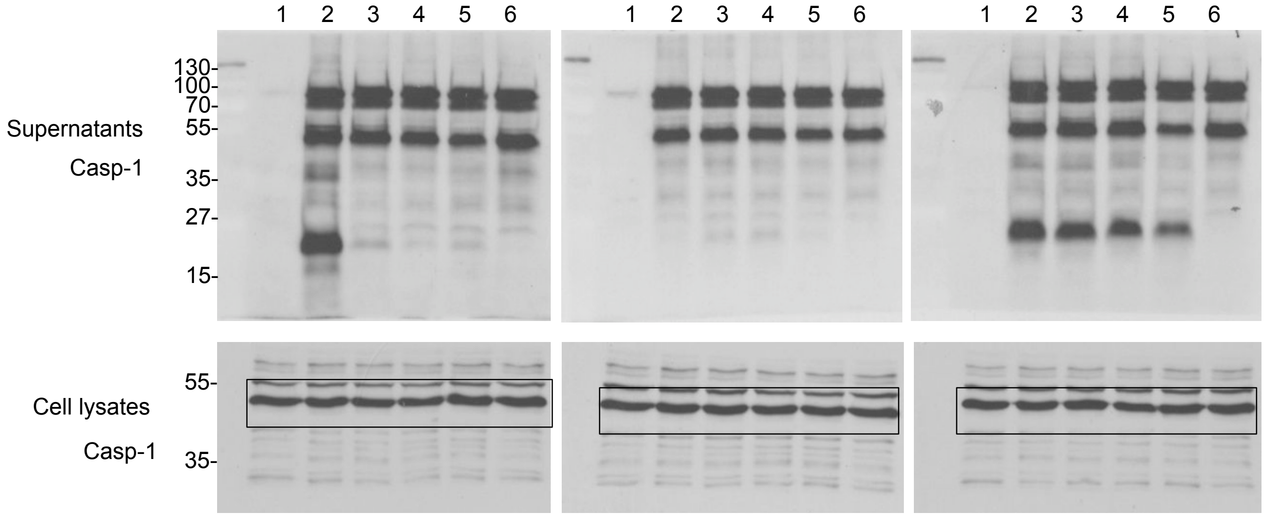

Figure 1F

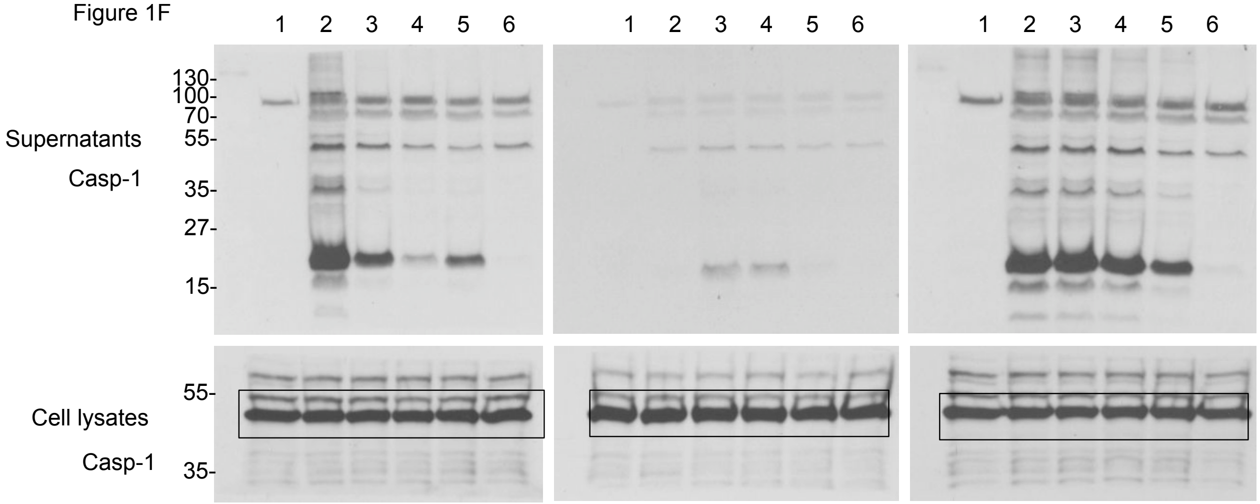

Supplementary Figure 16: full-sized scans of Western blots in Figure 1E, F,

Figure 1G

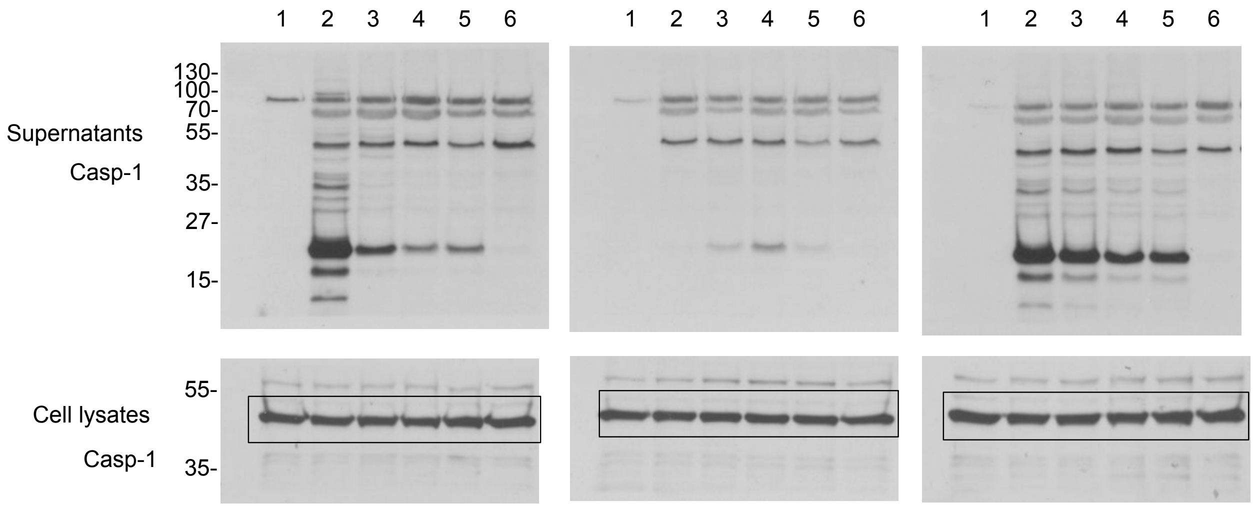

Supplementary Figure 17: full-sized scans of Western blots in Figure 1G

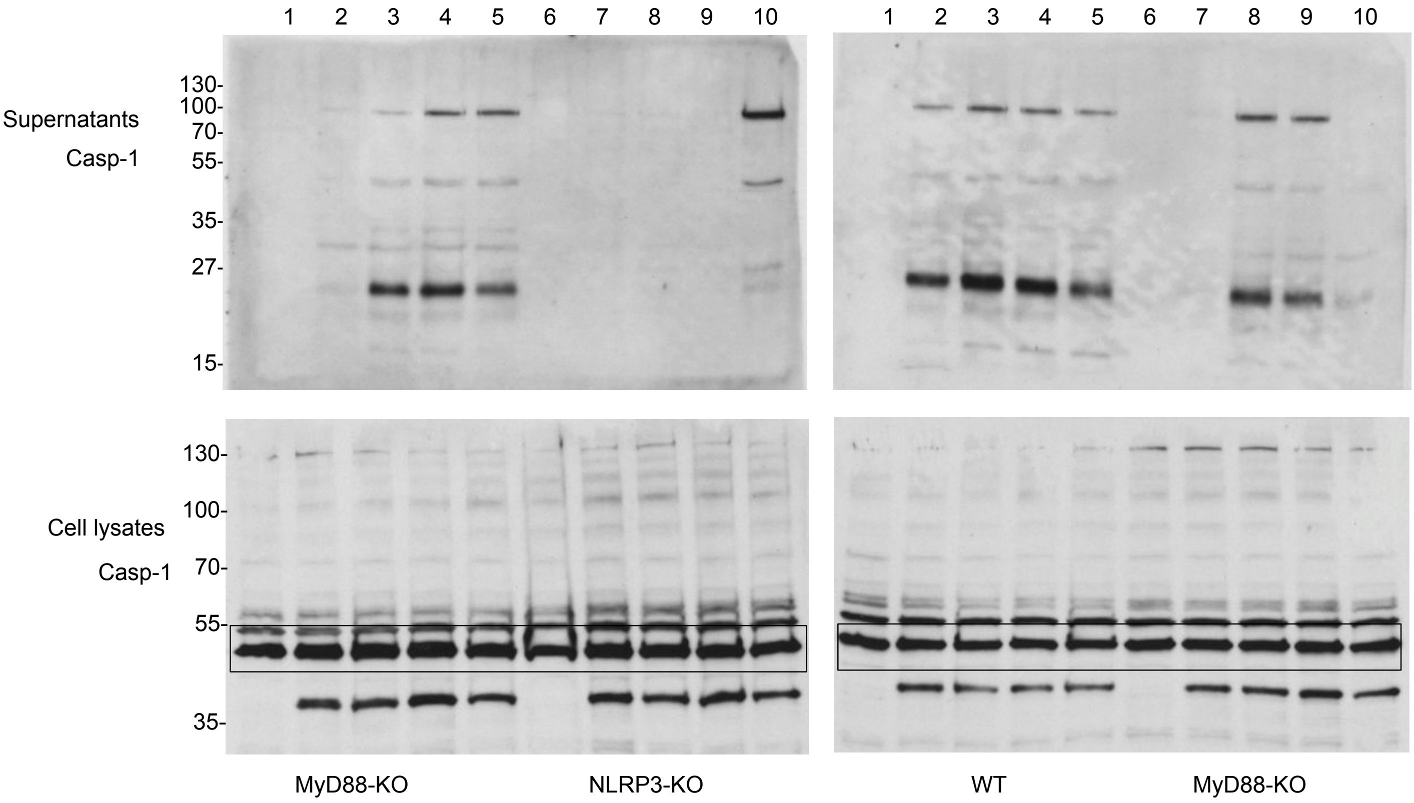

Supplementary Figure 18: full-sized scans of Western blots in Figure 2C

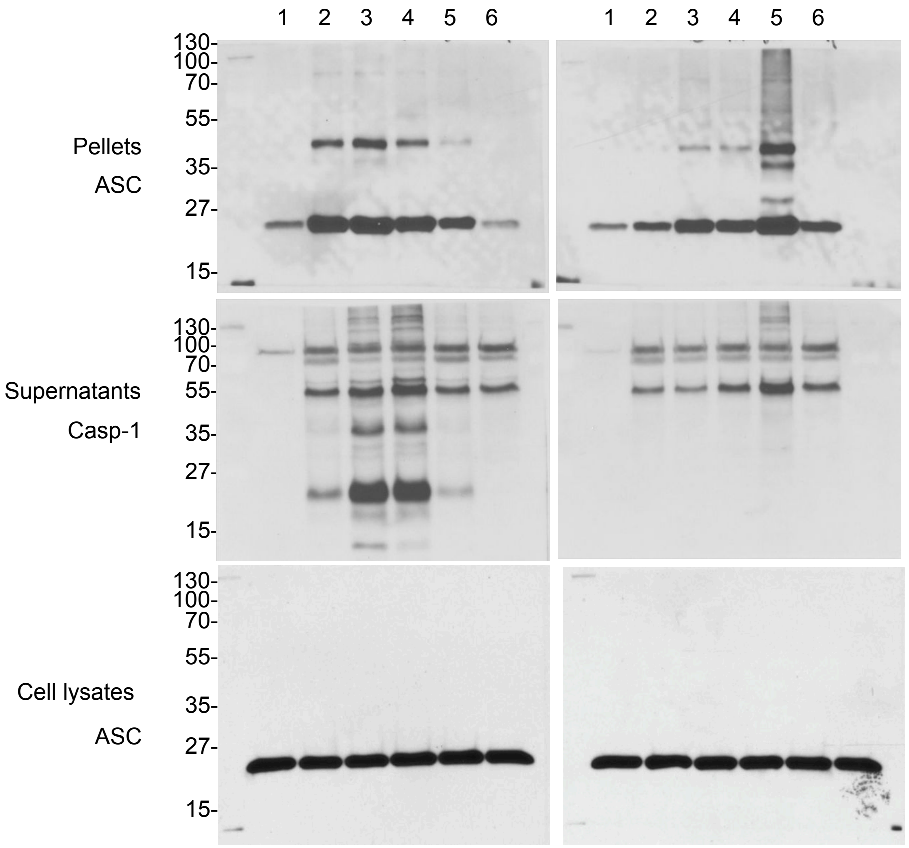

Supplementary Figure 19: full-sized scans of Western blots in Figure 3A

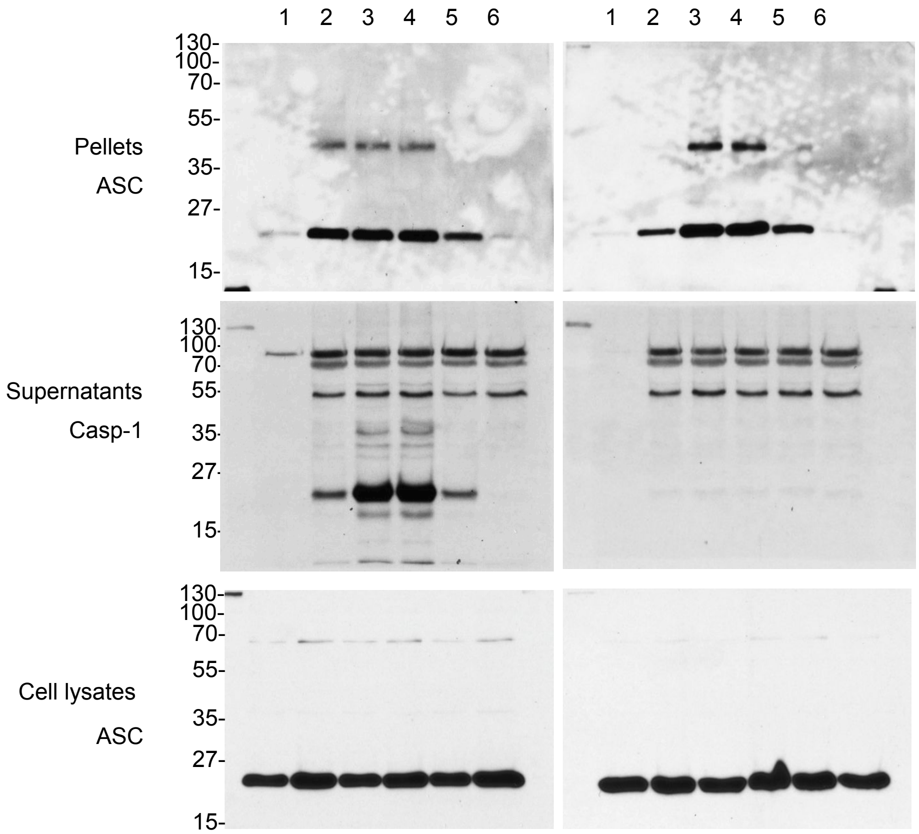

Supplementary Figure 20: full-sized scans of Western blots in Figure 3B

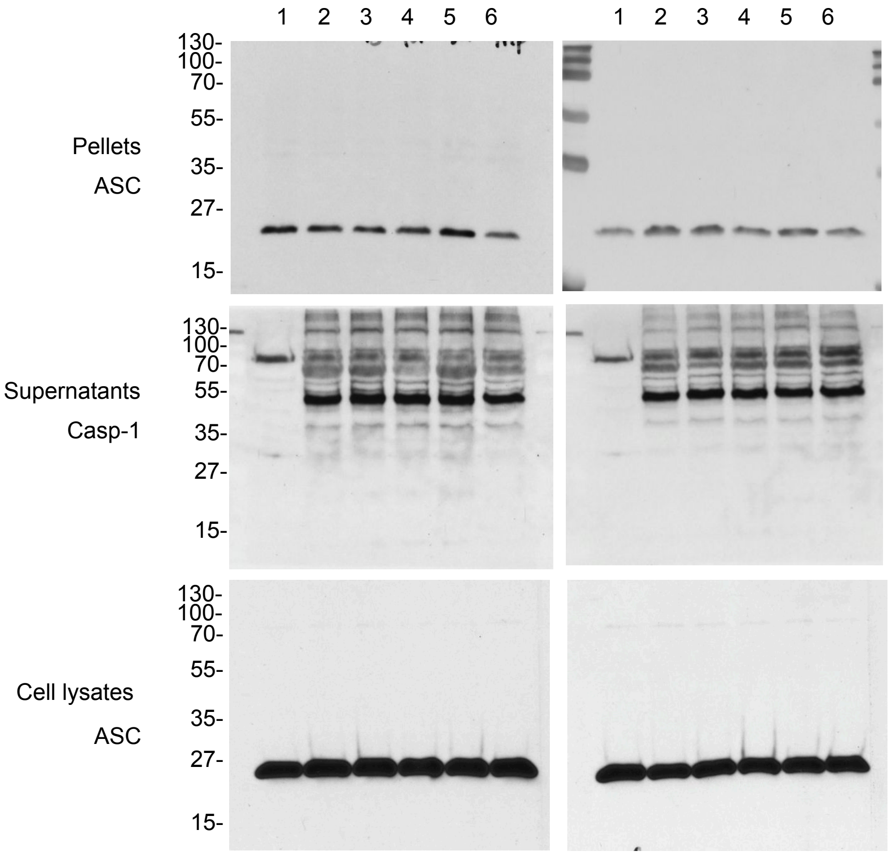

Supplementary Figure 21: full-sized scans of Western blots in Figure 3C

Figure 4A

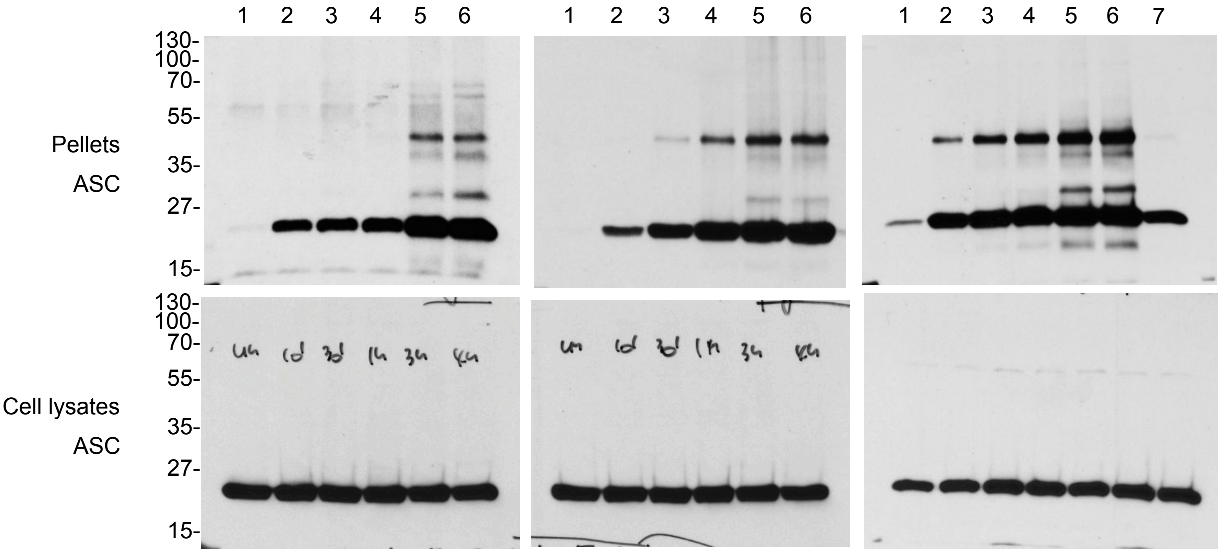

Figure 4B

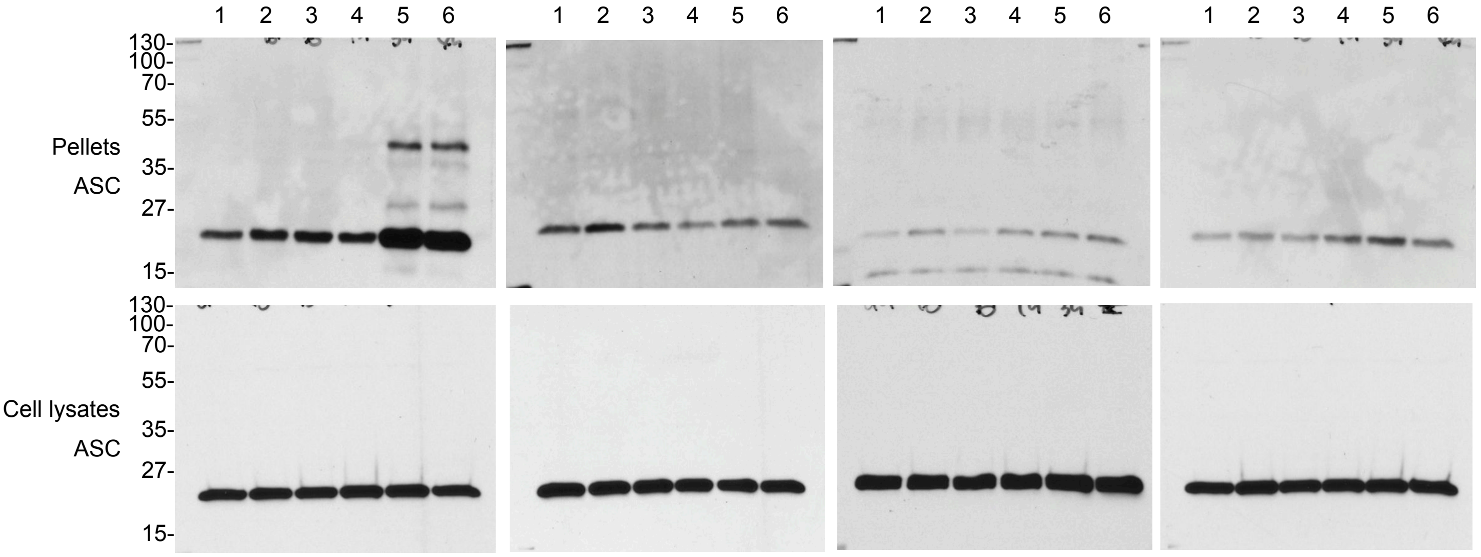

Supplementary Figure 22: full-sized scans of Western blots in Figure 4A, B

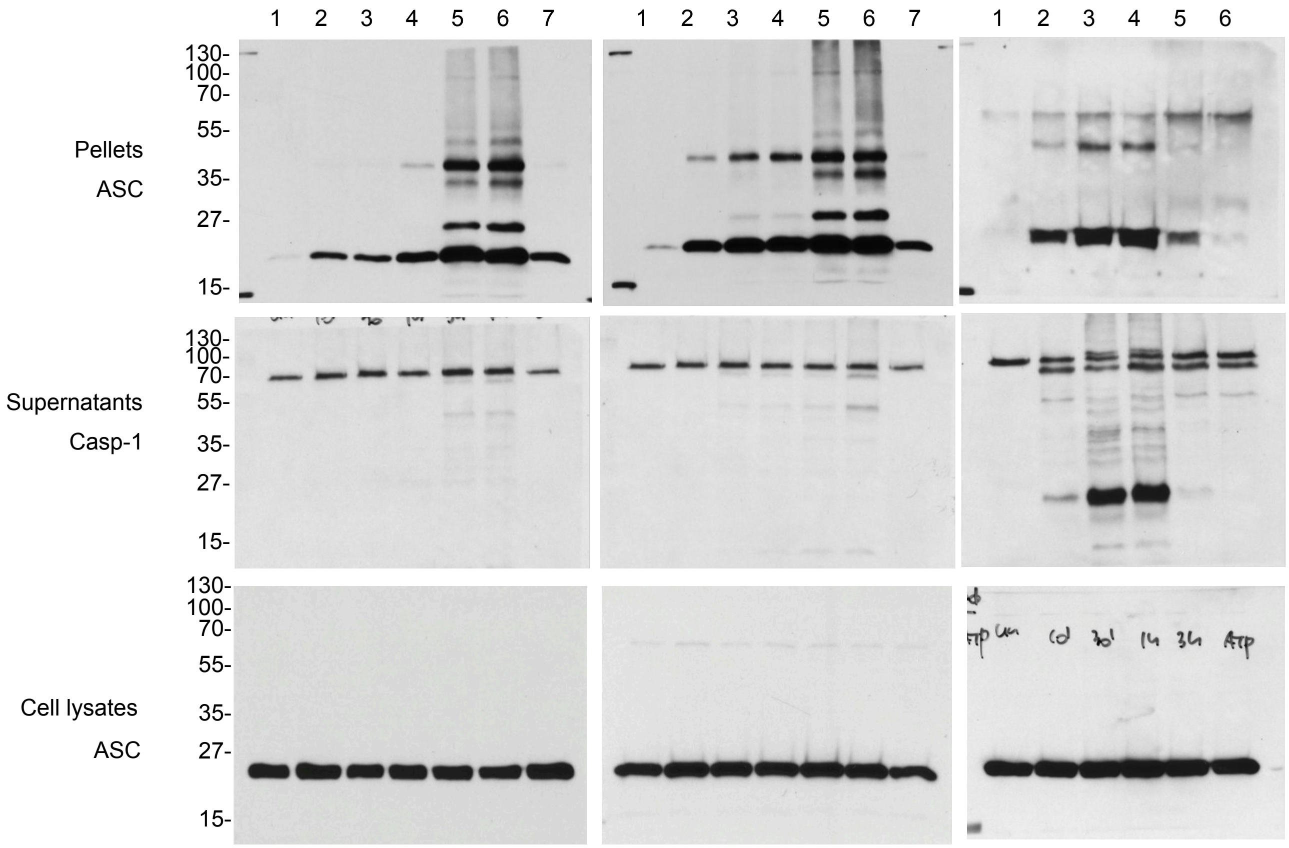

Supplementary Figure 23: full-sized scans of Western blots in Figure 5A

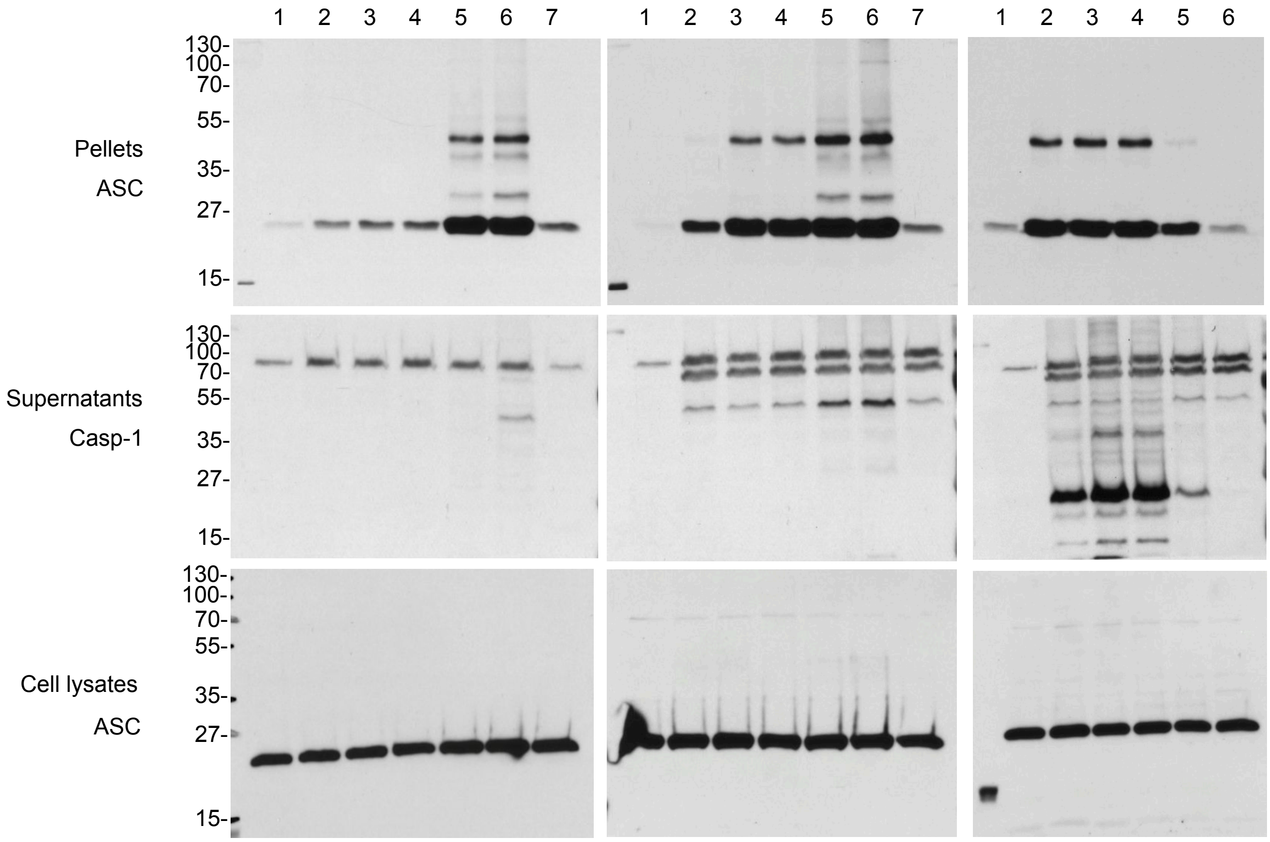

Supplementary Figure 24: full-sized scans of Western blots in Figure 5B

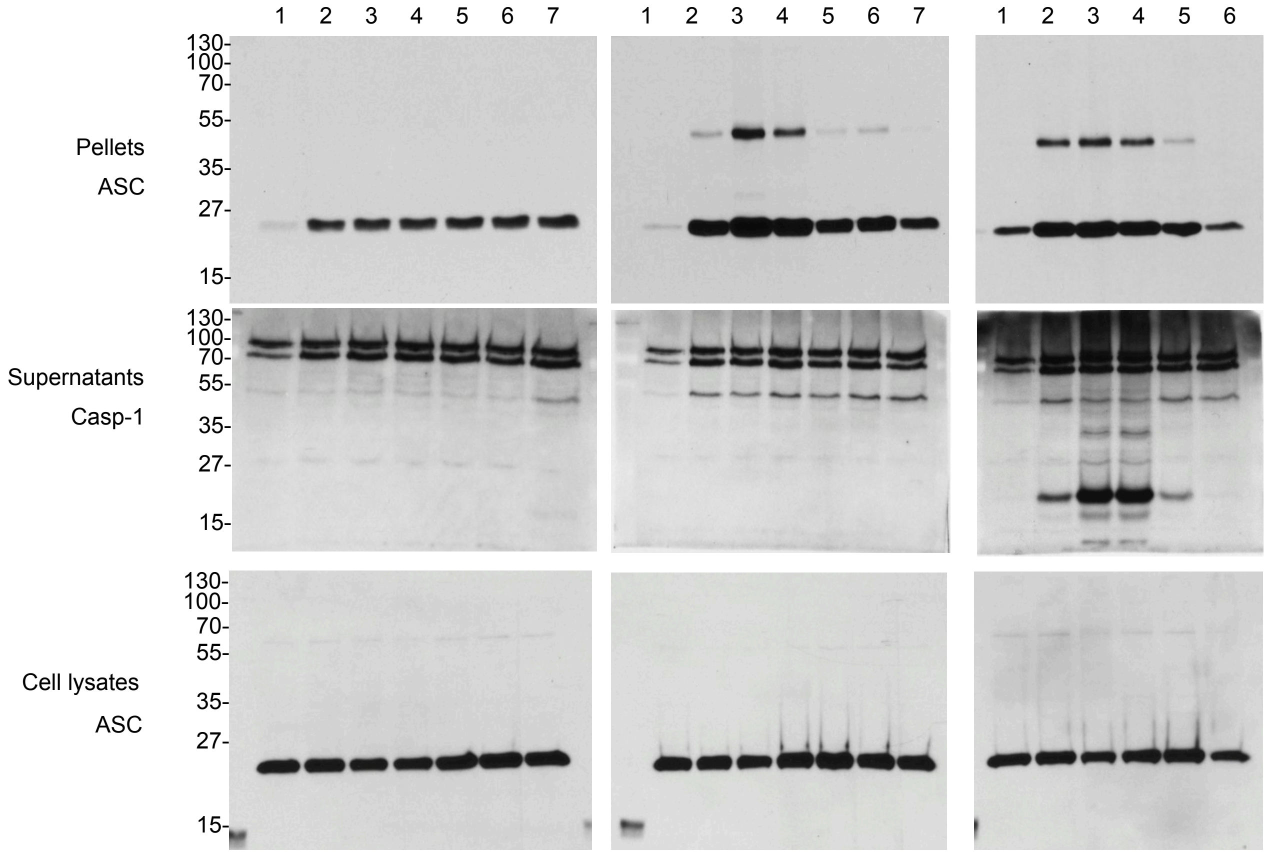

Supplementary Figure 25: full-sized scans of Western blots in Figure 5C

Figure 6A

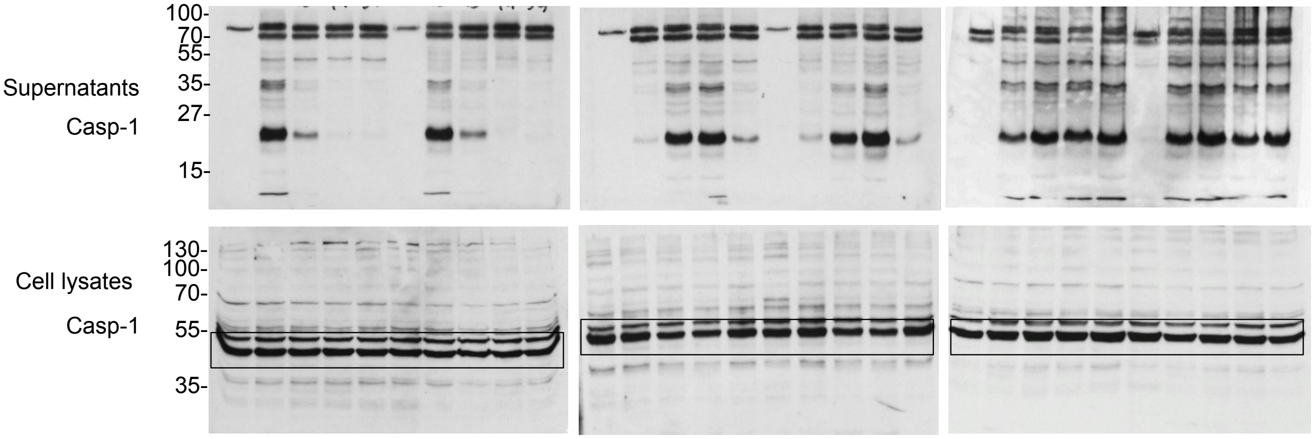

Figure 6B

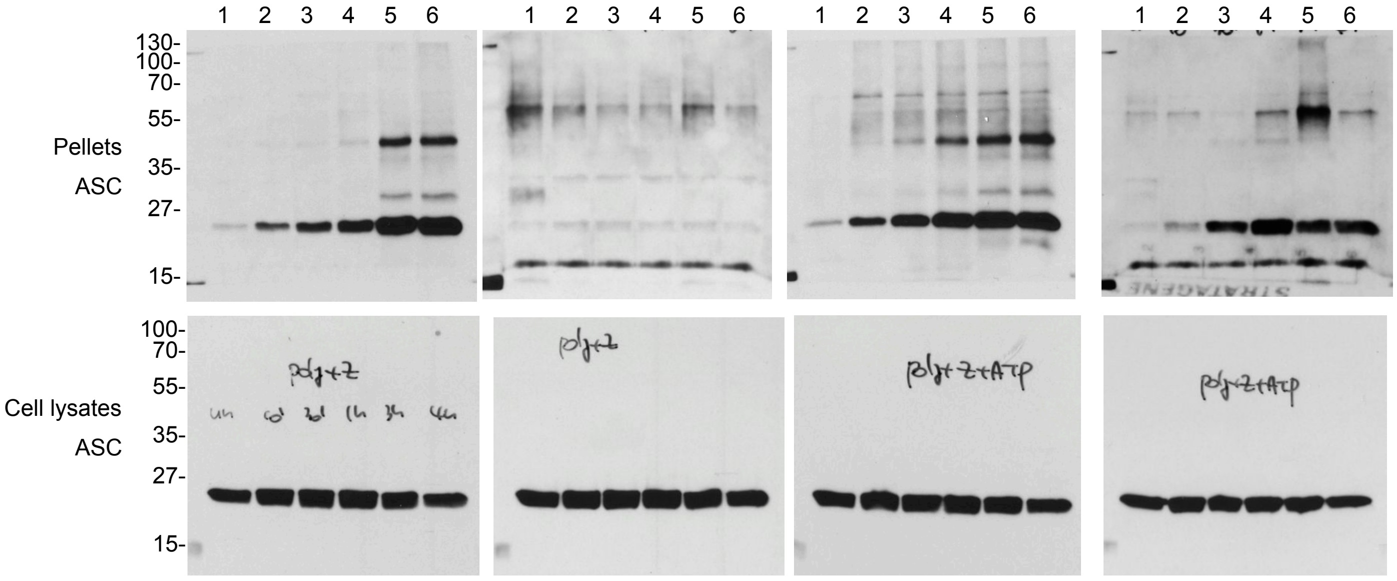

Supplementary Figure 26: full-sized scans of Western blots in Figure 6A, B

Figure 7A

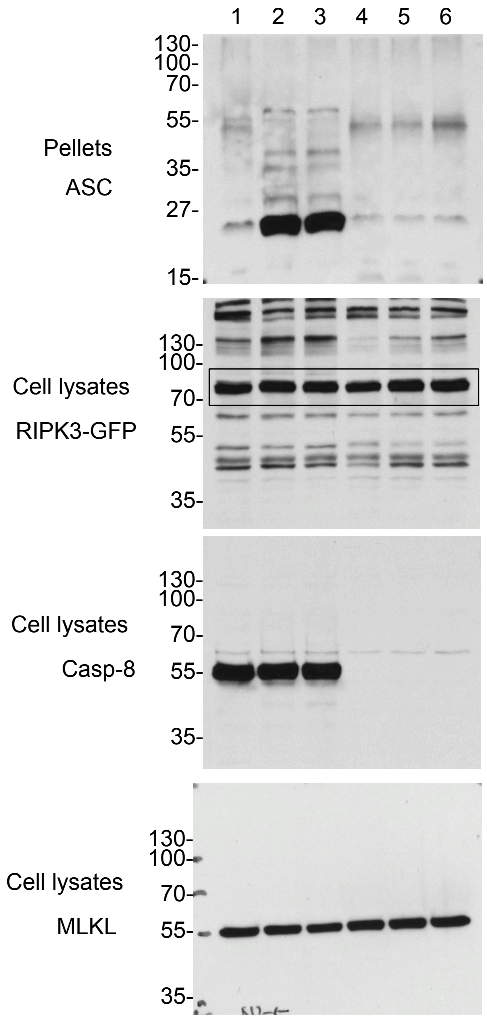

Figure 7C

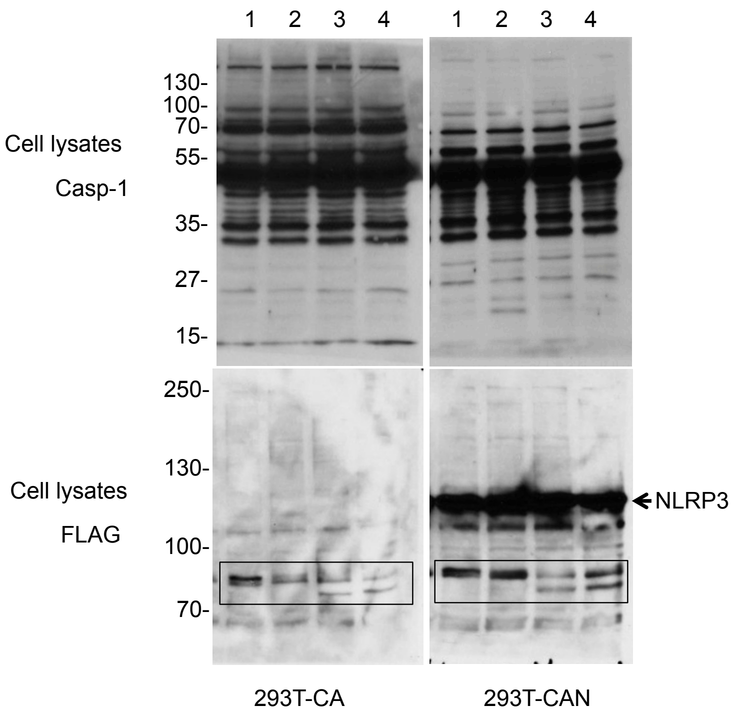

Supplementary Figure 27: full-sized scans of Western blots in Figure 7A, C

Figure 8A

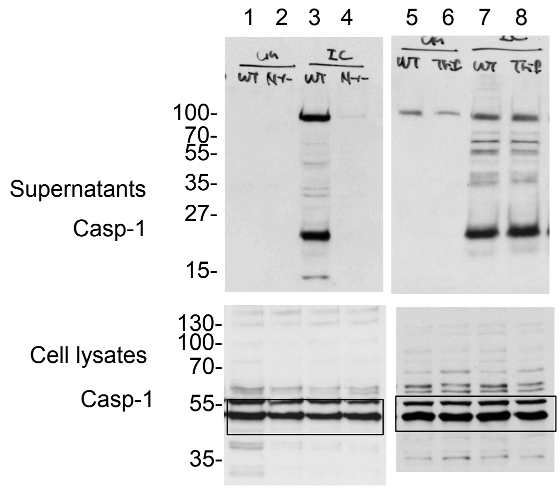

Figure 8B

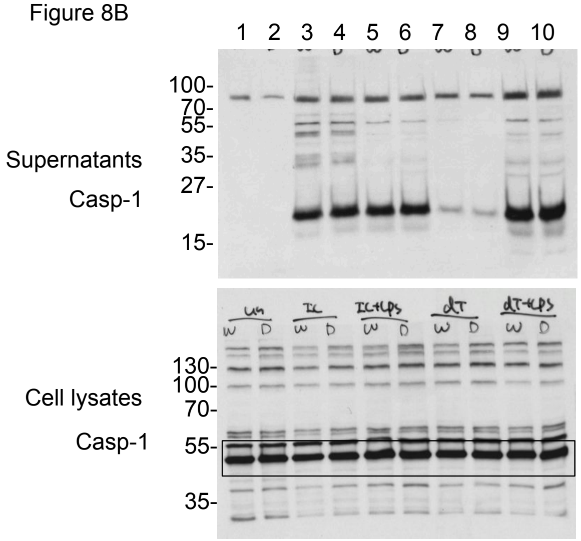

Figure 8C

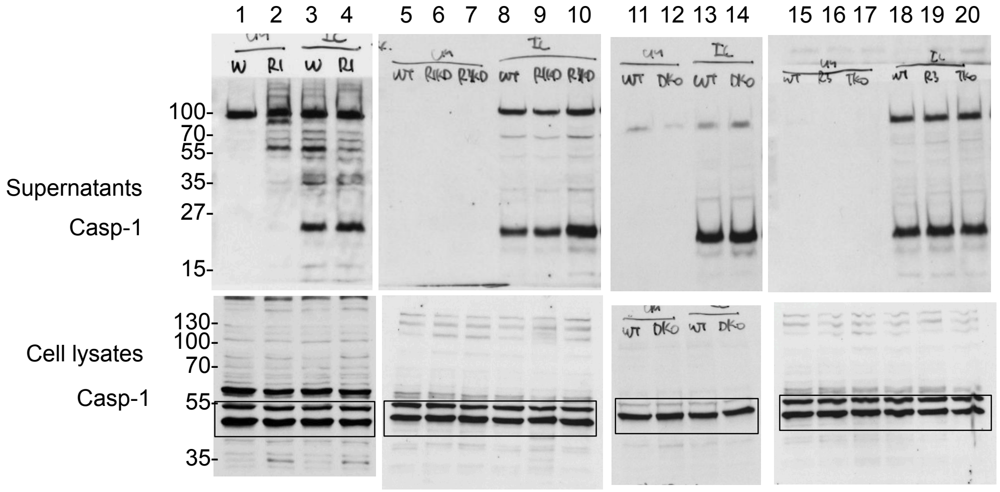

Supplementary Figure 28: full-sized scans of Western blots in Supplementary Figure 8A-C

Figure 8D

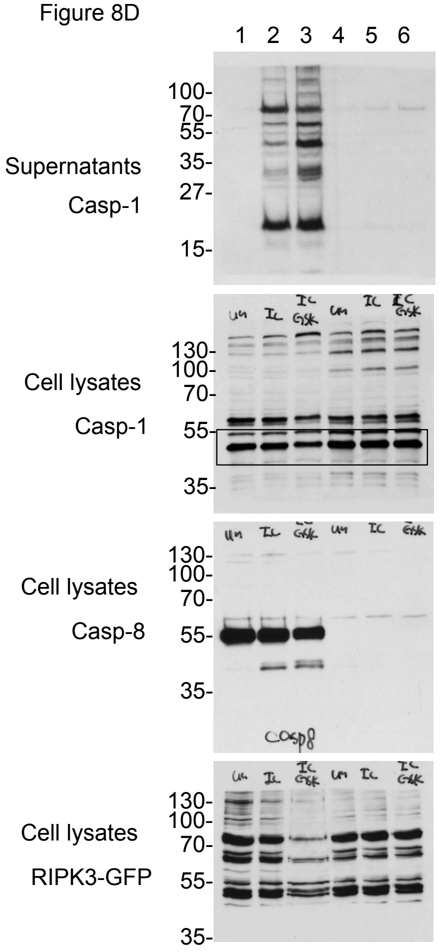

Figure 8E

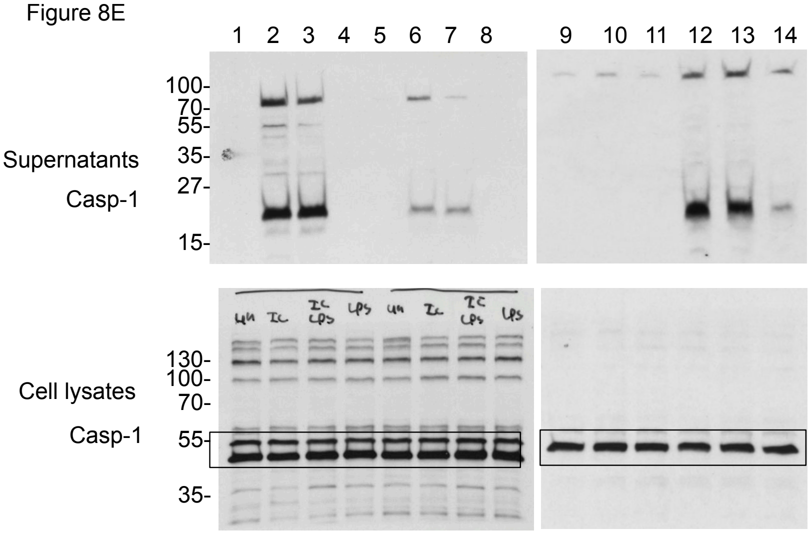

Supplementary Figure 29: full-sized scans of Western blots in Supplementary Figure 8D, E

Figure 9A

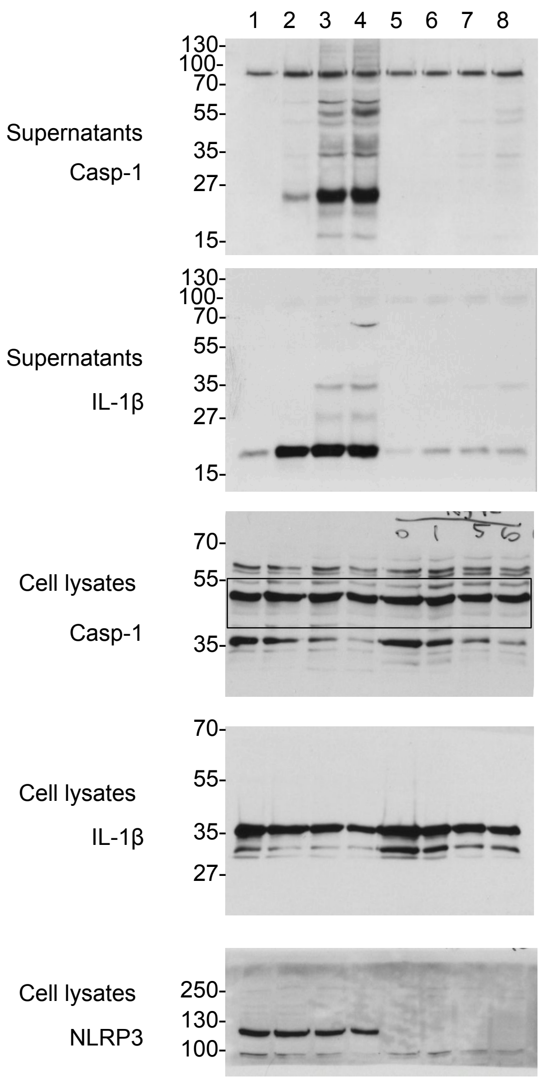

Figure 9B

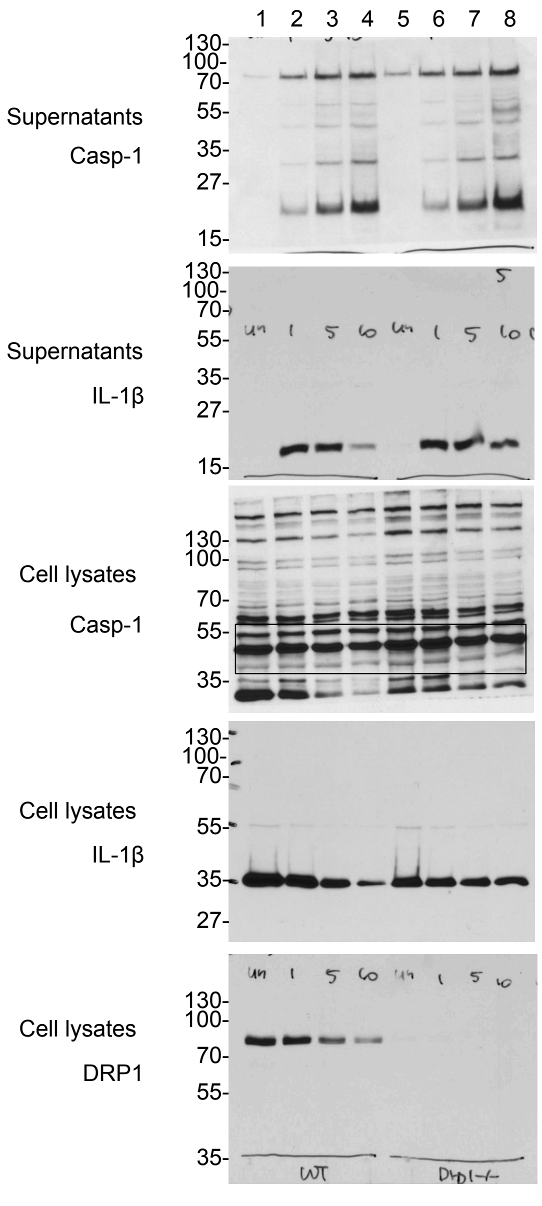

Supplementary Figure 30: full-sized scans of Western blots in Figure 9A, B

Figure 9C

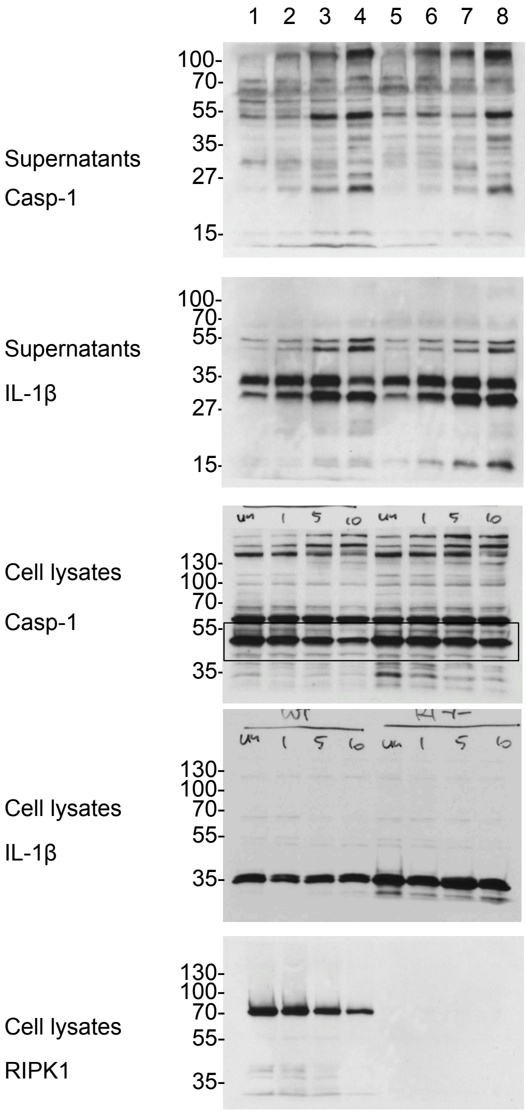

Figure 9D

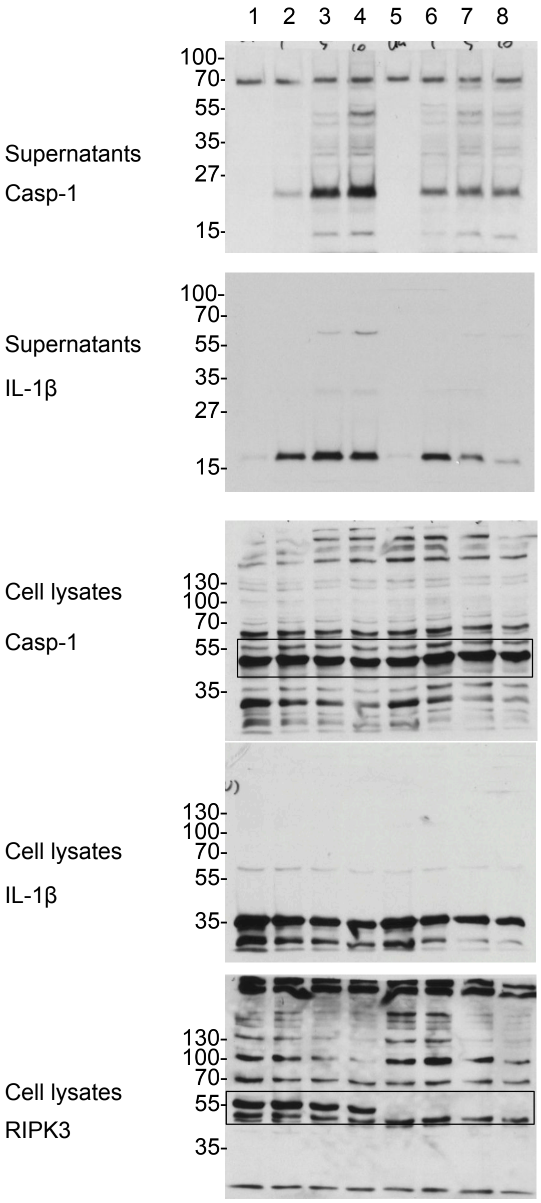

Supplementary Figure 31: full-sized scans of Western blots in Figure 9C, D

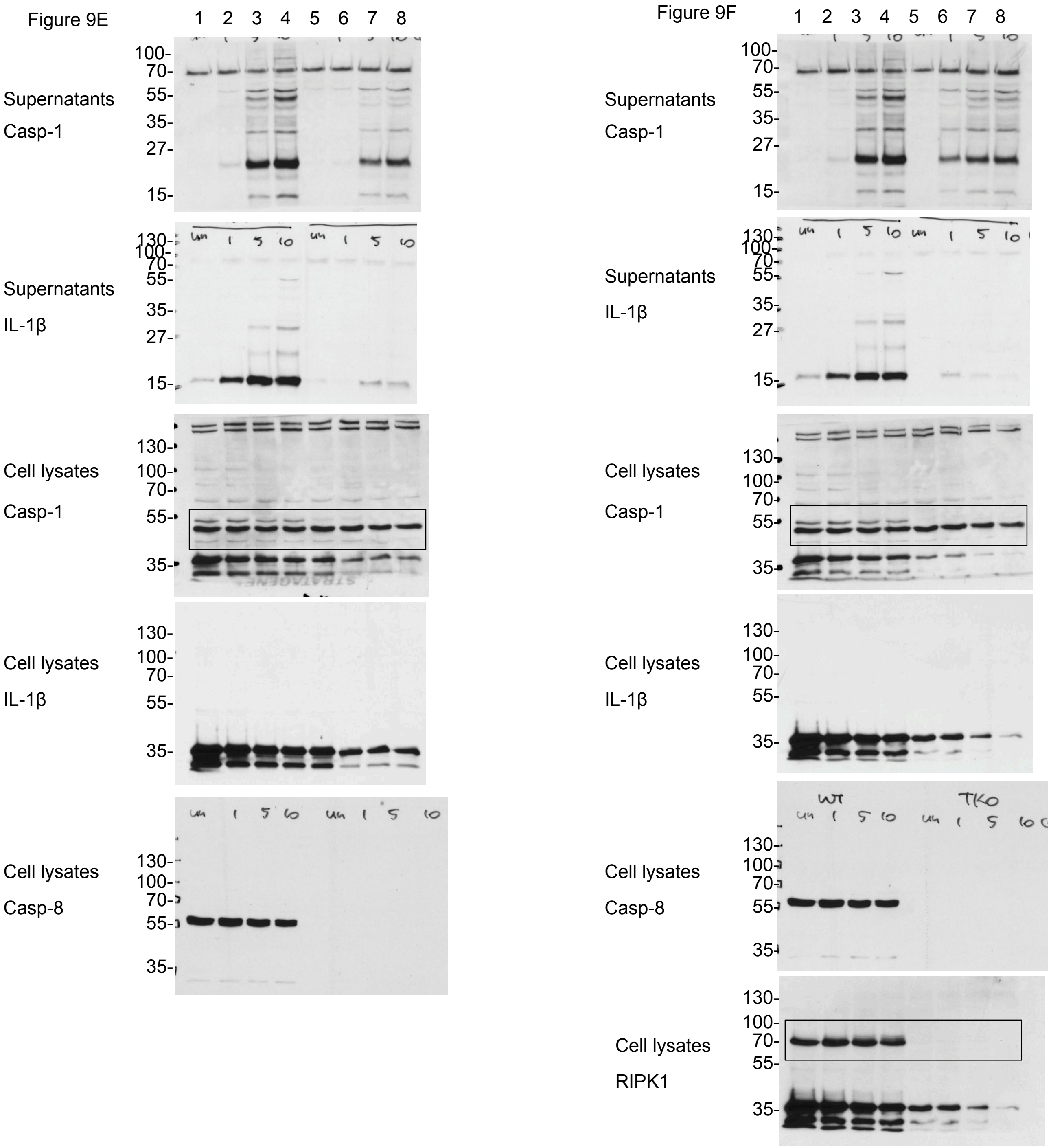

Supplementary Figure 32: full-sized scans of Western blots in Figure 9E, F

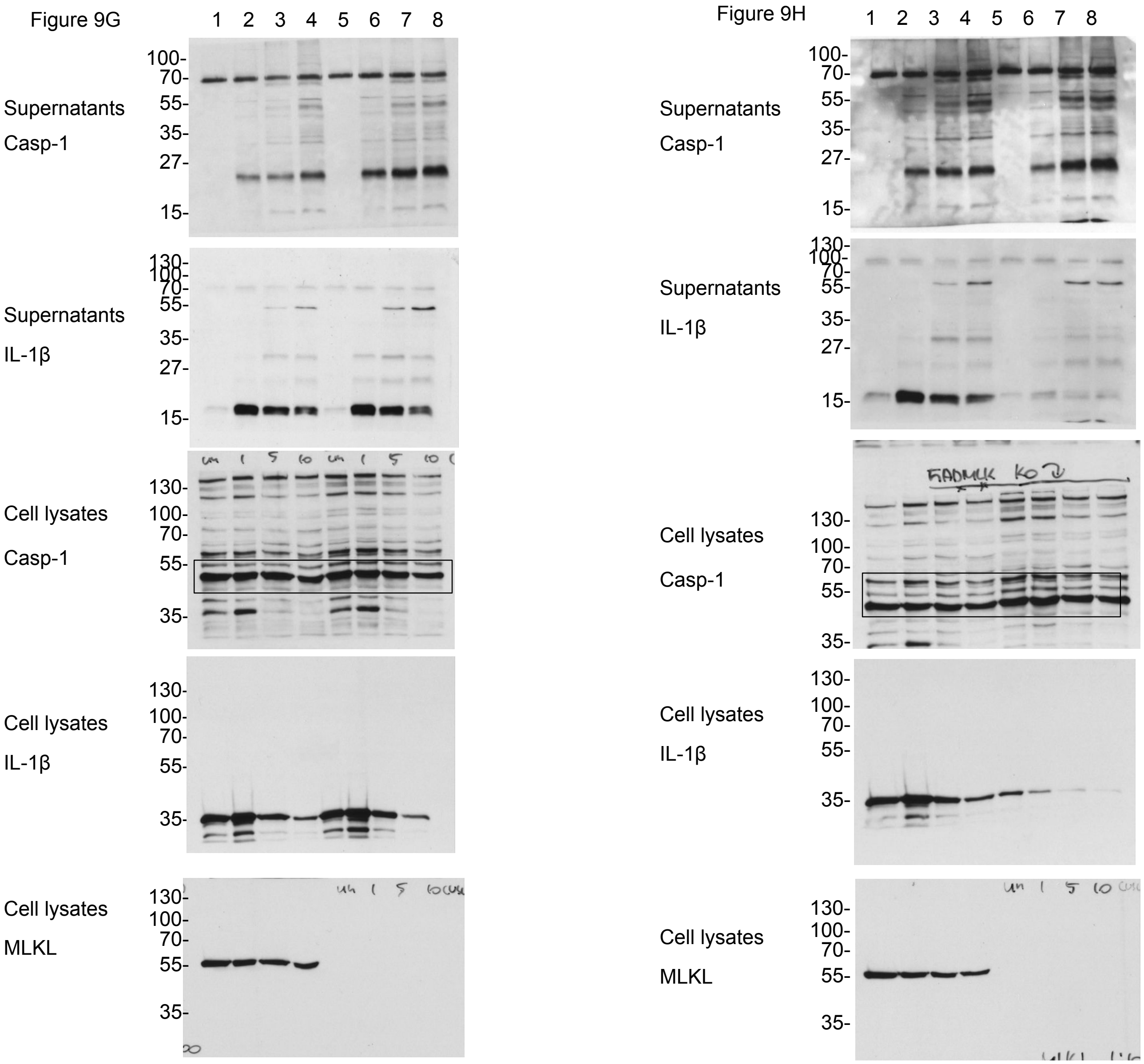

Supplementary Figure 33: full-sized scans of Western blots in Figure 9G, H

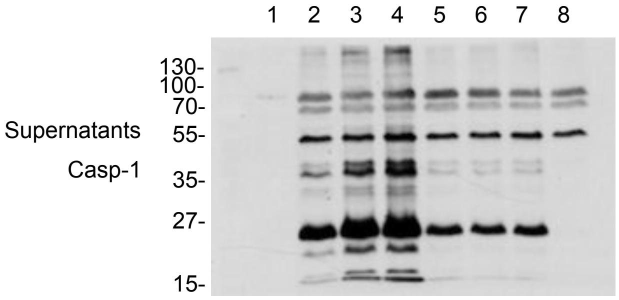

Supplementary Figure 34: full-sized scans of Western blots in Supplementary Figure 1

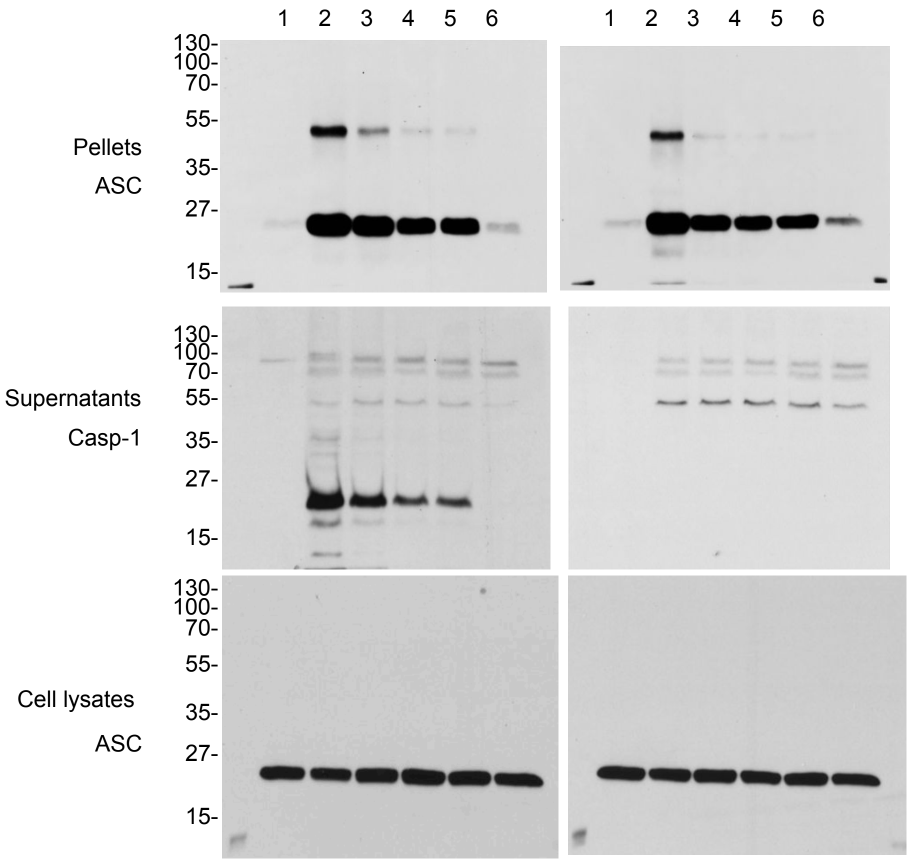

Supplementary Figure 35: full-sized scans of Western blots in Supplementary Figure 2A

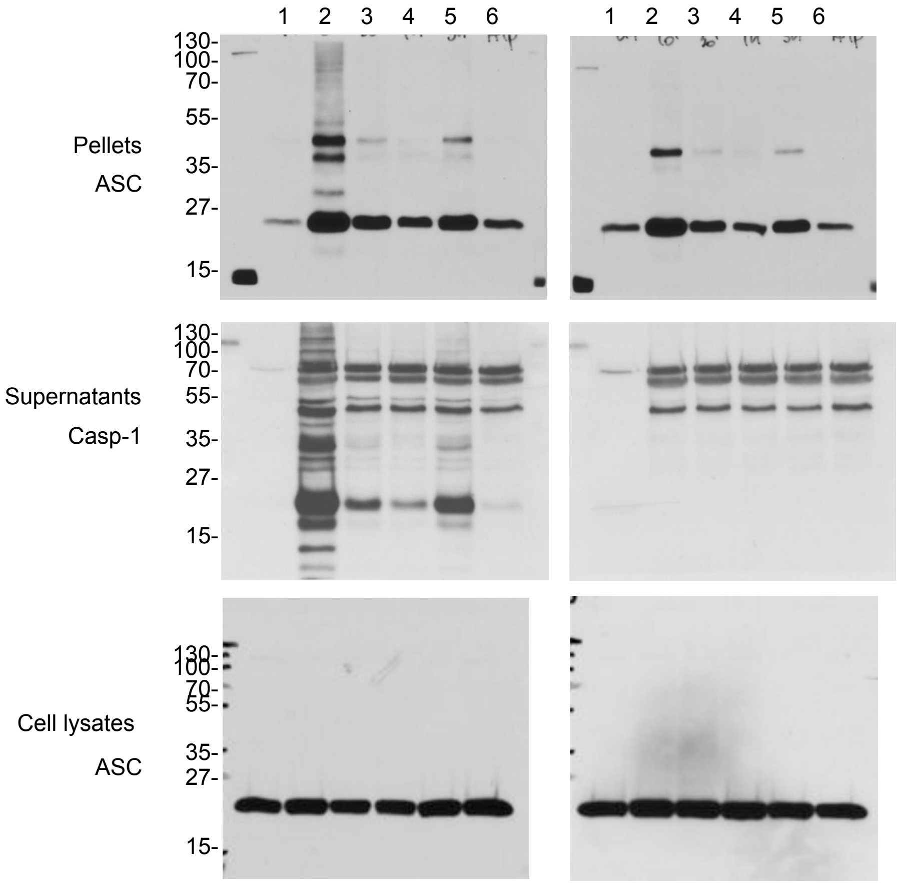

Supplementary Figure 36: full-sized scans of Western blots in Supplementary Figure 2B

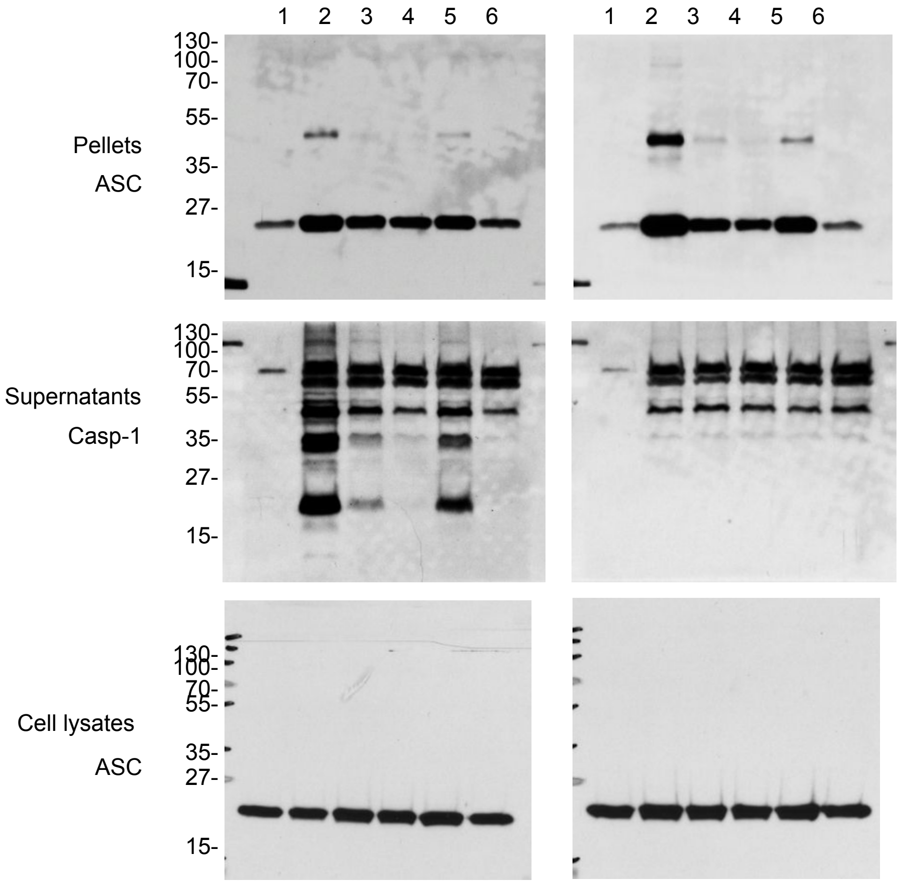

Supplementary Figure 37: full-sized scans of Western blots in Supplementary Figure 2C

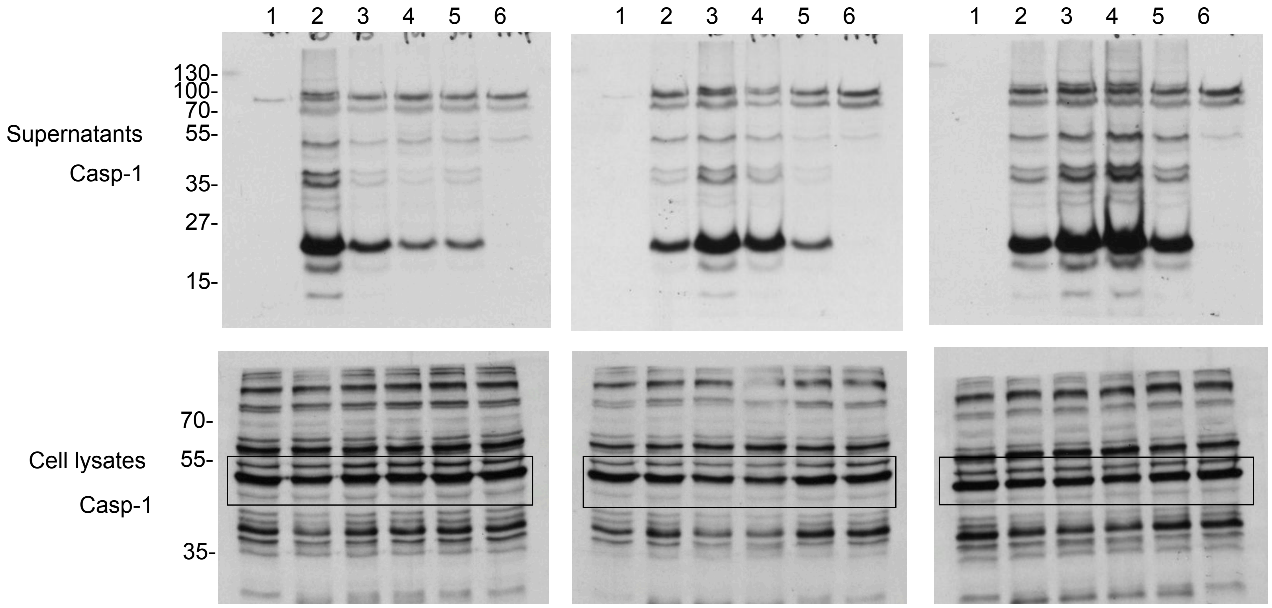

Supplementary Figure 38: full-sized scans of Western blots in Supplementary Figure 3A

Supplementary Figure 3B

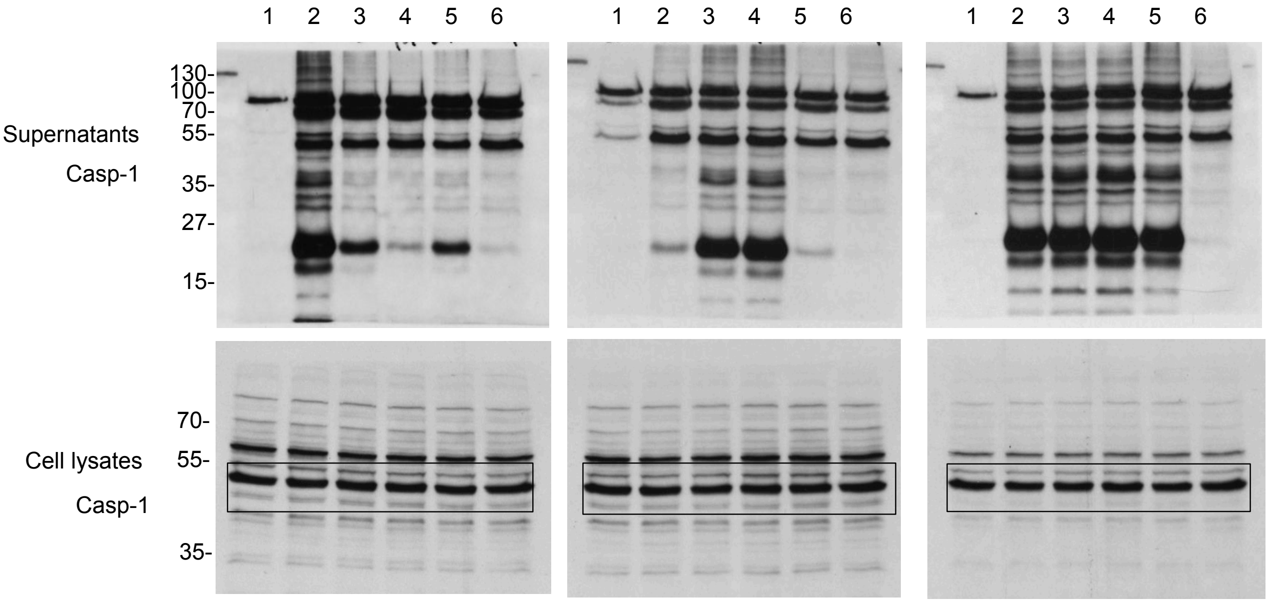

Supplementary Figure 3C

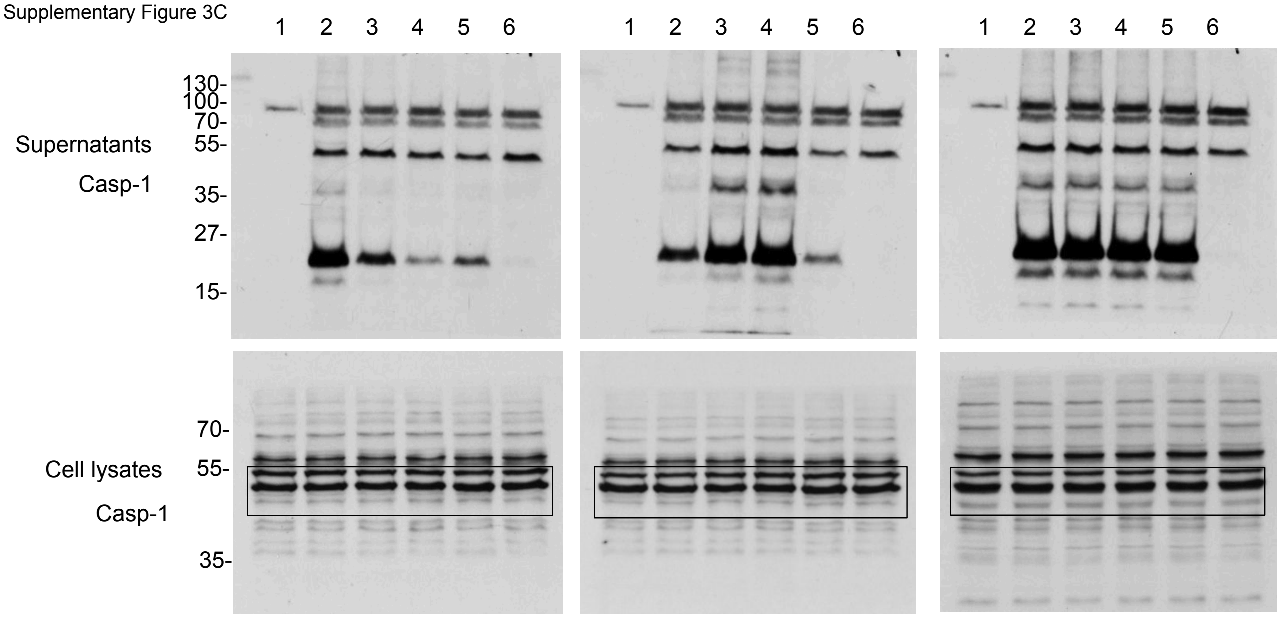

Supplementary Figure 39: full-sized scans of Western blots in Supplementary Figure 3B, C

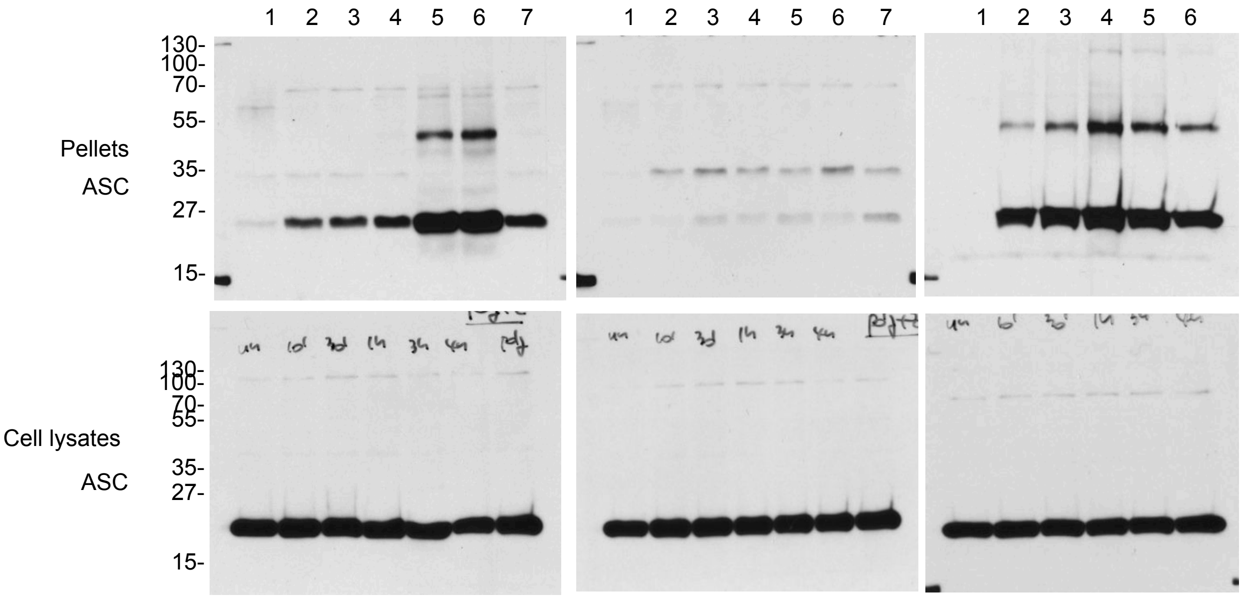

Supplementary Figure 40: full-sized scans of Western blots in Supplementary Figure 4A

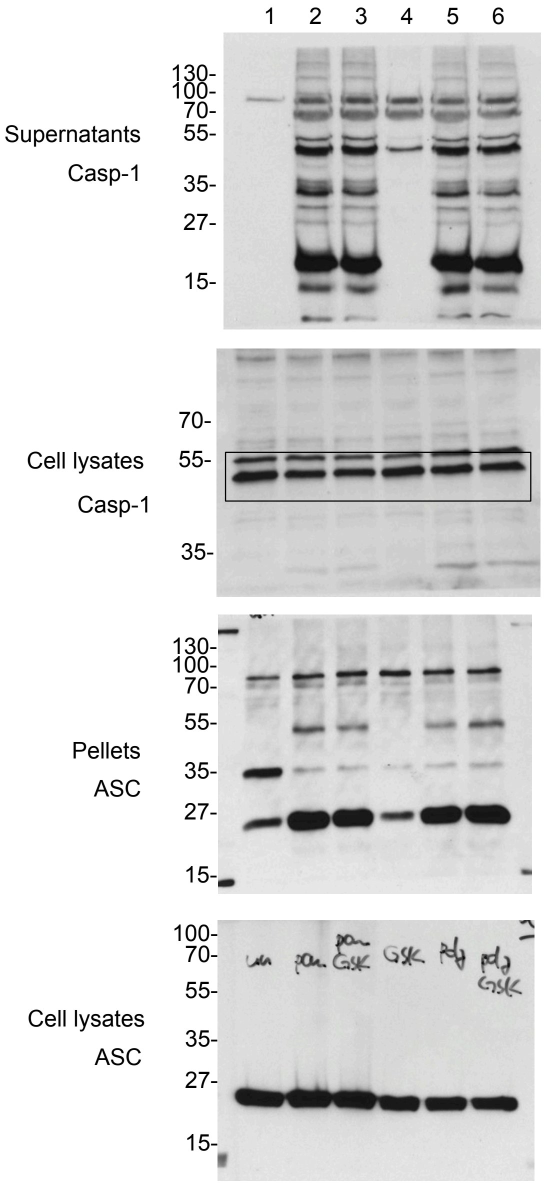

Supplementary Figure 41: full-sized scans of Western blots in Supplementary Figure 4B

Supplementary Figure 5A

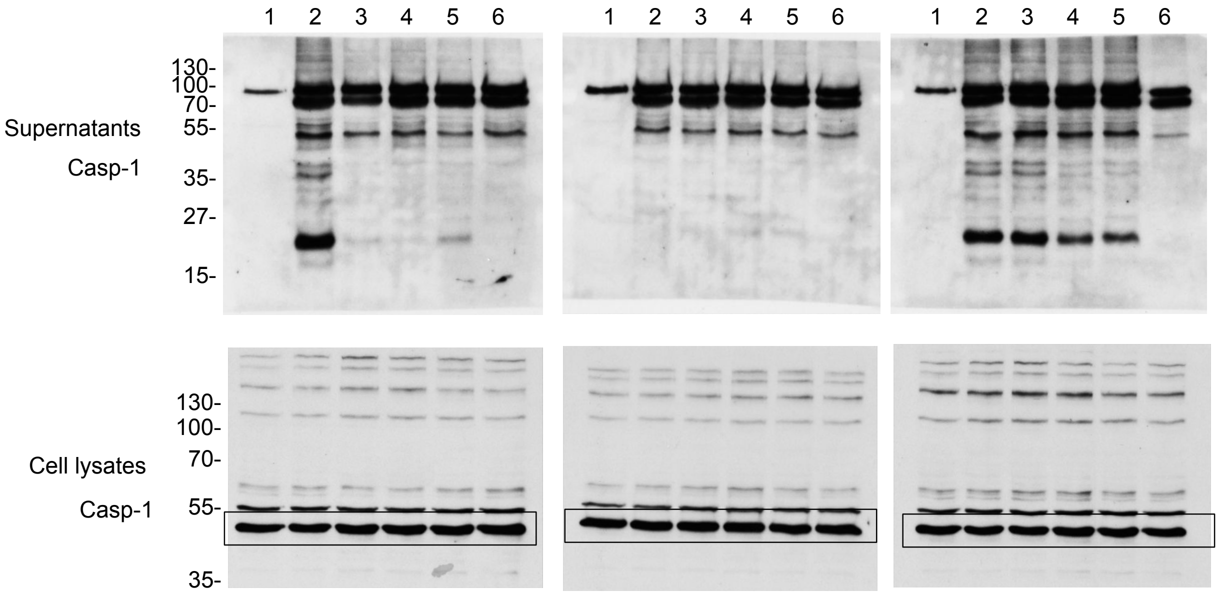

Supplementary Figure 5B

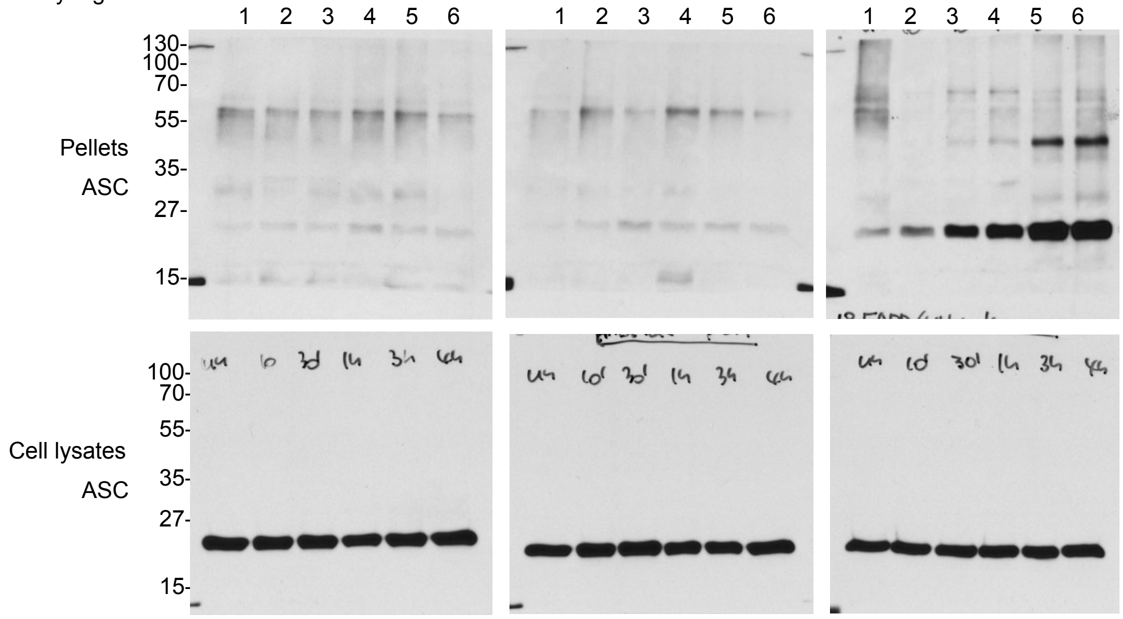

Supplementary Figure 42: full-sized scans of Western blots in Supplementary Figure 5A, B

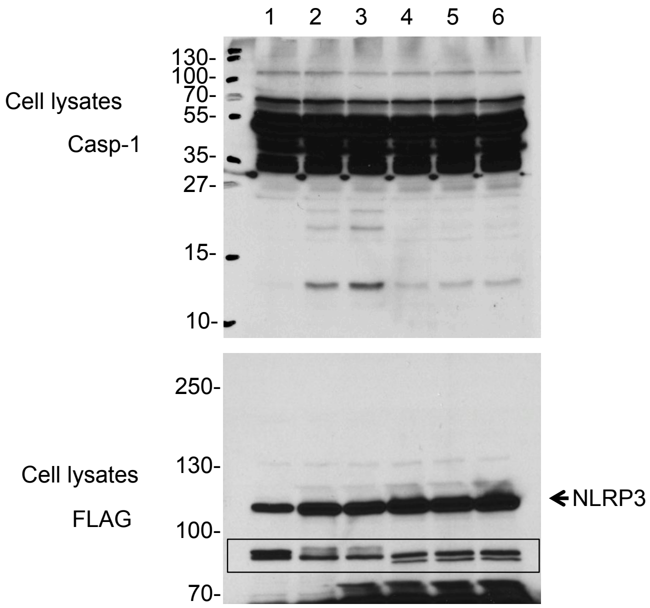

Supplementary Figure 43: full-sized scans of Western blots in Supplementary Figure 8

Supplementary Figure 9B

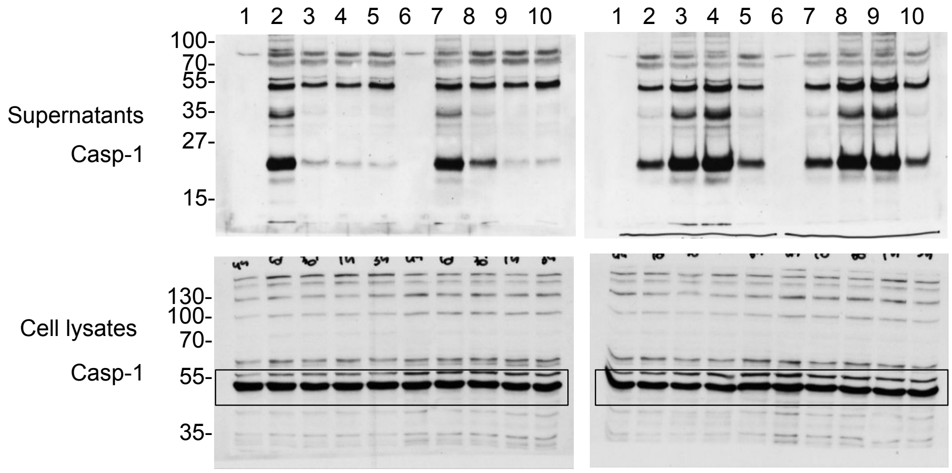

Supplementary Figure 9C

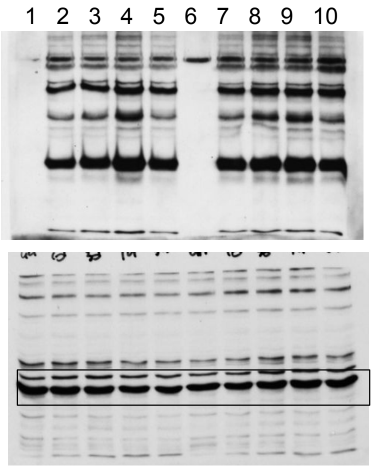

Supplementary Figure 9A

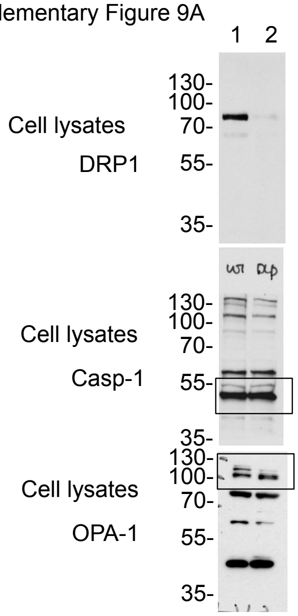

Supplementary Figure 9D

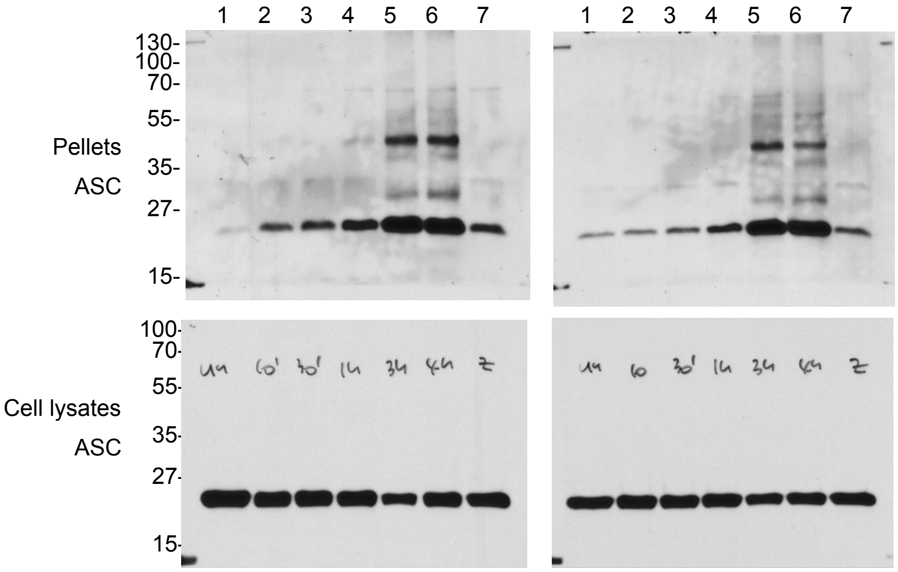

Supplementary Figure 44: full-sized scans of Western blots in Figure 9A-D

Supplementary Figure 10

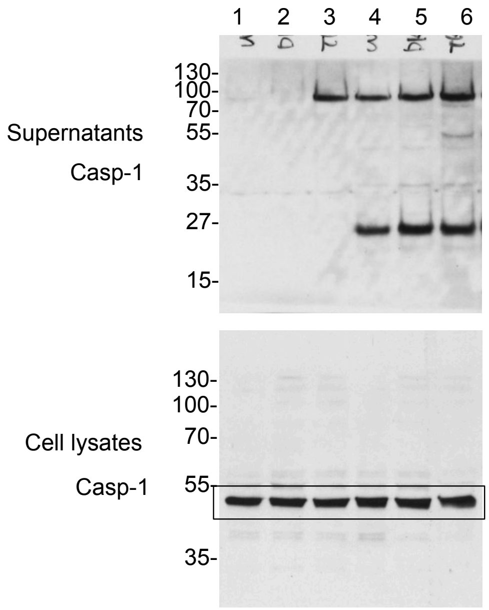

Supplementary Figure 11

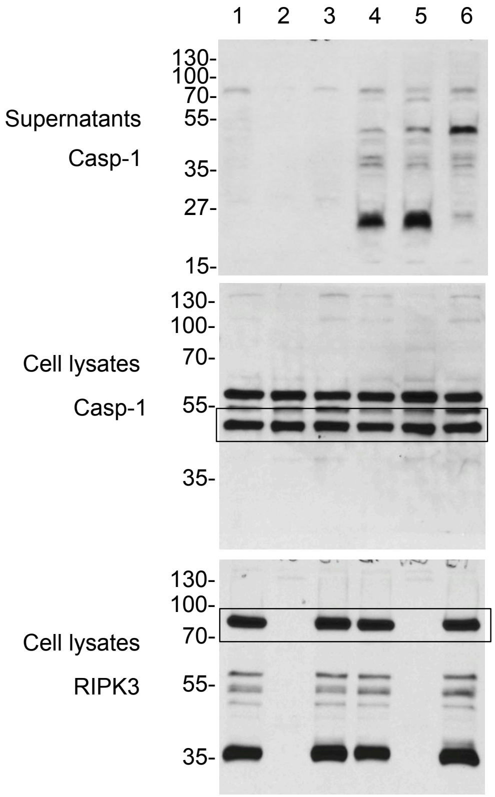

Supplementary Figure 45: full-sized scans of Western blots in Supplementary Figures 10 and 11

Supplementary Figure 12A

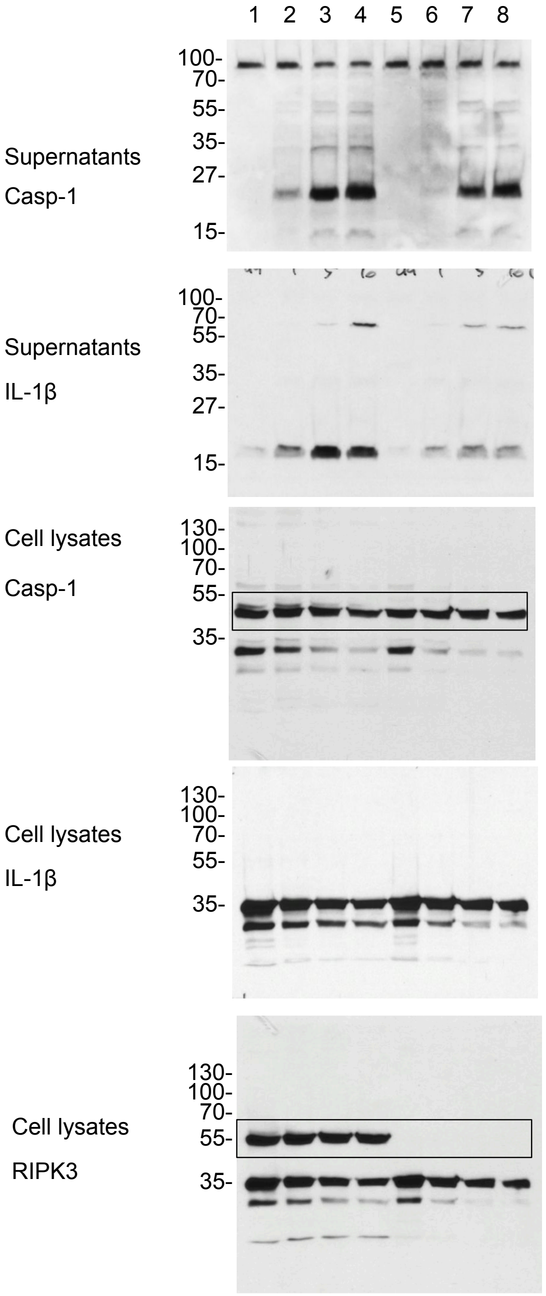

Supplementary Figure 12B

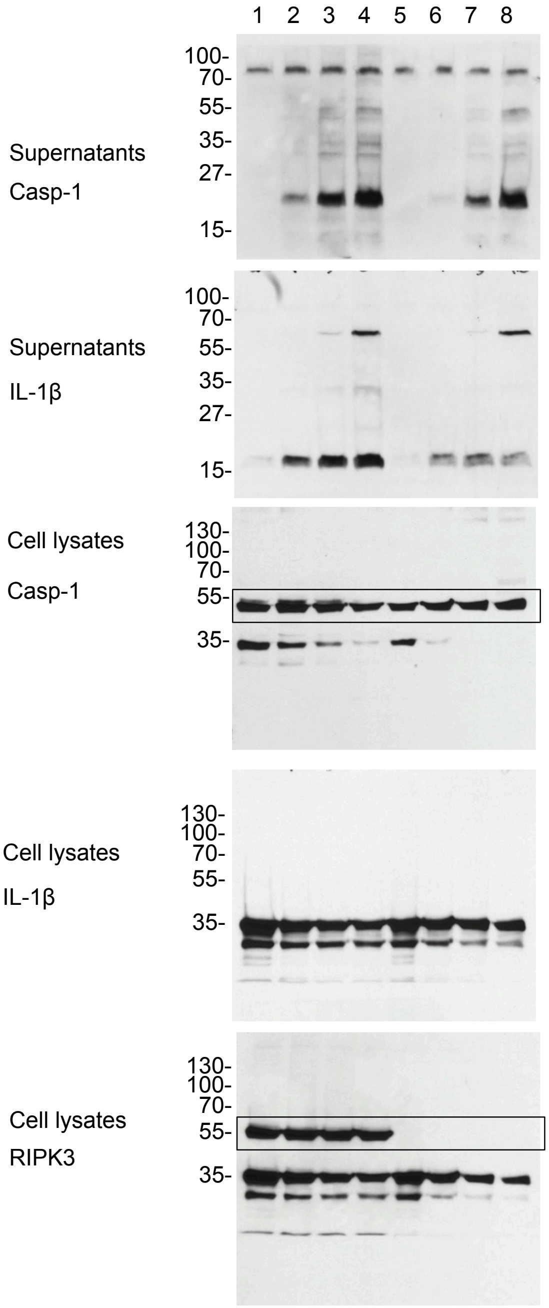

Supplementary Figure 46: full-sized scans of Western blots in Supplementary Figure 12A, B

Supplementary Figure 12C

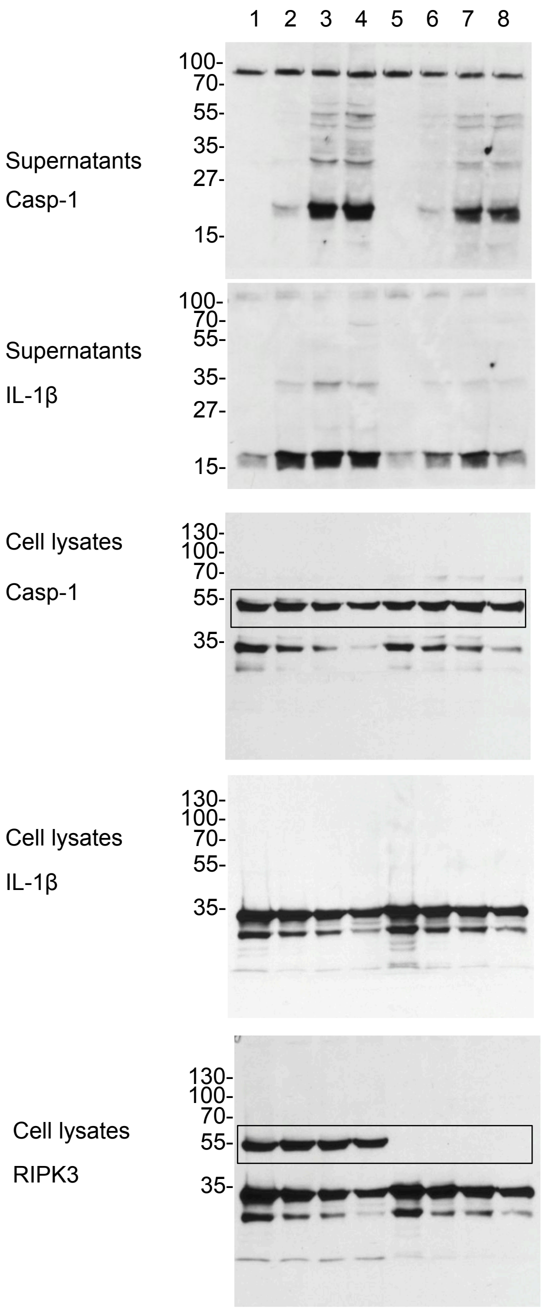

Supplementary Figure 47: full-sized scans of Western blots in Supplementary Figure 12C

Supplementary Figure 13A

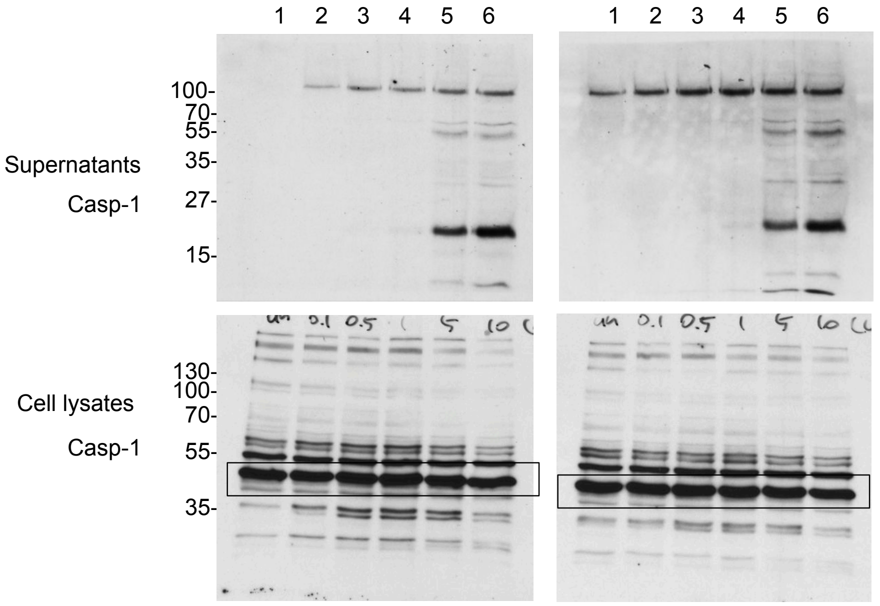

Supplementary Figure 13B

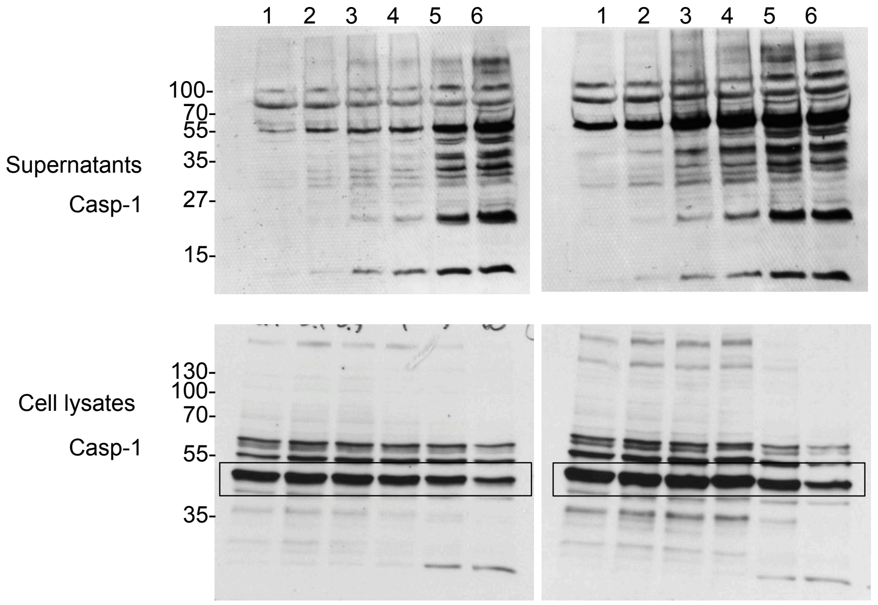

Supplementary Figure 48: full-sized scans of Western blots in Supplementary Figure 13A, B

Supplementary Figure 13C

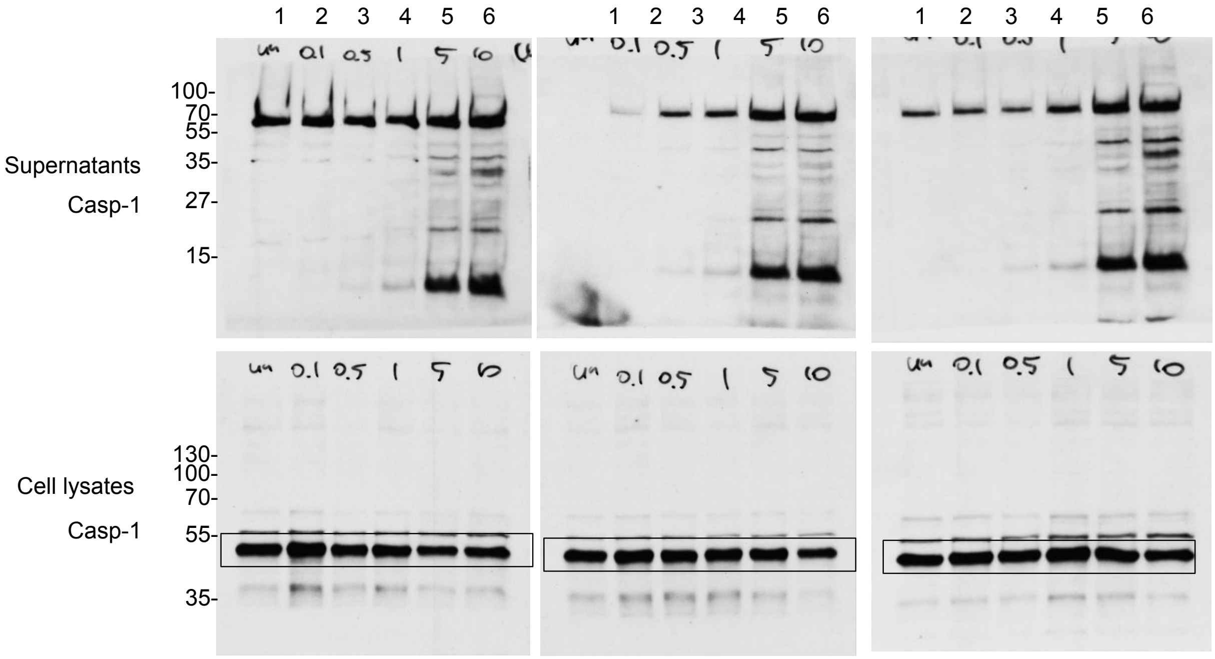

Supplementary Figure 49: full-sized scans of Western blots in Supplementary Figure 13C

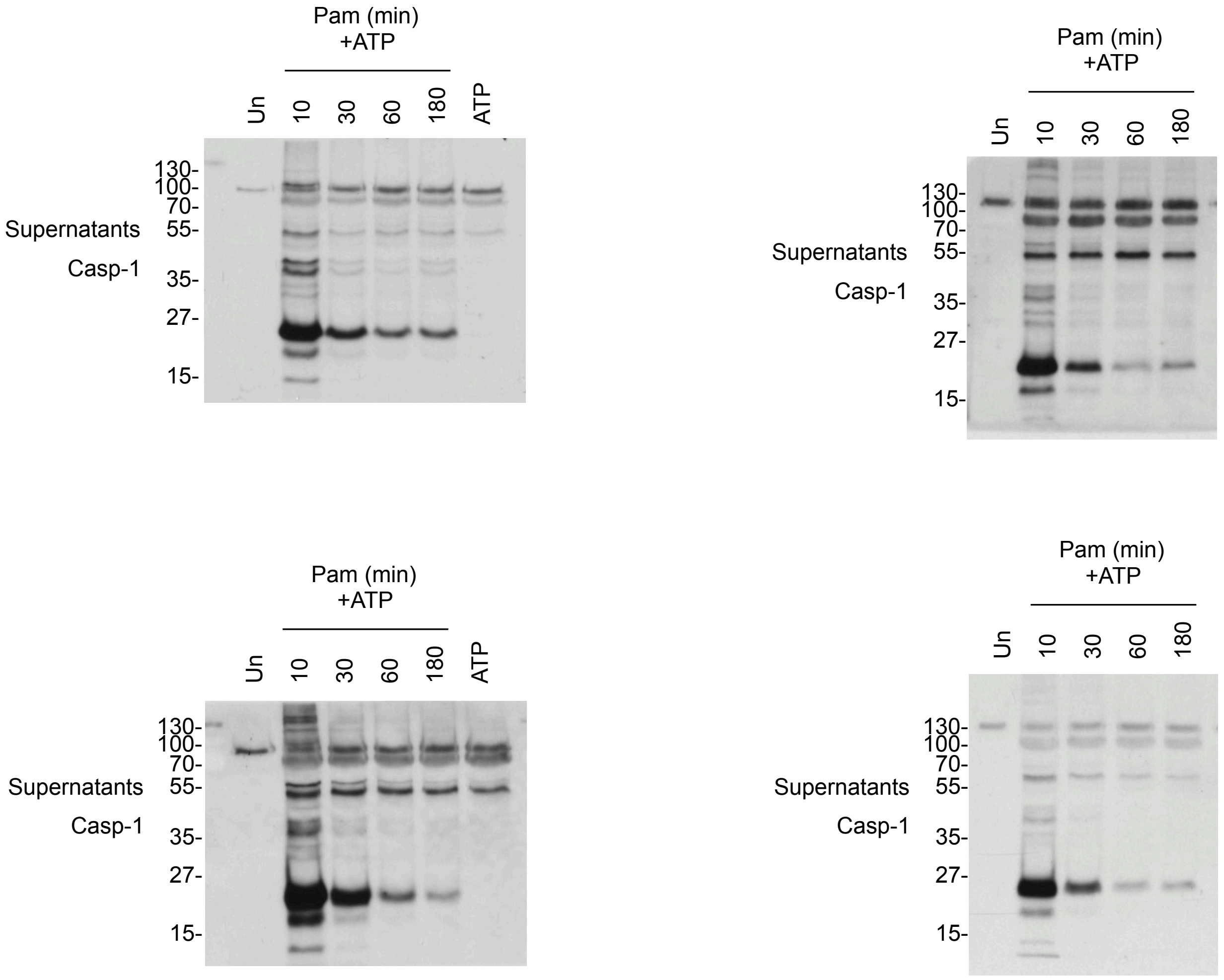

Supplementary Figure 50: Four independent repeats of the experiments in Figure 1A and Supplementary Figure 2A left panels . Samples were fractionated on a 12.5% SDS-gel and then western blotted with anti-caspase-1 antibody. Sup, culture supernatants.

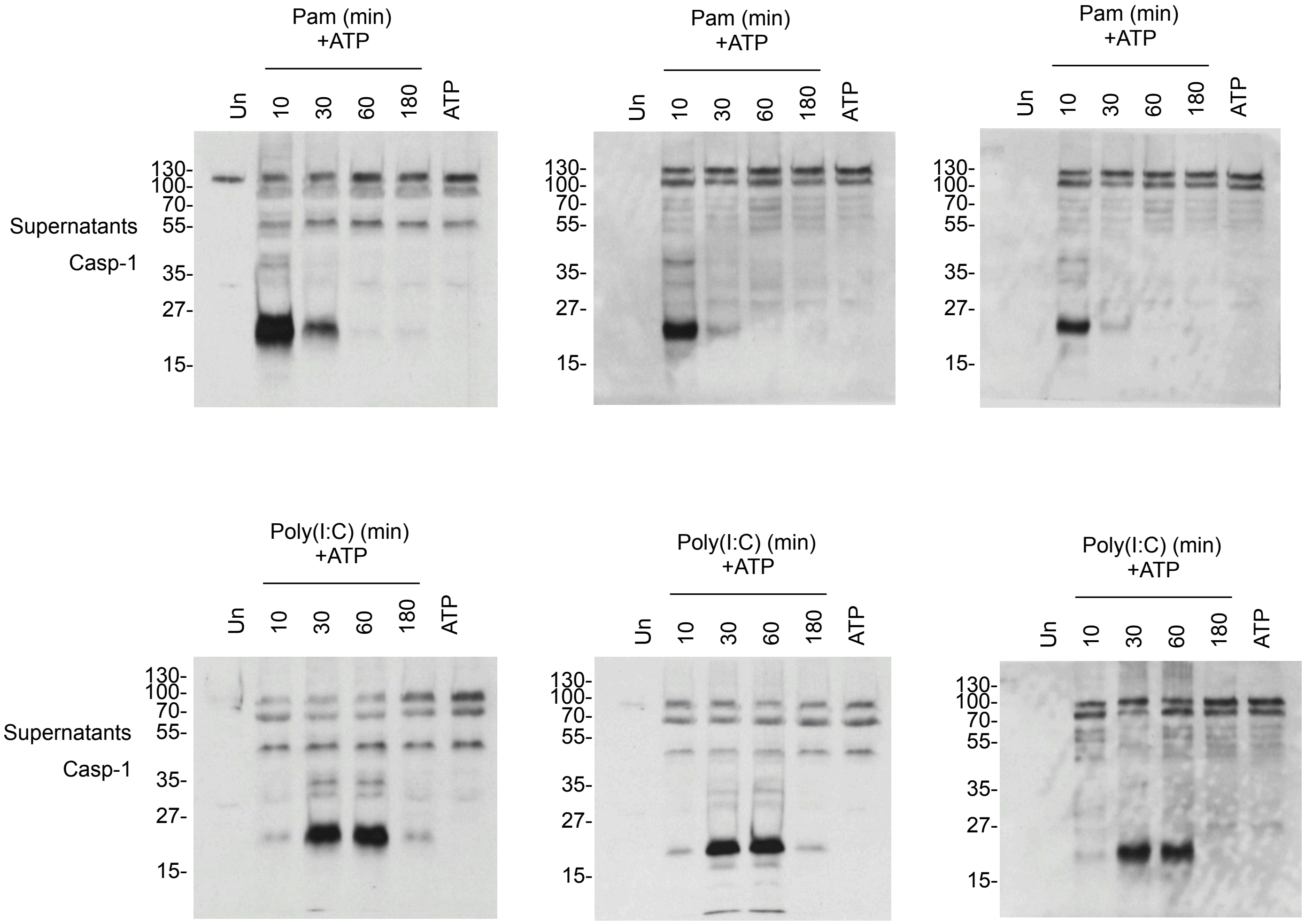

Supplementary Figure 51: Independent repeats of the experiments in Figure 1C left and middle panels . Samples were fractionated on a 12.5% SDS-gel and then western blotted with anti-caspase-1 antibody. Sup, culture supernatants.

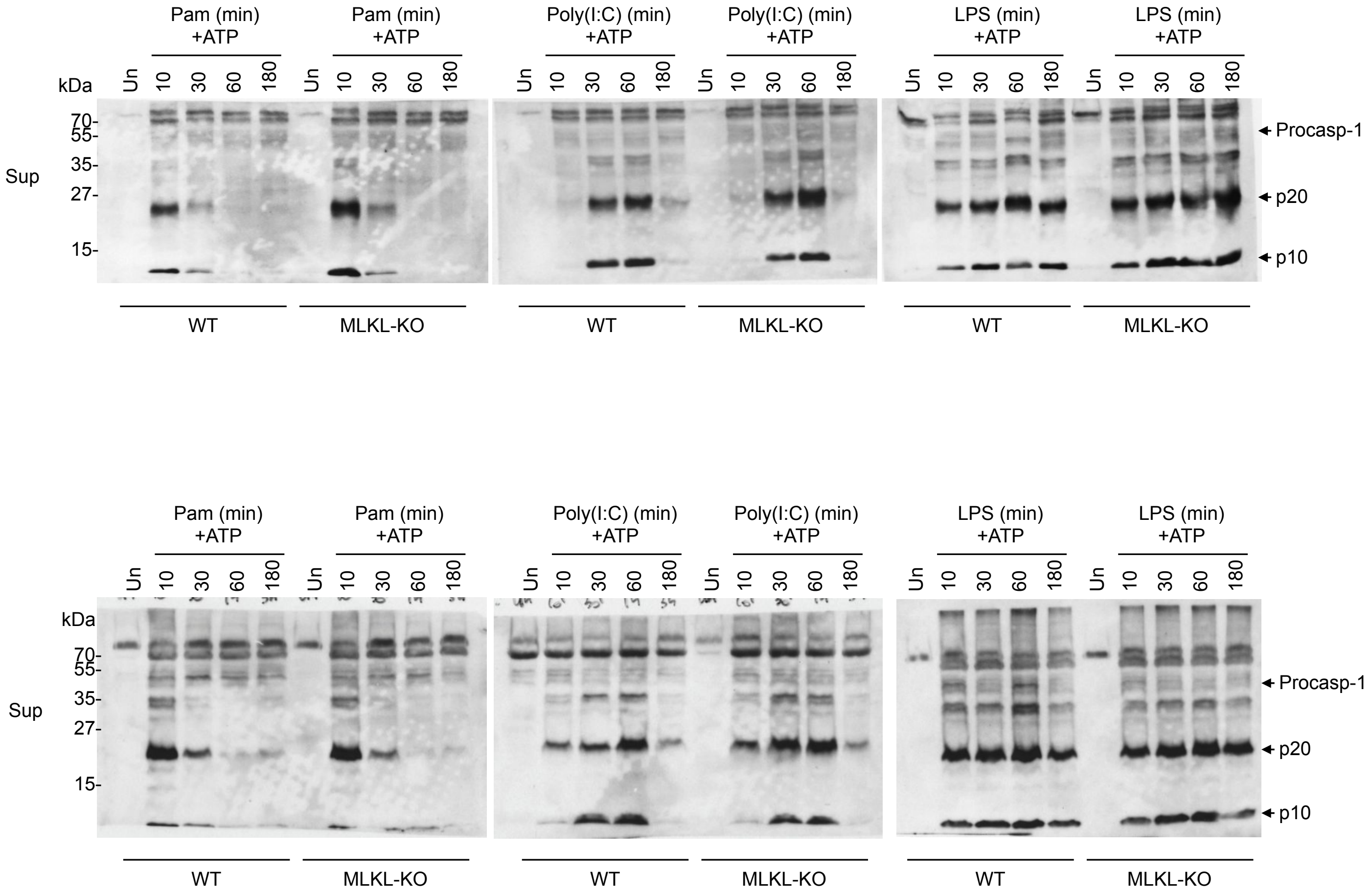

Supplementary Figure 52: Independent repeat of the experiments in Figure 6A. Samples were fractionated on a 12.5% SDS-gel and then western blotted with anti-caspase-1 antibody. Sup, culture supernatants.
